# Genomics-guided identification of potential modulators of SARS-CoV-2 entry proteases, TMPRSS2 and Cathepsins B/L

**DOI:** 10.1371/journal.pone.0256141

**Published:** 2021-08-18

**Authors:** Kartikay Prasad, Suliman Yousef AlOmar, Eman Abdullah Almuqri, Hassan Ahmed Rudayni, Vijay Kumar

**Affiliations:** 1 Amity Institute of Neuropsychology & Neurosciences (AINN), Amity University, Noida, UP, India; 2 Department of College of Science, King Saud University, Riyadh, Kingdom of Saudi Arabia; 3 Department of Biology, College of Science, Imam Mohammad Ibn Saud Islamic University, Riyadh, Saudi Arabia; 4 Biology Department, College of Science, Imam Muhammad bin Saud Islamic University, Riyadh, Kingdom of Saudi Arabia; North-Eastern Hill University, INDIA

## Abstract

SARS-CoV-2 requires serine protease, transmembrane serine protease 2 (TMPRSS2), and cysteine proteases, cathepsins B, L (CTSB/L) for entry into host cells. These host proteases activate the spike protein and enable SARS-CoV-2 entry. We herein performed genomic-guided gene set enrichment analysis (GSEA) to identify upstream regulatory elements altering the expression of TMPRSS2 and CTSB/L. Further, medicinal compounds were identified based on their effects on gene expression signatures of the modulators of TMPRSS2 and CTSB/L genes. Using this strategy, estradiol and retinoic acid have been identified as putative SARS-CoV-2 alleviation agents. Next, we analyzed drug-gene and gene-gene interaction networks using 809 human targets of SARS-CoV-2 proteins. The network results indicate that estradiol interacts with 370 (45%) and retinoic acid interacts with 251 (31%) human proteins. Interestingly, a combination of estradiol and retinoic acid interacts with 461 (56%) of human proteins, indicating the therapeutic benefits of drug combination therapy. Finally, molecular docking analysis suggests that both the drugs bind to TMPRSS2 and CTSL with the nanomolar to low micromolar affinity. The results suggest that these drugs can simultaneously target both the entry pathways of SARS-CoV-2 and thus can be considered as a potential treatment option for COVID-19.

## 1. Introduction

**Fig 1:**
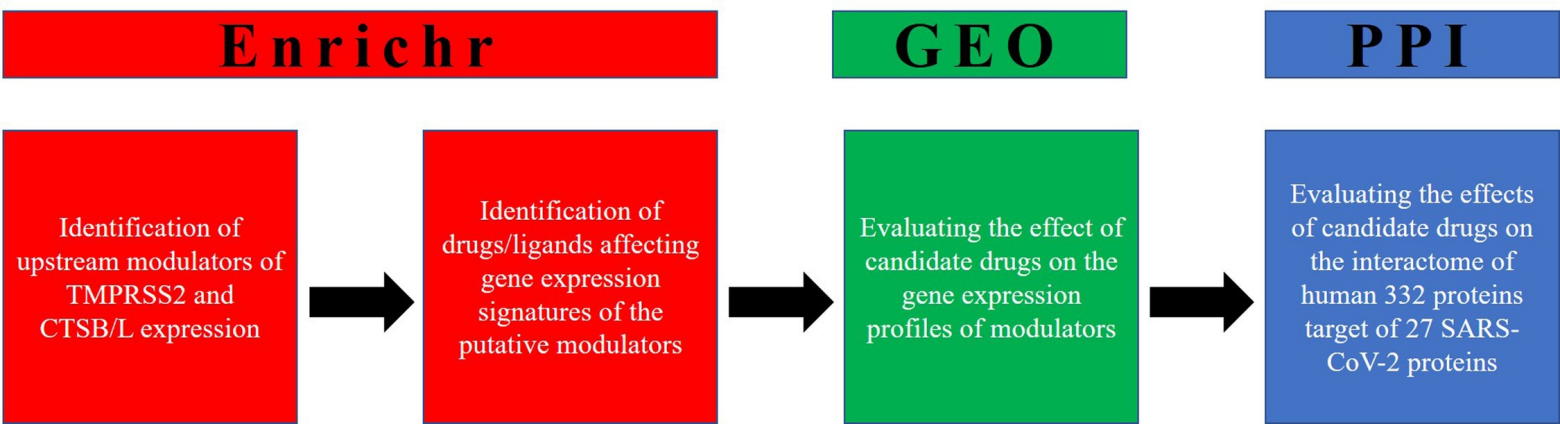
A strategic workflow describing the identification of candidate drugs targeting the regulators TMPRSS2 and CTSB/L identified through GSEA.

The recent outbreak of coronavirus disease 2019 (COVID-19) caused by severe acute respiratory 2 syndrome coronavirus 2 (SARS-CoV-2) has affected more than 180 million people with over 4.0 million deaths worldwide as of July 2021 (https://covid19.who.int/). SARS-CoV-2 enters the host cell through spike (S) protein which binds to angiotensin-converting enzyme 2 (ACE2) receptor present on the cell-membrane of host cells [1]. This binding is then followed by the cleavage of the S protein by the host transmembrane serine protease 2 (TMPRSS2) and then fusion to the host cell membrane [2]. Reportedly, furin protease is also likely involved in the SARS-CoV-2 infection process [3]. Also, many other studies indicate the involvement of the endosomal pathway as the key entry for SARS-CoV [4, 5]. These studies showed that the virus enters the host cell via pH- and receptor-mediated endocytosis pathways. In this pathway, the lysosomal cathepsins, mainly cathepsin L (CTSL) and cathepsin B (CTSB) cleaves and activate the S protein which then fuses with host cells [6, 7]. Very recently, Zhao et al. [8] reported that SARS-CoV-2 upregulates the expression of *CTSL* both in vivo and in vitro, which in turn enhances pseudo-virus infection in human cells. These studies largely suggest that CTSL may be considered as a drug target to treat COVID-19 infection [9, 10]. Therefore, the simultaneous co-expression of the host proteases, TMPRSS2 and CTSB/L in SARS-CoV-2 infected cells may lead to a higher risk for COVID-19 infection. Very recently, we utilized the network-based drug repurposing analyses to identify the possible common drugs that can target both the entry pathways. We have shown cyclosporine as a potential drug molecule, which binds not only with SARS-CoV-2 entry receptors, but is also predicted to interacts with most of SARS-CoV-2 target host genes, and thus could potentially inhibit the functions of SARS-CoV-2 proteins in human cells [11]. In extension to the previous study, we here performed genomic screening to find out the upstream regulatory elements modulating the expression of TMPRSS2, CTSB, and CTSL genes. The drugs and medicinal compounds were then identified based on their ability to change the gene expression of these regulatory elements. Using this strategy, we have identified estradiol and retinoic acid as potential drugs that could be repurposed to tackle COVID-19. Further, we utilized the network-based approach similar to our previous studies [11–13] to examine the drug-gene interactions between the drugs and 809 human genes that are being targeted by SARS-CoV-2 (**Fig 1**).

## 2. Methodology

### 2.1 Gene set enrichment analysis (GSEA) analysis of TMPRSS2 and CTSB/L

In this genomic-screen-based study, gene set enrichment analysis (GSEA) was performed to identify the genes linked to TMPRSS2 and CTSB/L for the substantial enrichment of different functional categories. Gene enrichment analysis offers information about the over-representation of a given gene in a particular pathway. GSEA was performed through Enrichr webserver (http://amp.pharm.mssm.edu/Enrichr [14, 15]. The predicted genes and transcription factors were further utilized for drugs and ligands identification based on their effects on gene expression signatures of the regulators of TMPRSS2 and CTSB/L genes. Two of the most promising candidate drugs, namely estradiol and retinoic acid manifest gene expression-altering activities of SARS CoV-2 entry genes.

The screening for enrichment was based on the “combined score” which is a product of the log of the p-value obtained from the Fisher’s exact test and the z-score, which is a deviation from the expected rank (i.e., combined score, c = log(p)*z,). Next for validating the GSEA result of the concerned genes, the available profiles of the gene expression were studied and also was created for other available SRAS-CoV-2 samples using the GEO2R tool of the NCBI Gene Expression Omnibus (GEO) database. GEO2R tool compares two or more groups of samples to identify the genes that are differentially expressed across experimental conditions. The tool in the background uses the limma package of R language to perform widely used statistical tests to identify the differentially expressed genes.

### 2.2. Drug-gene interaction network

Next, we have constructed the drug-gene interaction network from the predicted drug and their interactions with 809 human genes reported to interact with SARS-CoV-2 proteins from different affinity-based proteomics studies [16–18]. Cytoscape (version 3.8.2) [19] was used for preparing a drug-gene interaction network similar to our previous studies [11, 12, 20].

### 2.3. Molecular docking

For molecular docking, the three-dimension structure of CTSB (PDB ID: 1HUC), and CTSL (PDB ID: 4AXL) were obtained from Protein Data Bank [21]. The three-dimensional structure model of TMPRSS2 was obtained from the I-TASSER server [22] which is a threading-based hierarchal approach for structure prediction. The three-dimensional structures were energy minimized using the Swiss PDB Viewer (SPDBV) tool to attain the most stable conformation of the proteins [23]. Further, the mol file of Estradiol and Retinoic acid was downloaded from the DrugBank database [24]. The OpenBabel [25] software was used for converting the mol file into the three-dimensional structural file. The blind molecular docking was performed using AutoDock-Vina [26] with the grid spacing of 1Å and the exhaustiveness of 8. The docking results were ranked according to the binding affinity and root mean square deviation (RMSD) value. All the possible docked conformation and the protein-ligand interactions were visualized using PyMOL (https://pymol.org/2/) and Discovery studio [27].

## 3. Results

### 3.1. GSEA of genomic features associated with TMPRSS2 and CTSB/L

We have used the Enrichr bioinformatics platform [15] to find the genes modulating the expression level and functions of the TMPRSS2 and CTSB/L genes, thus potentially affecting SARS-CoV-2 infection. Expression profiling and GSEA of TMPRSS2 and CTSB/L genes revealed ubiquitous patterns of expression across human tissues. TMPRSS2 is highly expressed in the bladder, kidney, as well as gastrointestinal, and respiratory tract tissues. The CTSB/L genes are in general more homogeneously expressed across different tissues with the highest expression in the thyroid, salivary gland, and adipose tissues (**S1 Fig in S1 File**). TMPRSS2 as compared to CTSB/L has very low expression levels in brain and blood tissues.

GSEA of the COVID-19 related gene sets revealed that both TMPRSS2 and CTSL belong to SARS top 50 geneshot AutoRIF along with SARS 133 Literature-Associated Genes from Geneshot GeneRIF (**S2 Fig in S1 File and S1 Table**). The different GEO records revealed both the upregulation and downregulation of TMPRSS2 and CTSB/L genes by SARS-CoV-2 infection. Moreover, GEO records also suggest that the profile of expression largely depends on the type of cells (**S1 Table**).

**Fig 2:**
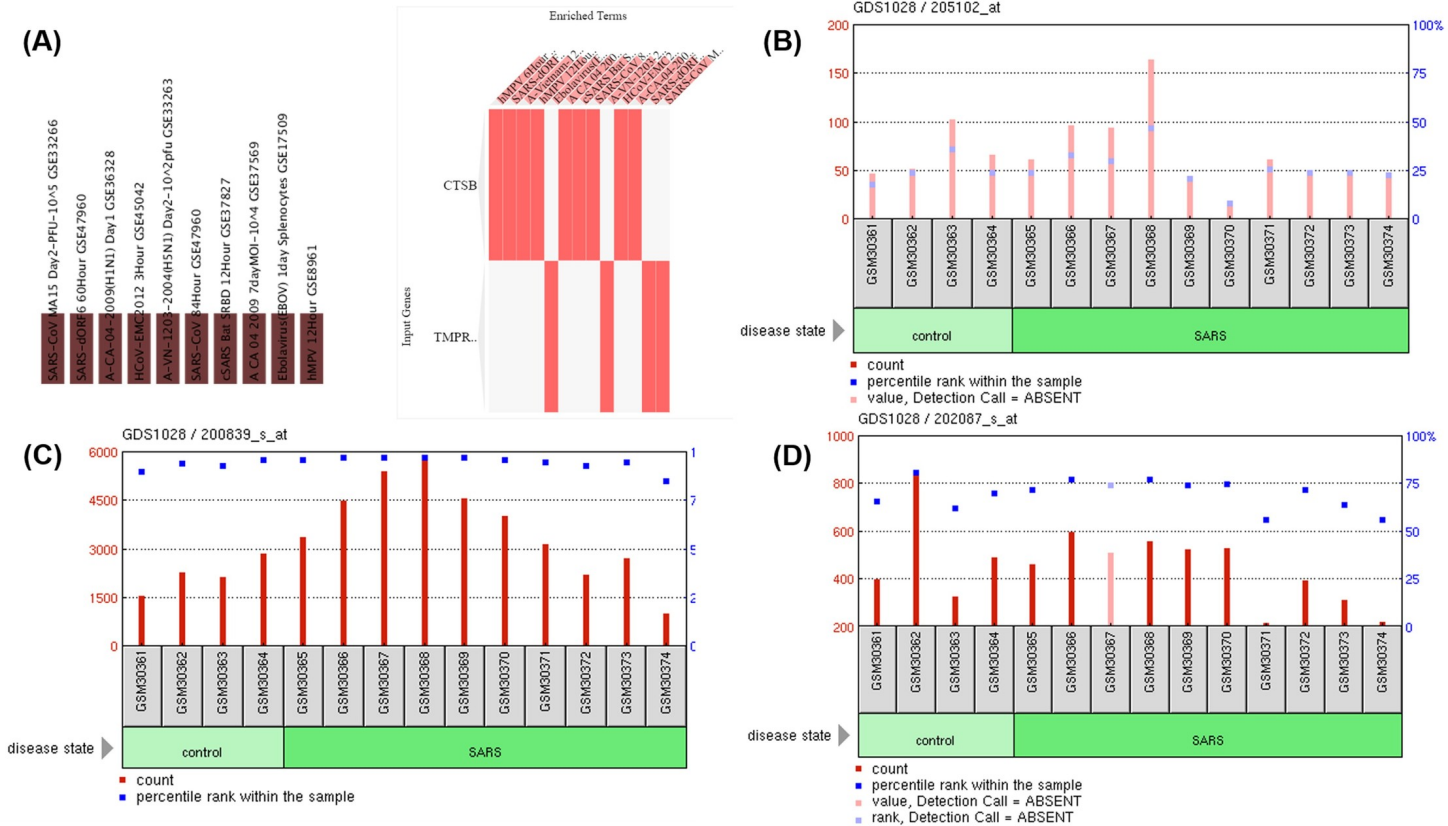
SARS-CoV2 effects on the expression of TMPRSS2 and CTSB/L genes. (A) GSEA of the virus perturbations from GEO focused on upregulated genes. Star in the figure represents the SARS-CoV-2 infection at 60 hr and 24 hr for TMPRSS2 and CTSB/L genes, respectively. GEO profiles of (B) TMPRSS2, (C) CTSB, and (D) CTSL expression in peripheral blood mononuclear cells (PBMCs) of patients with SARS infection (GSE1739) indicates the increased expression of these three genes involved in SARS CoV-2 entry.

The Enrichr reported the SARS-CoV infection challenge at 60 hr (GSE47960) as the highly enriched sets from the GEO records of upregulated genes during virus perturbations (**Fig 2**), and also marked by the upregulation of TMPRSS2 expression in human airway epithelial cells. Whereas CTSB showed upregulation in SARS-CoV infection after 24 hr (GSE47962) in human airway epithelial cells. The results indicate that SARS-CoV infection enhances the expression level of CTSB and TMPRSS2 genes after 1 day and 2.5 days, respectively (**Fig 2A and S1 Table**).

The findings were further substantiated by the augmented expression of TMPRSS2 and CTSB/L reported in the peripheral blood mononuclear cells (PBMCs) of patients with severe acute respiratory syndrome (**Fig 2B–2D and S3 Fig in S1 File**) [28].

GSEA identified common human disorders ranging from cancer to neurological diseases as enriched records through the upregulation of these genes (**S4 Fig in S1 File**), which matched with the different clinical comorbidities associated with COVID-19 infection. Interesting to note that both seasonal and pandemic H1N1 influenza virus infection highly increased the expression of TMPRSS2 and CTSL in human bronchial epithelial cells whereas, CTSB showed both increased and decreased expression (**S4 Fig in S1 File**).

Gene Ontology (GO) analyses revealed non-overlapping records of CTSB/L and TMPRSS2 (**S5 Fig in S1 File**). The common significantly enriched biological process for both genes include proteolysis and peptidase activity. The biological process for TMPRSS2 is significantly enriched in positive regulation of viral entry into the host cell (GO: 0046598) and protein auto-processing (GO:0016540), whereas, CTSB/L are enriched in the cellular response to thyroid hormone stimulus (GO:0097067) and cellular protein catabolic process (GO:0044257).

### 3.2. Identification of the transcription factors associated with the TMPRSS2 and CTSB/L genes

GSEA of the enriched records of transcription factors (TFs) using ChEA 2016 and ENCODE TF ChIP-seq 2015 databases reported different TFs for TMPRSS2 and CTSB/L genes (**S6 Fig in S1 File**). Common TFs for both TMPRSS2 and CTSB/L genes include PPARG (PPARγ) and SOX17 according to ChEA 2016. There were no common TFs identified through ENCODE TF ChIP-seq 2015 shared by these genes. However, for TMPRSS2 and CTSB, we found that PPARA (PPARα), RUNX1, and YAP1 binds to these two genes (**S6 Fig in S1 File**).

Next, the GSEA analysis was done to find out the involvement of PPARγ, PPARα, SOX17 RUNX1, and YAP1 in COVID-19 infection. The GSEA analysis reported the differential regulation of these genes in the COVID-19 gene sets available in the Enrichr platform (**S2 Table**). PPARα is downregulated by SARS-CoV-2 infection in intestinal organoids (GSE149312), in pancreatic organoids, and liver organoids (GSE151803). PPARγ has also been shown to be downregulated upon SARS-CoV-2 infection in intestinal organoids (GSE149312) and SARS-CoV infection in airway epithelium (GSE47961). In contrast, PPARγ is upregulated in COVID-19 patient PBMC and SARS-CoV infection in Vero E6 cells (GSE30589). Also, SOX17 and RUNX1 were upregulated in cardiomyocytes by SARS-CoV-2 infection (GSE150392). YAP1 also gets upregulated in COVID-19 patients BALF and SARS-CoV-2 infection in liver organoids (GSE151803). Interestingly, the expression of PPAR**γ** showed upregulation while PPARα and SOX17 showed mixed responses in the PBMC of patients suffering from severe acute respiratory syndrome (**S7 Fig in S1 File**). Furthermore, the microarray of the whole blood of COVID-19 patients (GSE166552) was analyzed through GEO2R. The results indicated that both PPARγ and PPARα are downregulated, while SOX17 and RUNX1 gets upregulated in COVID-19 patients (**S7 Fig in S1 File**).

**Fig 3:**
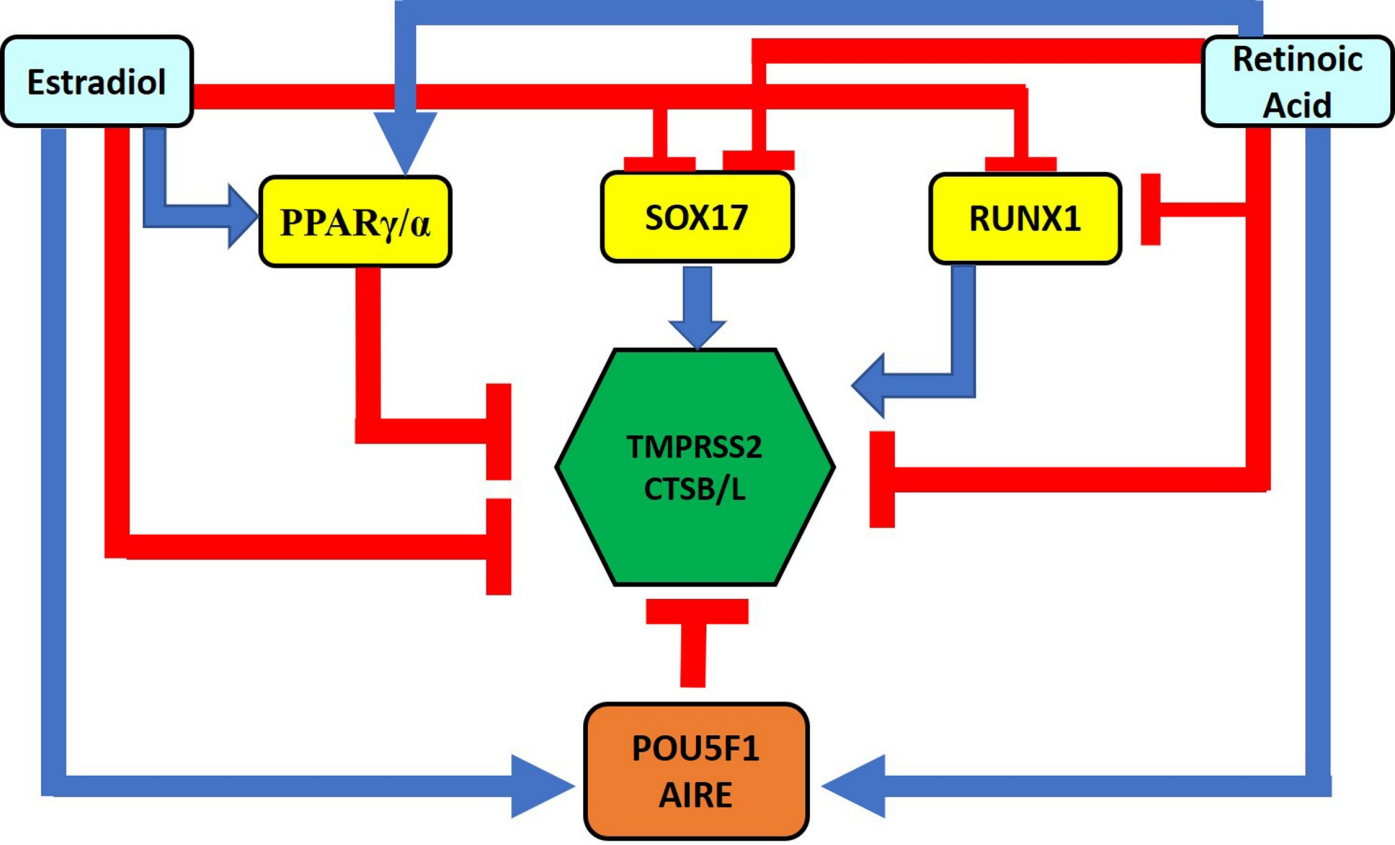
Summarized results of GSEA and GEO analysis. The figure represents PPARγ and PPARα as repressors while SOX17 as an activator of TMPRSS2 and CTSB/L gene expression. On the other hand, RUNX1 acts both as an activator and repressor of TMPRSS2 and CTSB/L genes. Two transcription factors, POU5F1 and AIRE downregulate the gene expression of TMPRSS2 and CTSB/L. The figure also depicts the effects of estradiol and retinoic acid on modulators of TMPRSS2 and CTSB/L expression. Both estradiol and retinoic acid upregulate the expression of PPARγ and PPARα, POU5F1 and AIRE, while downregulates the expression of SOX17 and RUNX1. Also, estradiol and retinoic acid downregulate the expression of TMPRSS2 and CTSB/L genes.

We also looked at the GEO database to study how these transcription factors modulate the expression of TMPRSS2 and CTSB/L genes. The GEO analysis indicate that PPARγ deficiency increased the expression of TMPRSS2 in induced inflammatory bowel disease (**S8 Fig in S1 File**). Moreover, dominant-negative expression of PPARγ decreased the expression of TMPRSS2 and CTSB/L genes, thus suggesting the repressor effects of PPARγ. Also, PPARα depletion increased the expression of TMPRSS2 and CTSB/L genes (**S8 Fig in S1 File**). The other transcription factor, SOX17 overexpression leads to the increased expression of TMPRSS2 and CTSB/L genes (**S8 Fig in S1 File**) suggesting the activation effects of SOX17. Similarly, the depletion of RUNX1 in human and mouse cells showed different effects on the expression of TMPRSS2 and CTSB/L genes. RUNX1 knockdown decreased the expression of TMPRSS2 in the human prostate cancer cell line, whereas increased the expression of CTSB/L genes (**S8 Fig in S1 File**). In contrast, RUNX1 depletion increased TMPRSS2 and CTSL expression, while decreased the expression of CTSB in mouse cells (**S8 Fig in S1 File**). The summary of these findings is summarized in **Fig 3**.

### 3.3. Identification of putative repressors of the TMPRSS2 and CTSB/L expression

GSEA of genomic databases was carried out to find putative modulators of TMPRSS2 and CTSB/L genes. The ARCHS4 transcription factor co-expression analysis indicated that the enriched records were demonstrating different patterns of co-expression with either TMPRSS2 or CTSB/L. Therefore, we look for the individual GSEA profiles for TMPRSS2 and CTSB, and TMPRSS2 and CTSL genes. The GSEA of the database involving the TF perturbations and the GEO perturbations based on upregulated genes revealed POU5F1 and AIRE as potential repressors of the expression of TMPRSS2 and CTSB; and TMPRSS2 and CTSL expression, respectively (**S9 Fig in S1 File**). These outcomes were further substantiated by observations that POU5F1 (also known as Oct4) knockdown significantly increased the expression of TMPRSS2 and CTSB/L in mouse embryonic stem cells (**S10 Fig in S1 File**). AIRE deficiency on the other hand increased the expression of TMPRSS2 and CTSB while decreased the expression of CTSL in mouse thymic epithelial cells (**S10 Fig in S1 File**). Both POU5F1 and AIRE genes have been mentioned in SARS133 literature-associated genes from Geneshot GeneRIF and also reported as up-regulated genes in COVID-19 infected bronchoalveolar lavage from patients (**S2 Table**) and in COVID-19 patient’s whole blood (GSE166552). A summary of the results is provided in **Fig 3**.

### 3.4. GSEA find Estradiol and Retinoic acid as potential drugs for mitigating COVID-19 infection

Further, GSEA of both the ligand and drug perturbations from GEO records of downregulated genes revealed estradiol and retinoic acid as the top highly enriched candidates (**S11 Fig in S1 File**). Estradiol seems to modulate both TMPRSS2 and CTSB/L gene expression levels, while retinoic acid appears to affect TMPRSS2 and CTSB expression (**S11 Fig in S1 File**). These observations, thus provide the initial evidence suggesting that both estradiol and retinoic acid could be considered as the candidates for drug repurposing against SARS-CoV-2 infection.

Manual curation of GEO data sets suggested that both estradiol and retinoic acid exert biological activities which leads to alleviating the SARS-CoV-2 infection. The administration of estradiol inhibits TMPRSS2, CTSB, and CTSL expression in human endothelial cells (**S12 Fig in S1 File**). Also, the administration of retinoic acid has resulted in significantly decreased expression of TMPRSS2 and CTSB/L genes in human endothelial cells (**S12 Fig in S1 File**).

Consistent with these findings, the investigation of GEO records revealed that estradiol appears to modulate several genes involved in promoting COVID-19 infection. Estradiol upregulated the expression of PPARγ, and PPARα, while downregulated the expression of SOX17 and RUNX1 genes (**S13 Fig in S1 File**). Furthermore, the interrogation of GEO records revealed that all-trans retinoic acid increased the expression of PPARγ and PPARα, while decreased the expression of SOX17 in human peripheral blood monocytes (**S13 Fig in S1 File**).

Estradiol upregulated the expression of POU5F1 in the mouse prostate gland (GSE3630). Also, the time course expression analysis of POU5F1 in mouse uterus response to 17beta-estradiol showed the upregulation for almost 4 hr (**S14 Fig in S1 File**). Estradiol also increased the expression of POU5F1 and AIRE in the endometrium of the Rhesus monkey (**S14 Fig in S1 File**). Retinoic acid also increased the expression of POU5F1 and AIRE in CD4+ T cells from spleen/lymph nodes of the mouse (**S14 Fig in S1 File**). Also, retinoic acid showed a mixed response for the expression of AIRE in human peripheral blood monocytes and MCF-7 breast cancer cells, suggesting different mechanisms of regulation. **Fig 3** summarizes the overall effects of estradiol and retinoic acid on the modifiers as well as on TMPRSS2 and CTSB/L.

### 3.5. Drug-gene interactions network of estradiol and retinoic acid in SARS-CoV2-human interactome

**Fig 4:**
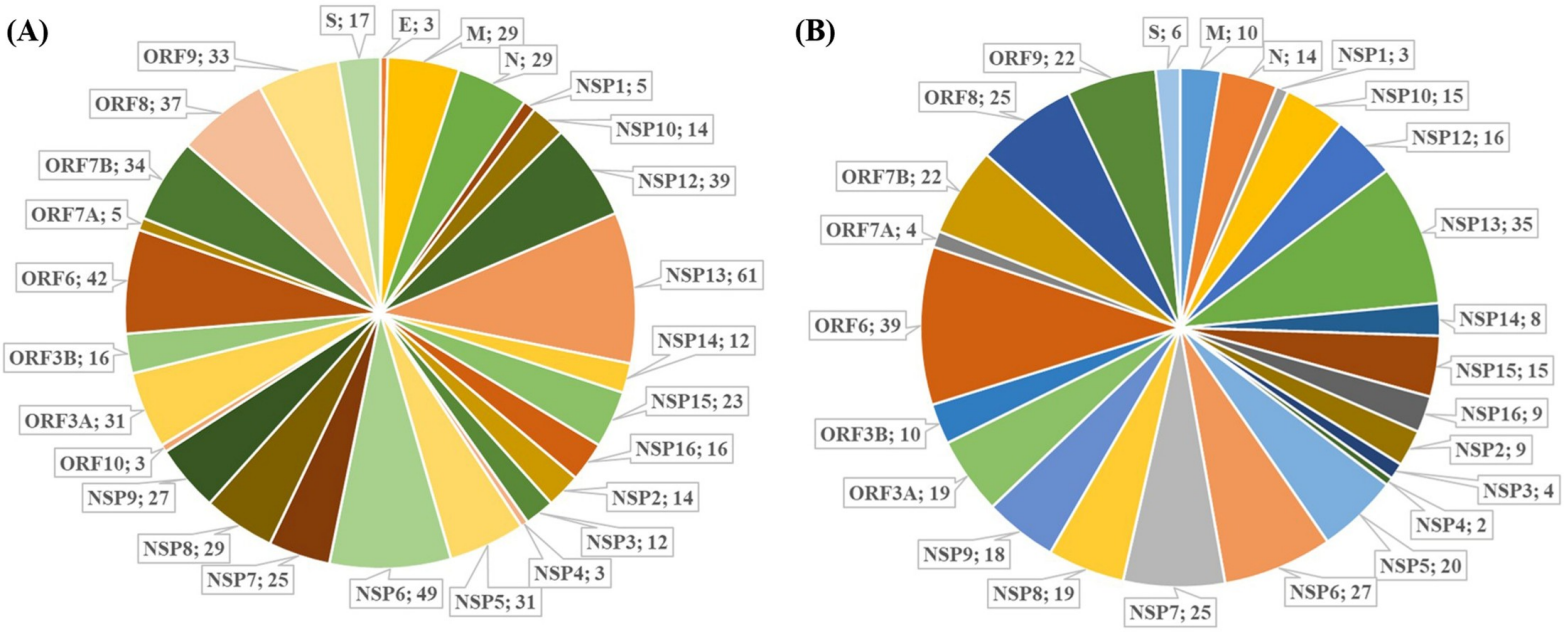
Effects of estradiol and retinoic acid on the drug-gene interactions network of SARS-CoV-2-human interactome. (A) Estradiol interacts with 370 (i.e., ~45%) of 809 human proteins targeted by SARS-CoV-2, making 637 interactions in total, thus possibly affecting the functions of all the SARS-CoV-2 protein in human cells. (B) Out of 809 SARs-CoV-2 human target proteins, retinoic acid interacts with 251 (i.e ~31%) proteins, making 395 interactions in total, affecting the functions of 96.2% SARS-CoV-2 protein in human cells.

SARS-CoV-2-human interactome from three different proteomics studies [16–18] identified a total of 809 human proteins significantly targeted by 27 SARS-CoV-2 proteins. Here, we report that estradiol interacts with 370 of 809 (45%) human proteins, and theoretically interferes with all of the SARS-CoV-2 proteins (**Fig 4A**). Most of these 370 human proteins interact with more than one SARS-CoV-2 protein. These 370 proteins make 809 interactions with 27 SARS-CoV-2 proteins with maximum interactions to NSP13 (61), NSP (49), ORF6 (42), and NSP12 (39) protein (**Fig 4A**), suggests an extensive protein-protein interactions network that can be significantly modulated by estradiol.

Similarly, retinoic acid interacts with 251 out of 809 (31%) human proteins targeting SARS-CoV-2 and interfering with the activities of 26 of 27 (96.2%) SARS-CoV-2 proteins (**Fig 4B**). These 251 human genes interact with SARS-CoV-2 proteins and make a total of 395 interactions with maximum interactions observed in case to ORF6 (39), NSP13 (35), NSP6 (27), NSP7 (25), and ORF8 (25) protein. Thus, estradiol and retinoic acid manifest significant interference with the SARS-CoV-2-human interactome. Remarkably, estradiol and retinoic acid in combination interacts with 461 of 809 (~56%) human proteins and thus affect the functions of all SARS-CoV-2 proteins (**S15 Fig in S1 File).**

### 3.6 Identification of potential miRNAs involved in the regulation of TMPRSS2 and CTSB/L

miRNAs can be utilized as an essential antiviral tool. To evaluate possible miRNAs that can directly regulate TMPRSS2 and CTSB/L, TargetScan microRNA 2017 from Enrichr has been explored. The analysis yielded no common miRNAs against these three genes (**S16 Fig in S1 File**). However, three miRNAs have been identified to potentially bind against TMPRSS2 and CTSL genes. Out of this, one of the human miRNA, hsa-miR-379 increased upon infection with influenza A infection in dendritic cells and peripheral blood of Parkinson’s disease patients (**S16 Fig in S1 File**). This miRNA has also been predicted to bind ORF10 of SARS-CoV-2 [29] and might be considered useful against SARS-CoV-2 infection.

### 3.7 Molecular docking of estradiol and retinoic acid to TMPRSS2 and CTSB/L genes

**Fig 5:**
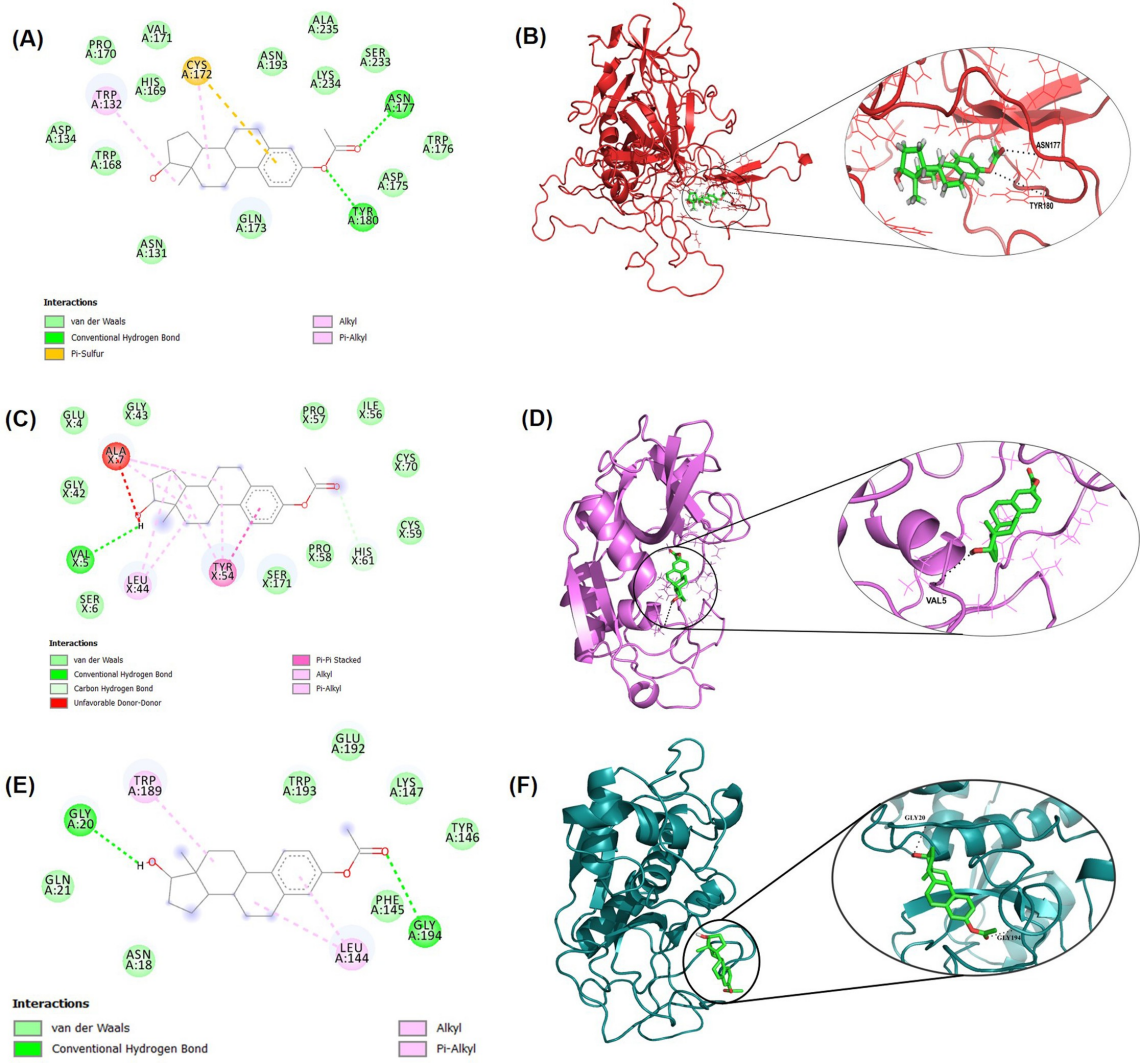
Molecular docking study of estradiol to TMPRSS2, CTSB, and CTSL. (A) Two-dimensional (2D) diagrams of TMPRSS2-estradiol interactions using Ligplot+. The figure provides the information about the interactions, the protein residues and interactions are colored accordingly. (B) The best docking pose in the three-dimensional (3D) structure of the protein. The black dotted line denotes the intermolecular hydrogen bond interactions. Similarly, (C) 2D diagrams of CTSB-estradiol interactions and (D) binding poses in the 3-D structure are shown. (E) and (F) represents 2-D interactions for CTSL-estradiol and 3-D binding poses, respectively.

**Fig 6:**
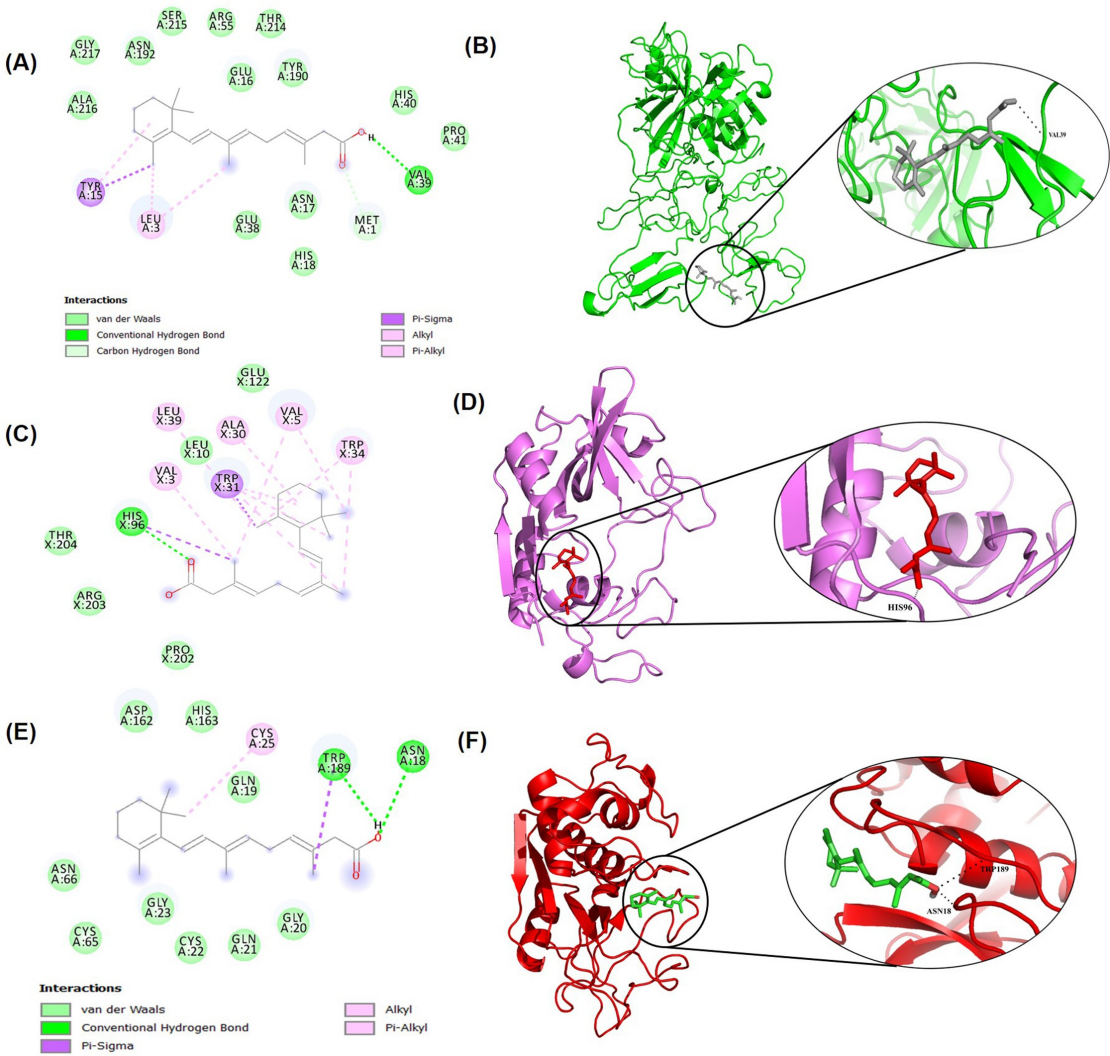
Molecular docking study of retinoic acid to TMPRSS2, CTSB, and CTSL. 2D diagrams of (A) TMPRSS2- retinoic acid, (C) CTSB-retinoic acid, and (E) CTSL-retinoic acid interactions from Ligplot+ analysis. The figure provides the information about the interactions and the protein residues and interactions are colored accordingly. The best docking poses in the 3D structure of the proteins are represented in (B), (D), and (F).

Molecular docking studies have largely been employed to know the binding modes of ligand and receptor and are generally used in drug discovery. Through the docking, we here assessed the interaction of estradiol and retinoic acid to TMPRSS2 and CTSB/L. The proposed binding mode is presented in **Figs 5 and 6**, and the docking results based on the binding affinity have been represented in **Table 1**.

**Table 1:** Molecular docking results of estradiol and retinoic acid to target TMPRSS2 and CTSB/L.

Protein-Drug	Binding energy (kcal/mol)	H-bond	Vander Waal bond	Other bonds
TMPRSS2-Estradiol	-7.4	ASN177, TYR180	VAL171, PRO170, HIS169, ASP134, TRP168, ASN131, GLN173, ASN193, ALA235, LYS234, SER233, TRP176, ASP175	TRP132, CYS172
CTSL-Estradiol	-6.8	GLY20, GLY194	GLN21, ASN18, TRP193, GLU192, LYS147, TYR146, PHE145	TRP189, LEU144
CTSB-Estradiol	-7.4	VAL5	GLY43, GLU4, GLY42, SER6, PRO57, ILE56, CYS70, CYS59, PRO58, SER171	ALA7, TYR54, LEU44, HIS61
TMPRSS2-Retinoic	-6.4	VAL39	ALA216, GLY217, ASN192, SER215, SRG55, THR214, GLU16, TYR190, HIS40, PRO41, ASN17, GLU38, HIS18	MET1, LEU3, TYR15
CTSB-Retinoic	-7.9	HIS96	LEU10, GLU122, PRO202, ARG203, THR204,	VAL3, VAL5, ALA30, TRP31, TRP34, LEU39
CTSL-Retinoic	-6.0	ASN18, TRP189	ASP162, HIS163, GLN19, ASN66, CYS65, GLY23, CYS22, GLN21, GLY20	CYS25

The interaction analysis of estradiol with TMPRSS2 revealed the formation of two hydrogen (H) bonds at ASN177 and TYR180 positions along with thirteen Van der Waal bonds and one pi-sulfur and alkyl bond each (**Fig 5A and 5B**). The interaction of estradiol with CTSL revealed the formation of two H-bonds at the position, GLY20 and GLY194 along with seven Van der Waal bonds and two alkyl bonds (**Fig 5C and 5D**). Interaction study of estradiol with CTSB showed one H-bond at VAL5 position along with nine Van der Waal bonds, one carbon-hydrogen bond, and three other bonds (**Fig 5E and 5F**).

Retinoic acid binds with CTSB, CTSL, and TMPRSS2 with the binding affinity of -7.9 kcal/mol, -6.0 kcal/mol, and -6.4 kcal/mol, respectively. Retinoic acid form one H-bond to VAL39 with TMPRSS2 along with thirteen Van der Waal bonds, one carbon-hydrogen bond, and one alkyl and pi-sigma bond each (**Fig 6A and 6B**). Similarly, retinoic acid form two H-bonds at position TRP189 and ASN18 with CTSL along with nine Van der Waal bonds and one alkyl bond (**Fig 6C and 6D**). Interaction study of retinoic acid with CTSB showed one H- bond at position HIS96 along with five Van der Waal interactions, one pi-sigma bond, and five alkyl bonds (**Fig 6E and 6F**). The interaction study represents how stringently is the binding of estradiol and retinoic acid with the target proteins ultimately helps to lock the ligand molecules in the binding pocket and thus effectively inhibits the target proteins.

## 4. Discussion

The potential modulators of TMPRSS2 and CTSB/L were investigated through GSEA analysis and the potential drugs regulating these modulators were identified and examined in this study. Peroxisome proliferator-activated receptors (PPARs) belong to the ligand-activated nuclear hormone receptors (NR) superfamily and recently emerged as key players of inflammation. The PPAR family has PPAR-α, PPAR-β/δ, and PPAR-γ that are encoded by distinct genes. PPAR-γ expression is repressed in inflammatory lungs of patients with severe COVID-19. The repression of PPAR-γ plays a key role in the induction of cytokine storm of inflammatory monocytes/macrophages in the SARS-CoV-2-infected lung. It has also been shown that SARS-CoV-2 modifies lipid metabolism in the lung epithelial cells by modulating the expression of PPARα, and thus contributes to lipotoxicity and respiratory problems [30]. Thus, downregulation of PPARs in COVID-19 may be considered as an important modulator of pulmonary inflammation and acute lung injury [31]. In this regard, the activation of PPARs may serve as an effective therapeutic strategy to decrease the inflammatory perturbations during SARS-CoV-2 infection. Recently, Ehrlich et al reported that the PPARα agonist, fenofibrate decreased the phospholipid accumulation in SARS-CoV-2 infected cells, and inhibited viral replication [30]. Fenofibrate inhibits the downregulation of PPARα activation caused by inflammation, decreases cytokine production by LPS or TNFα [32, 33], and improves fatty acid oxidation, thus averting acute lung injury [31]. Moreover, the activation of PPARs requires the heterodimerization with another nuclear receptor, the retinoid X receptor (RXR) [34], that are activated by endogenous 9-cis retinoic acid [18], indicating a protective role of retinoic acid in lung injury.

Retinoic acid has been involved in the regulation of transcription of over 500 genes [35]. The pulmonary, immunomodulatory, and antimicrobial functions of retinoic acid play a crucial role in reducing viral diseases, including COVID-19 infection [36]. It has been involved in modifying the pathogenesis of acute respiratory distress syndrome (ARDS), regulating the production of IL1-β, and IL-1 receptor antagonists and the subsequent pulmonary access of neutrophils [37]. In addition, the combination of retinoic acid with simvastatin shown to be involved in pulmonary regeneration and remodeling in animal studies [38]. Retinoic acid also plays a crucial role in viral infections because of its involvement in the development of innate immunity against RNA virus through type-I interferon-facilitated mechanism (retinoic acid-inducible gene I, RIG-1), which helps in the protection of bystander immune cells against a subsequent round of viral replication [39].

Additionally, TMPRSS2 is sensitive to Dihydrotestosterone (DHT), and its expression is increased in a dosage-dependent manner. Retinoic acid has been found to inhibit DHT, thus resulting in an inhibition of androgen receptor stimulation, and downregulating the expression of TMPRSS2, and thus reduce SARS-CoV-2 infection. In light of this, a randomized, phase II, placebo-controlled clinical trial (ClinicalTrials.gov; Identifier: NCT04578236) for assessing the efficacy of aerosol combination therapy of 13- cis retinoic acid and captopril for treating COVID-19 patients via indirect inhibition of TMPRSS2 is currently ongoing.

The susceptibility to SARS-CoV-2 infection is almost similar in both genders, but males have higher severity and mortality. It has been noted that TMPRSS2 is an androgen-dependent protein, signifying that SARS-CoV-2 infection is probably androgen-mediated [40]. Estradiol has been shown to be protective against multiple pathological complications ranging from ARDS, inflammation, autoimmune diseases, viral infections to neurological disorders. Because of such wide-ranging protective effects, estradiol might control SARS-CoV-2 infection by affecting the renin-angiotensin-aldosterone system (RAAS), suppressing inflammatory storms, inducing anti-viral immune responses, and enhancing the virus degradation through upregulation of endolysosomal degradation pathways [41, 42]. In line with this, a clinical trial testing the effect of sex hormones (estrogen and testosterone) on COVID-19 outcomes (ClinicalTrials.gov; Identifier: NCT04359329).

Interestingly, a hypothetical bipartite combination consisting of estradiol and retinoic acid may alter the expression of 461 of 809 (56%) human genes encoding SARS-CoV-2 targets and interfere with the functions of all SARS-CoV-2 viral proteins. Remarkably, estradiol and retinoic acid significantly modulates the PPI network of human and SARS-CoV-2 proteins and thus could manifest better therapeutic benefits by targeting a large number of genes involved in SARS-CoV-2 infection.

## 5. Conclusions

This study aimed to identify human genes involved in the regulation of the expression and functions of the SARS-CoV-2 entry genes, TMPRSS2 and CTSB/L. These identified genes may act as activators and /or repressors of TMPRSS2 and/or CTSB/L and provide necessary information to understand the regulatory interactions observed during COVID-19 infection. A panel of existing drugs and ligands against these regulatory genes were then identified that could be considered for drug-repurposing to mitigate the outcomes of COVID-19. Two of the most promising candidate drugs, namely estradiol and retinoic acid modulates the gene expression of TMPRSS2 and CTSB/L and their modifiers. Our findings are in excellent agreement with recent studies reporting the significant COVID-19 mitigation potential of estradiol and retinoic acid [42–44]. Interestingly, a hypothetical bipartite combination (estradiol and retinoic acid) indicated strong effects on the interactome of SARS-CoV-2 target human host genes compared to monotherapies.

## Supporting information

S1 File Supplementary figures contains all the supporting figures (S1-S16 Figs in S1 File). (PDF)

S1 Table Involvement of TMPRSS2, CTSB, and CTSL genes in COVID-19 related gene sets obtained from Enrichr. (XLSX)

S2 Table Involvement of putative modulators of target genes in COVID-19 related gene sets obtained from Enrichr. (XLSX)
