# Supplementary material for: Genomics-guided identification of potential modulators of SARS-CoV-2 entry proteases, TMPRSS2 and Cathepsins B/L
Source: PLoS One. 2021 Aug 18;16(8):e0256141. doi: 10.1371/journal.pone.0256141 (PMC8372896; doi:10.1371/journal.pone.0256141)
Supplement: S1 File — (PDF) [file pone.0256141.s001.pdf]

Supplementary Figure 1. RNA-Seq expression data of (A) TMPRSS2, (B) CTSB, and (C) CTSL genes from GTEx web portal.

(A)

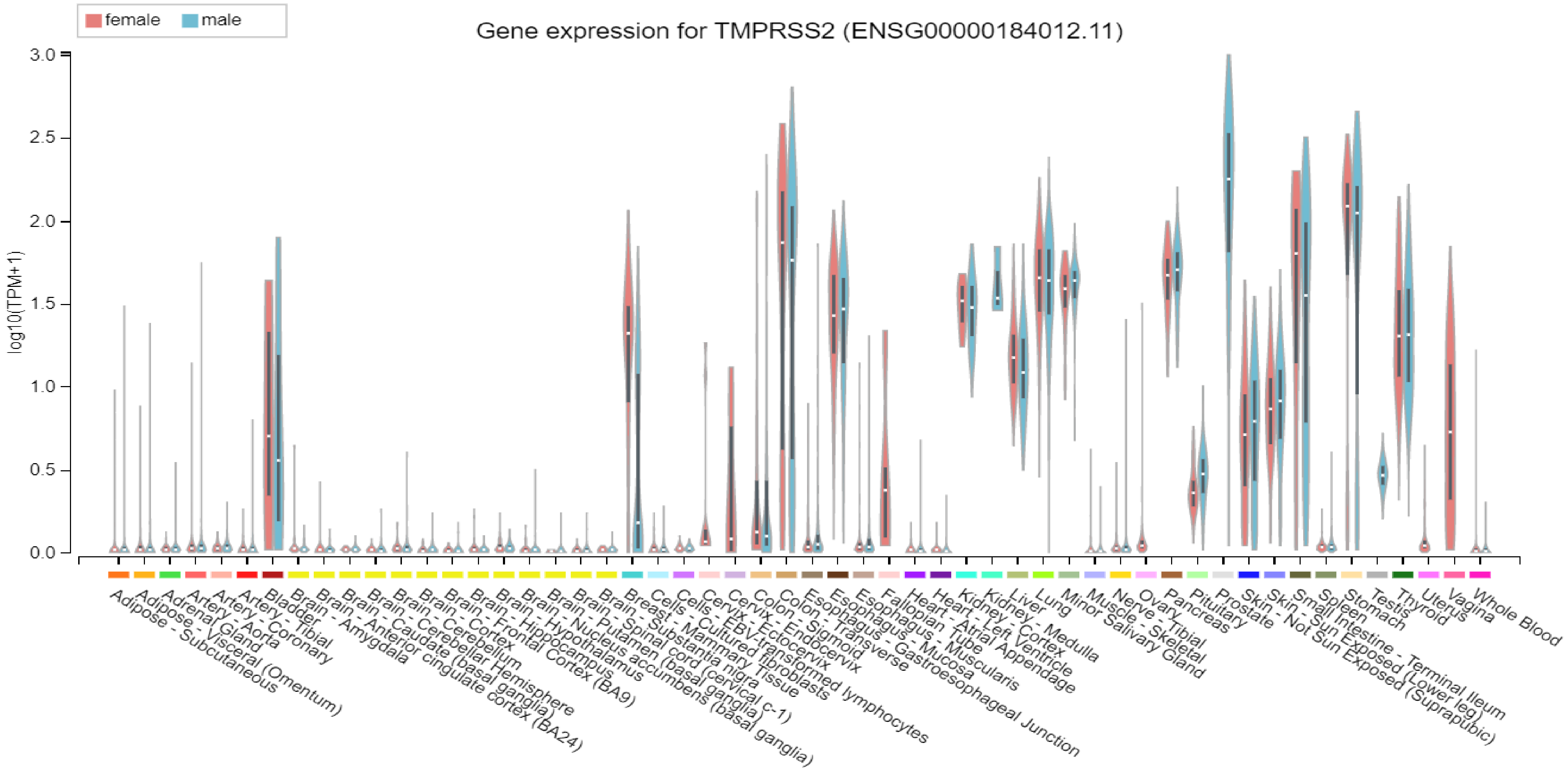
female
male
Gene expression for TMPRSS2 (ENSG00000184012.11)
log10(TPM+1)
3.0
2.5
2.0
1.5
1.0
0.5
0.0
Adipose - Subcutaneous
Adipose - Visceral (Omentum)
Adrenal Gland
Artery - Aorta
Artery - Coronary
Artery - Tibial
Bladder
Brain - Amygdala
Brain - Anterior cingulate cortex (BA24)
Brain - Caudate (basal ganglia)
Brain - Cerebellar Hemisphere
Brain - Cerebellum
Brain - Cortex
Brain - Frontal Cortex (BA9)
Brain - Hippocampus
Brain - Hypothalamus
Brain - Nucleus accumbens (basal ganglia)
Brain - Putamen (basal ganglia)
Brain - Spinal cord (cervical c-1)
Brain - Substantia nigra
Breast - Mammary Tissue
Cells - Cultured fibroblasts
Cells - EBV-transformed lymphocytes
Cervix - Ectocervix
Cervix - Endocervix
Colon - Sigmoid
Colon - Transverse
Esophagus - Gastroesophageal Junction
Esophagus - Mucosa
Esophagus - Muscularis
Fallopian Tube
Heart - Atrial Appendage
Heart - Left Ventricle
Kidney - Cortex
Kidney - Medulla
Liver
Lung
Minor Salivary Gland
Muscle - Skeletal
Nerve - Tibial
Ovary
Pancreas
Pituitary
Prostate
Skin - Not Sun Exposed (Suprapubic)
Skin - Sun Exposed (Lower leg)
Small Intestine - Terminal Ileum
Spleen
Stomach
Testis
Thyroid
Uterus
Vagina
Whole Blood

(B)

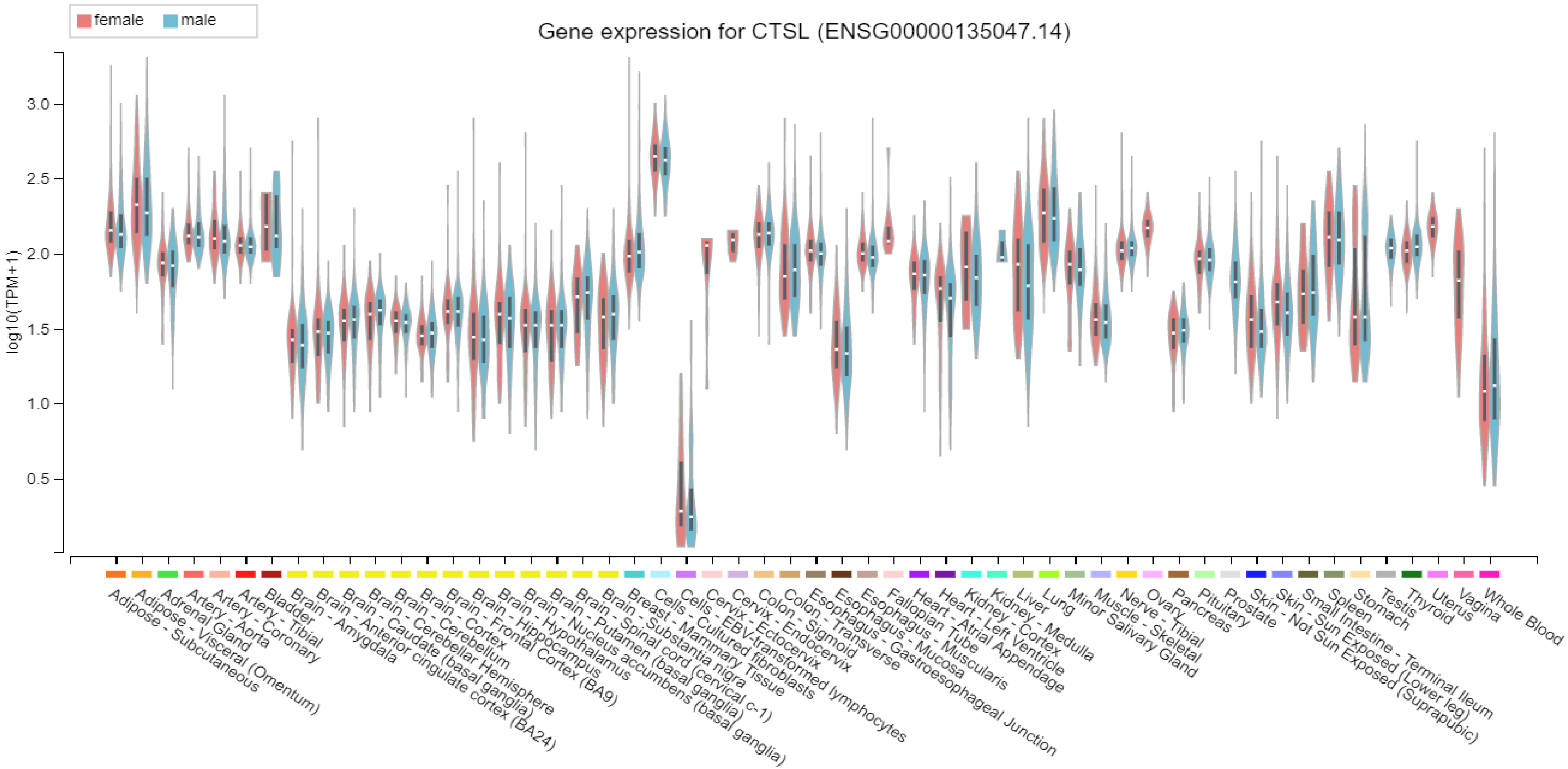
female
male
Gene expression for CTSL (ENSG00000135047.14)
log10(TPM+1)
3.0
2.5
2.0
1.5
1.0
0.5
Adipose - Subcutaneous
Adipose - Visceral (Omentum)
Adrenal Gland
Artery - Aorta
Artery - Coronary
Artery - Tibial
Bladder
Brain - Amygdala
Brain - Anterior cingulate cortex (BA24)
Brain - Caudate (basal ganglia)
Brain - Cerebellar Hemisphere
Brain - Cerebellum
Brain - Cortex
Brain - Frontal Cortex (BA9)
Brain - Hippocampus
Brain - Hypothalamus
Brain - Nucleus accumbens (basal ganglia)
Brain - Putamen (basal ganglia)
Brain - Spinal cord (cervical c-1)
Brain - Substantia nigra
Breast - Mammary Tissue
Cells - Cultured fibroblasts
Cells - EBV-transformed lymphocytes
Cervix - Ectocervix
Cervix - Endocervix
Colon - Sigmoid
Colon - Transverse
Esophagus - Gastroesophageal Junction
Esophagus - Mucosa
Esophagus - Muscularis
Fallopian Tube
Heart - Atrial Appendage
Heart - Left Ventricle
Kidney - Cortex
Kidney - Medulla
Liver
Lung
Minor Salivary Gland
Muscle - Skeletal
Nerve - Tibial
Ovary
Pancreas
Pituitary
Prostate
Skin - Not Sun Exposed (Suprapubic)
Skin - Sun Exposed (Lower leg)
Small Intestine - Terminal Ileum
Spleen
Stomach
Testis
Thyroid
Uterus
Vagina
Whole Blood

(c)

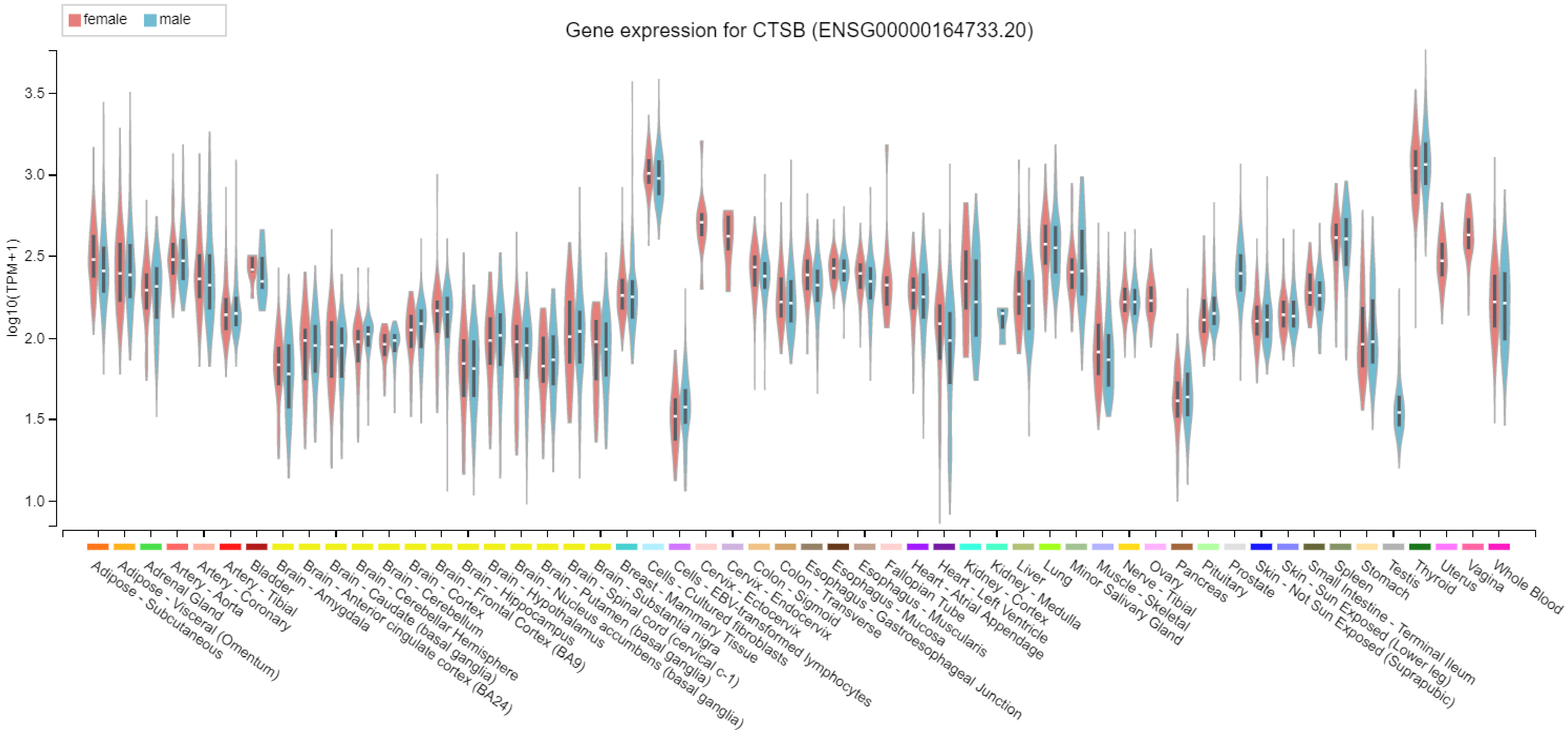
female
male
Gene expression for CTSB (ENSG00000164733.20)
log10(TPM+1)
3.5
3.0
2.5
2.0
1.5
1.0
Adipose - Subcutaneous
Adipose - Visceral (Omentum)
Adrenal Gland
Artery - Aorta
Artery - Coronary
Artery - Tibial
Bladder
Brain - Amygdala
Brain - Anterior cingulate cortex (BA24)
Brain - Caudate (basal ganglia)
Brain - Cerebellar Hemisphere
Brain - Cerebellum
Brain - Cortex
Brain - Frontal Cortex (BA9)
Brain - Hippocampus
Brain - Hypothalamus
Brain - Nucleus accumbens (basal ganglia)
Brain - Putamen (basal ganglia)
Brain - Spinal cord (cervical c-1)
Brain - Substantia nigra
Breast - Mammary Tissue
Cells - Cultured fibroblasts
Cells - EBV-transformed lymphocytes
Cervix - Ectocervix
Cervix - Endocervix
Colon - Sigmoid
Colon - Transverse
Esophagus - Gastroesophageal Junction
Esophagus - Mucosa
Esophagus - Muscularis
Fallopian Tube
Heart - Atrial Appendage
Heart - Left Ventricle
Kidney - Cortex
Kidney - Medulla
Liver
Lung
Minor Salivary Gland
Muscle - Skeletal
Nerve - Tibial
Ovary
Pancreas
Pituitary
Prostate
Skin - Not Sun Exposed (Suprapubic)
Skin - Sun Exposed (Lower leg)
Small Intestine - Terminal Ileum
Spleen
Stomach
Testis
Thyroid
Uterus
Vagina
Whole Blood

Supplementary Figure 2. COVID-19 related gene sets from Enrichr web server

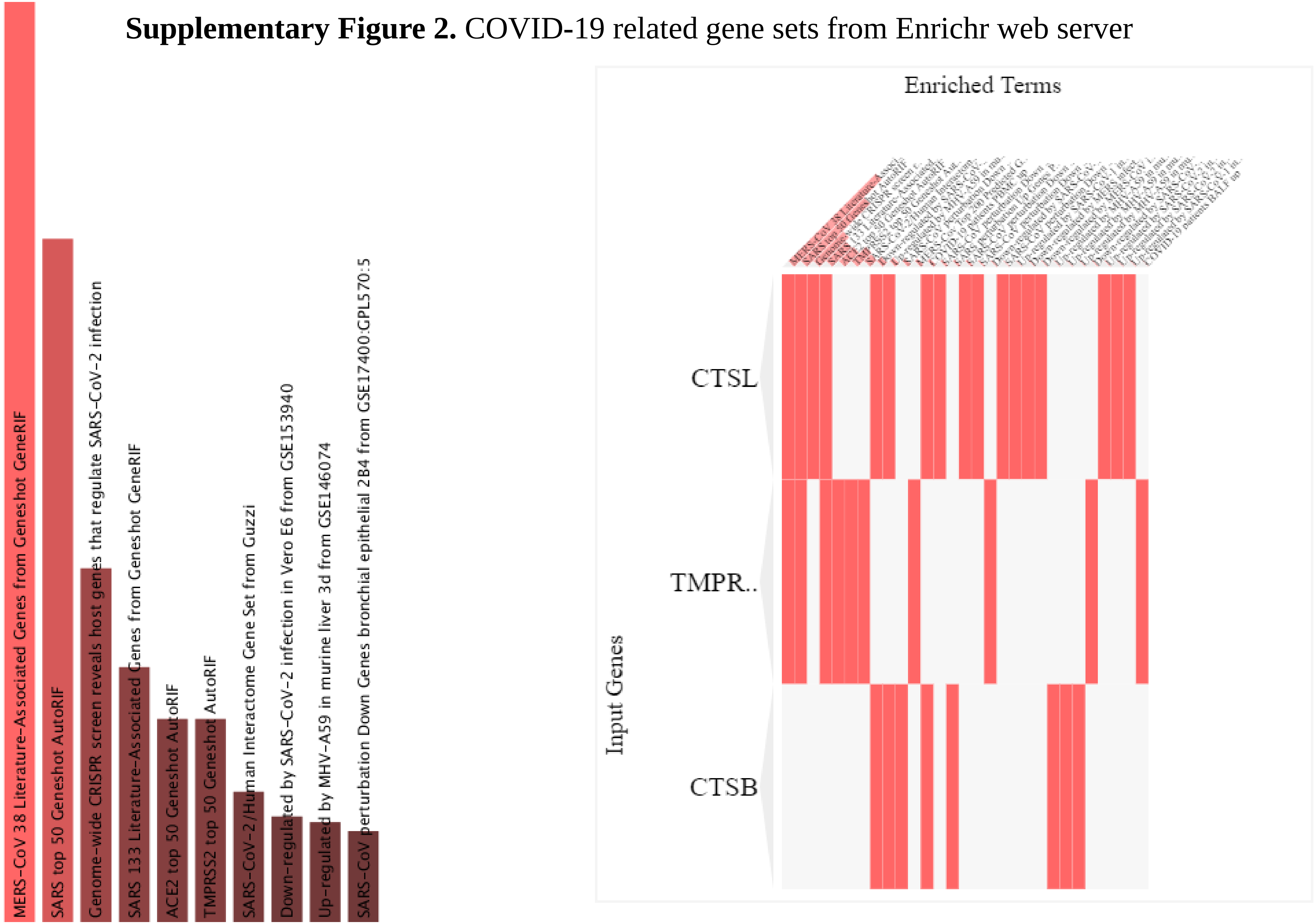

Supplementary Figure 3. Effects of viral perturbations on **e**xpression profile data from NCBI GEO database.

(A) TMPRSS2 - Severe acute respiratory syndrome expression profile

**Profile**	GDS1028 / 205102_at
**Title**	Severe acute respiratory syndrome expression profile
**Organism**	Homo sapiens

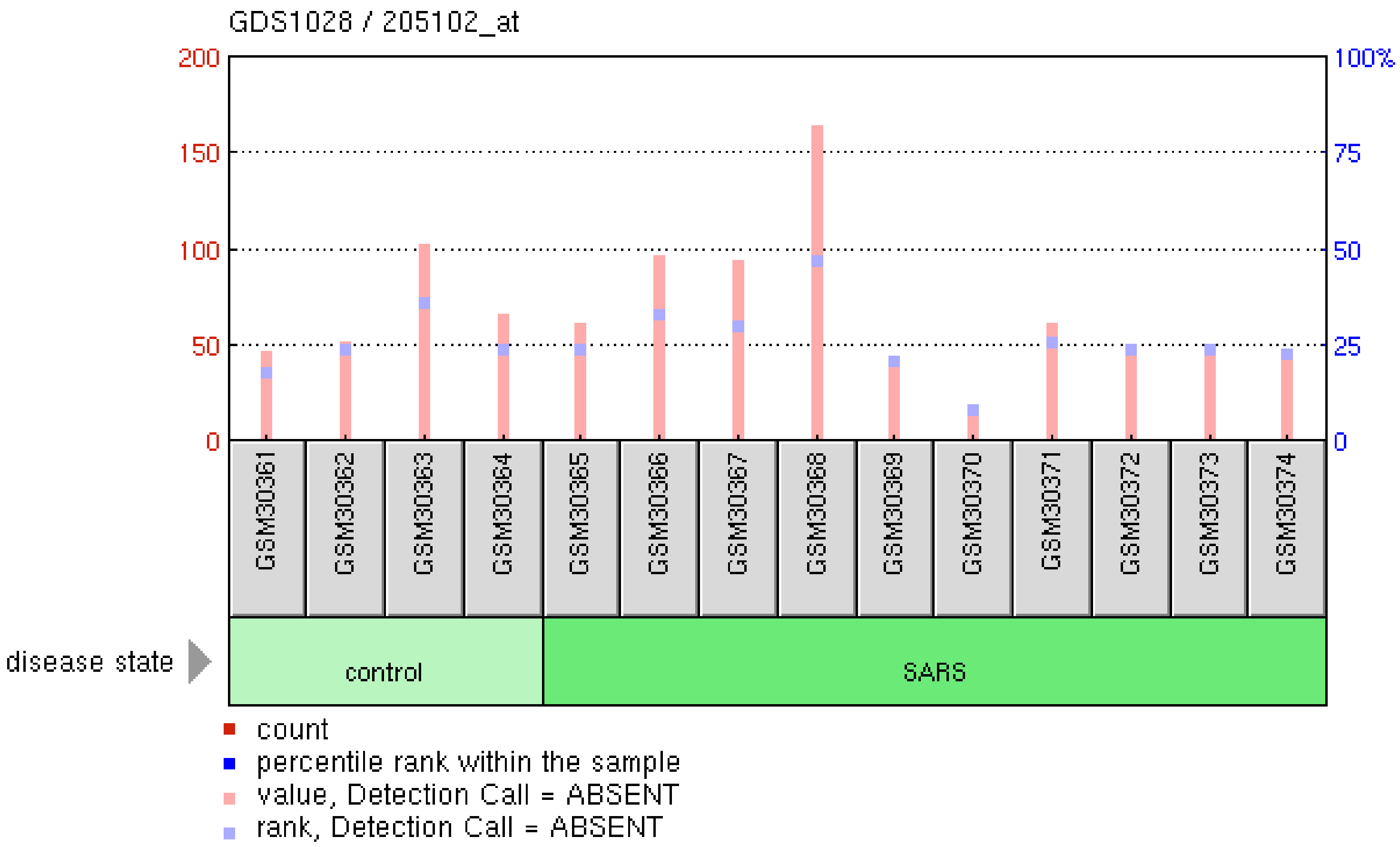

Sample	Title	Value
GSM30361	N1	47.2
GSM30362	N2	51.9
GSM30363	N3	103.1
GSM30364	N4	66.4
GSM30365	S1	61.5
GSM30366	S2	97.1
GSM30367	S3	94.1
GSM30368	S4	164.9
GSM30369	S5	45.4
GSM30370	S6	17.1
GSM30371	S7	62
GSM30372	S8	50.9
GSM30373	S9	49.2
GSM30374	S10	42.5

(B) CTSB - Severe acute respiratory syndrome expression profile

**Profile**	GDS1028 / 200839_s_at
**Title**	Severe acute respiratory syndrome expression profile
**Organism**	Homo sapiens

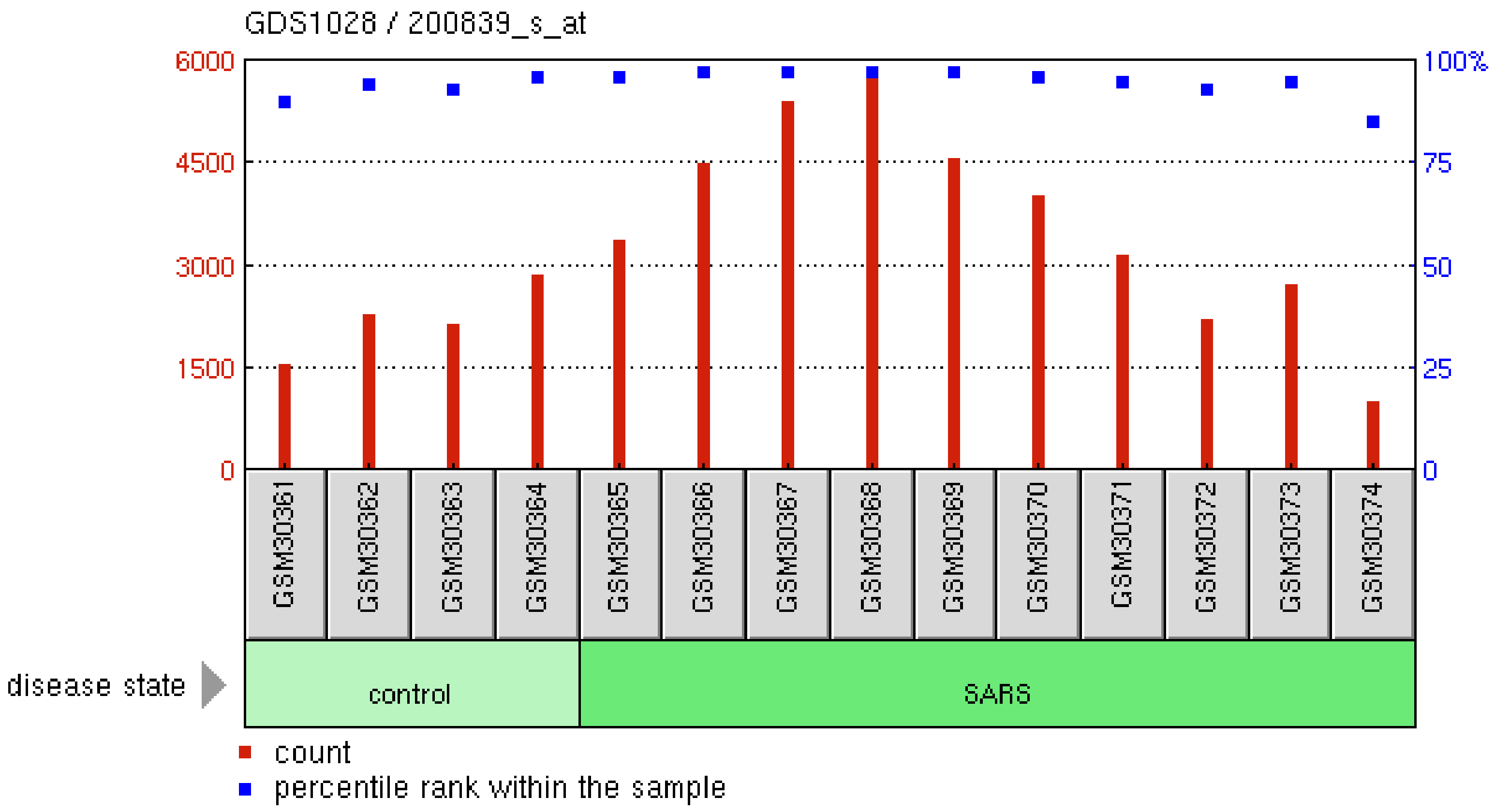

Sample	Title	Value
GSM30361	N1	1556.1
GSM30362	N2	2304
GSM30363	N3	2140.2
GSM30364	N4	2867
GSM30365	S1	3397.5
GSM30366	S2	4517.3
GSM30367	S3	5418.1
GSM30368	S4	5797.7
GSM30369	S5	4568.4
GSM30370	S6	4016.1
GSM30371	S7	3174.4
GSM30372	S8	2216.5
GSM30373	S9	2743
GSM30374	S10	1020.6

(C) CTSL - Severe acute respiratory syndrome expression profile

**Profile**	GDS1028 / 202087_s_at
**Title**	Severe acute respiratory syndrome expression profile
**Organism**	Homo sapiens

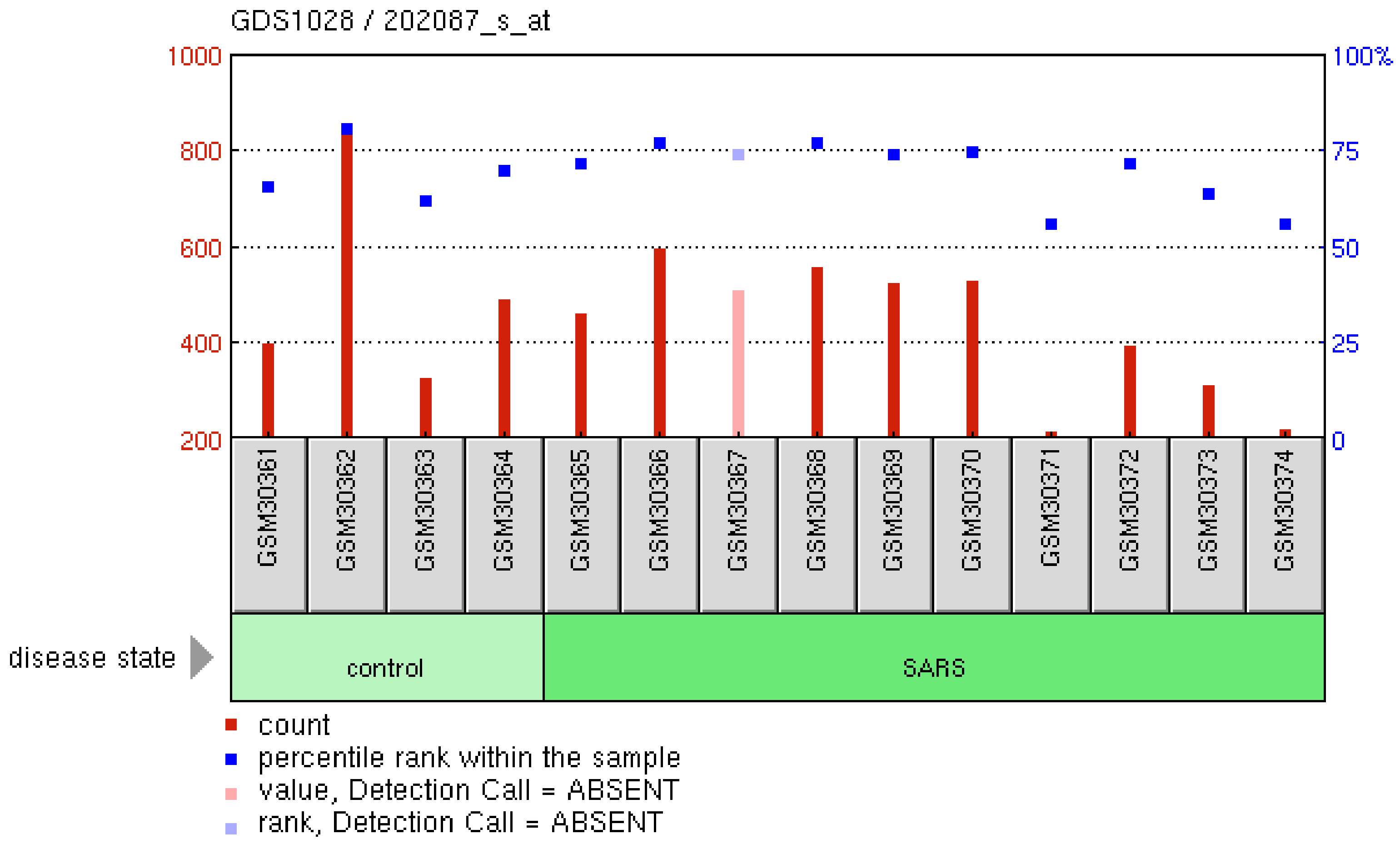

Sample	Title	Value
GSM30361	N1	398.6
GSM30362	N2	832
GSM30363	N3	326.3
GSM30364	N4	489.4
GSM30365	S1	460.7
GSM30366	S2	596
GSM30367	S3	511.3
GSM30368	S4	561.1
GSM30369	S5	524.8
GSM30370	S6	532.4
GSM30371	S7	215.4
GSM30372	S8	393.8
GSM30373	S9	315.4
GSM30374	S10	205.2

Supplementary Figure 4. Effects of human diseases on expression changes. Expression changes of TMPRSS2 and CTSB/L genes leads to very different diseases patterns.

Disease Perturbations from GEO up

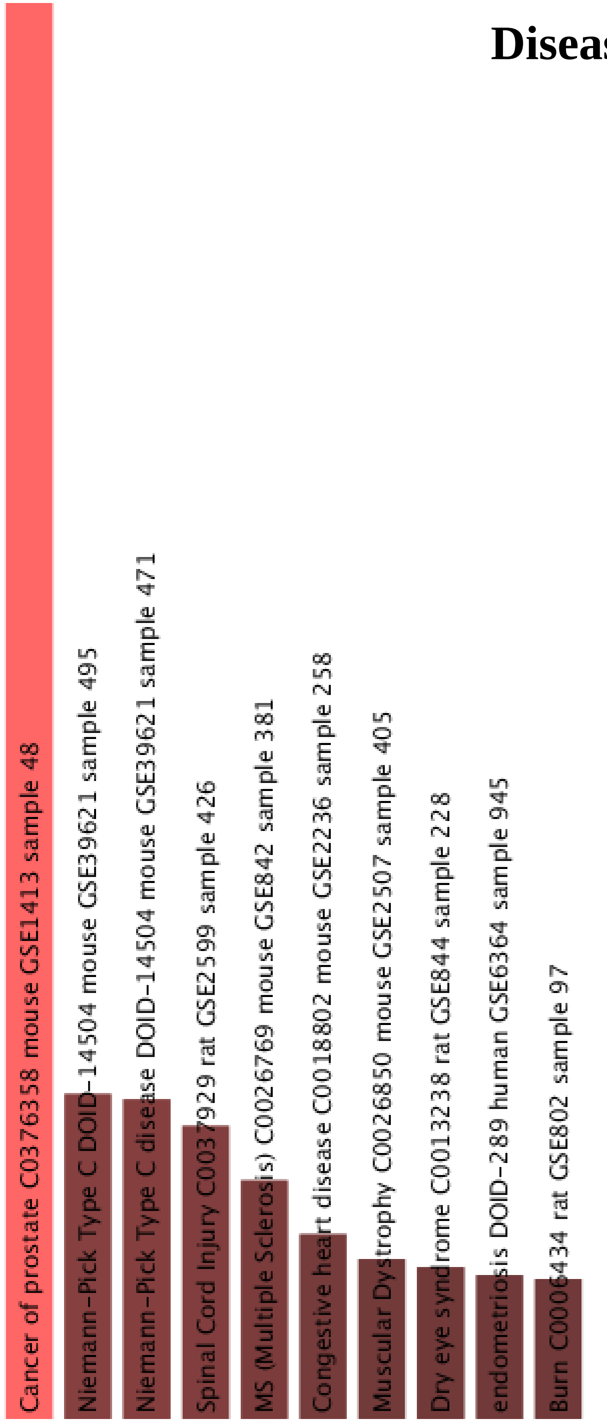

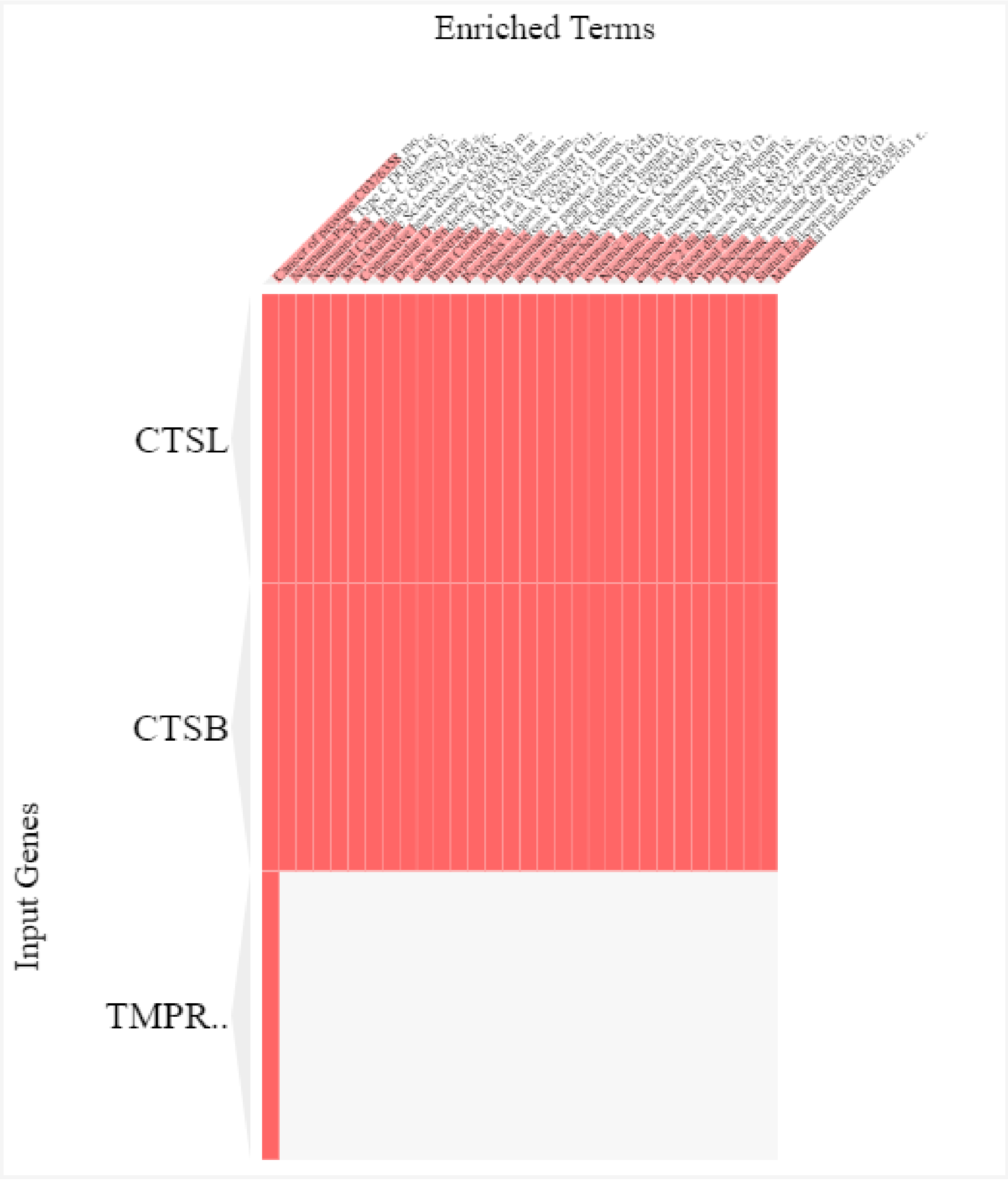

DisGeNET

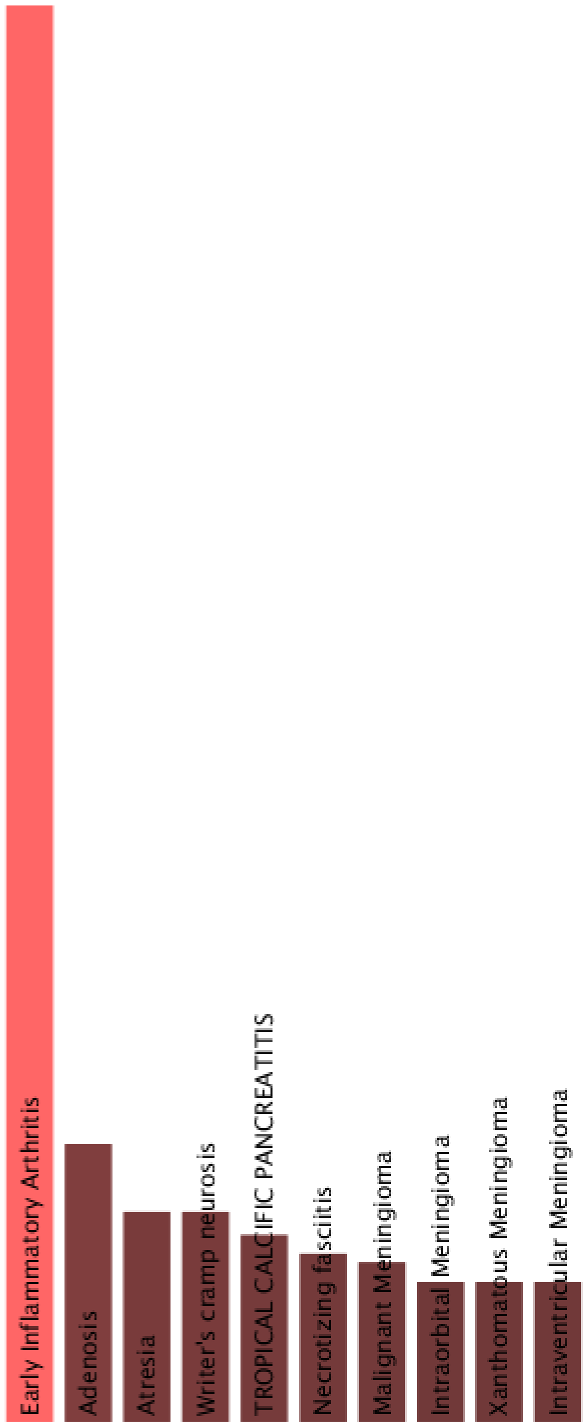

Early Inflammatory Arthritis
Adenosis
Atresia
Writer's cramp neurosis
TROPICAL CALCIFIC PANCREATITIS
Necrotizing fasciitis
Malignant Meningioma
Intraorbital Meningioma
Xanthomatous Meningioma
Intraventricular Meningioma

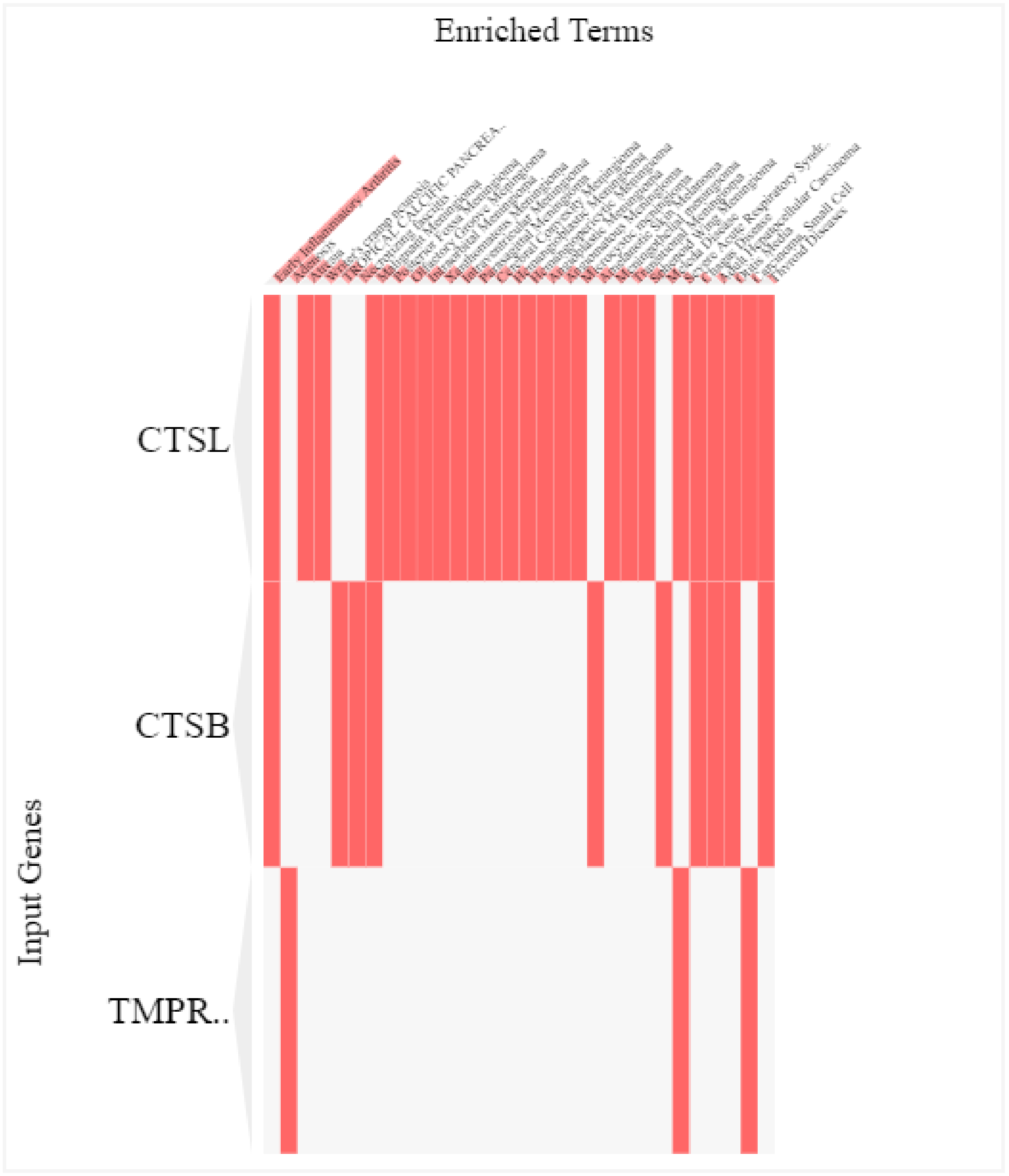

Enriched Terms
Input Genes
CTSL
CTSB
TMPR..

TMPRSS2 - Pandemic and seasonal H1N1 influenza virus infections of bronchial epithelial cells in vitro

**Profile**	GDS4855 / 205102_at
**Title**	Pandemic and seasonal H1N1 influenza virus infections of bronchial epithelial cells in vitro
**Organism**	Homo sapiens

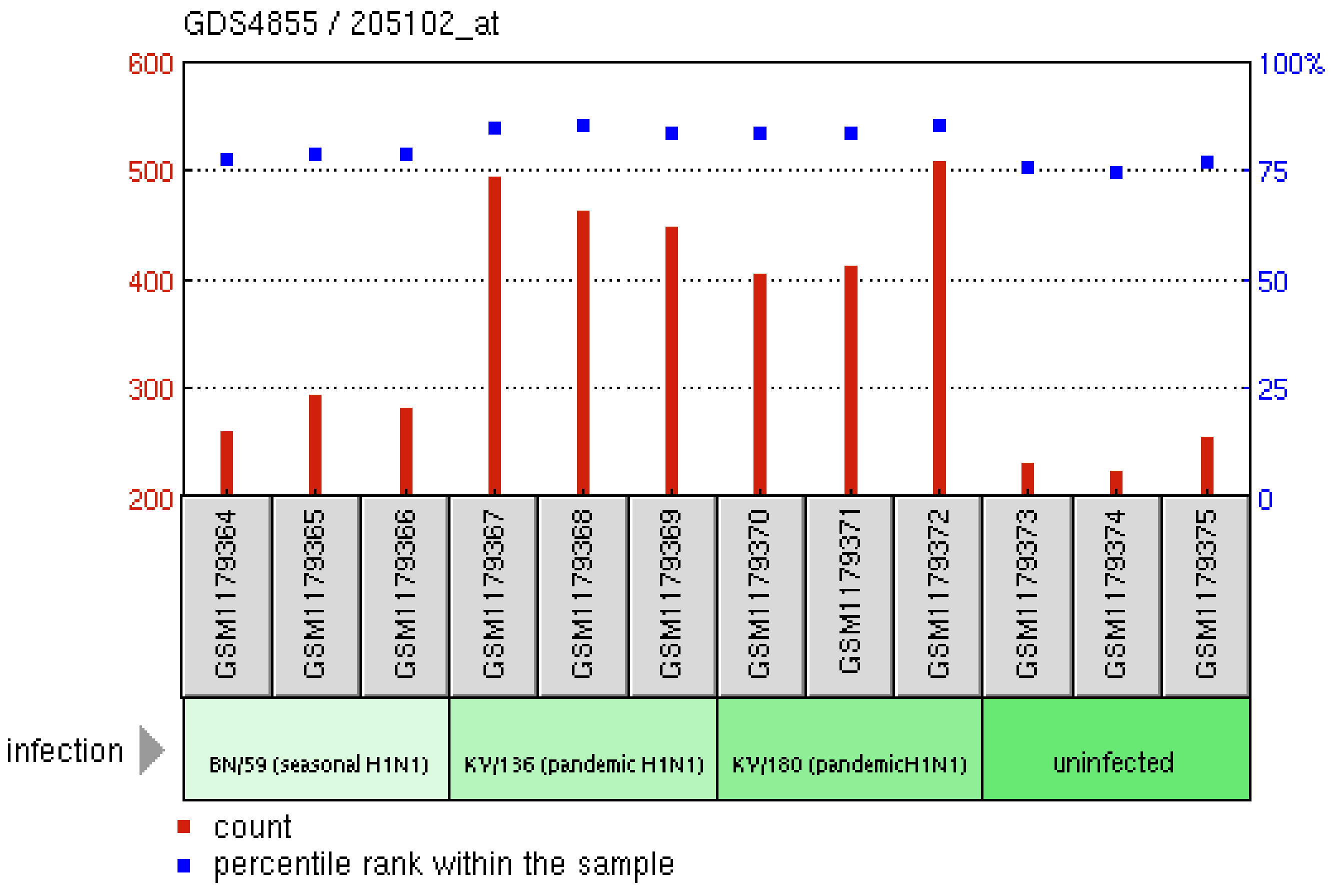

Sample	Title	Value
GSM1179364	wdNHBE with BN/59 at 36hpi, biological rep1	261.058
GSM1179365	wdNHBE with BN/59 at 36hpi, biological rep2	296.302
GSM1179366	wdNHBE with BN/59 at 36hpi, biological rep3	282.786
GSM1179367	wdNHBE with KY/136 at 36hpi, biological rep1	495.862
GSM1179368	wdNHBE with KY/136 at 36hpi, biological rep2	463.829
GSM1179369	wdNHBE with KY/136 at 36hpi, biological rep3	449.964
GSM1179370	wdNHBE with KY/180 at 36hpi, biological rep1	405.291
GSM1179371	wdNHBE with KY/180 at 36hpi, biological rep2	413.315
GSM1179372	wdNHBE with KY/180 at 36hpi, biological rep3	510.487
GSM1179373	wdNHBE uninfected at 36hpi, biological rep1	232.881
GSM1179374	wdNHBE uninfected at 36hpi, biological rep2	226.012
GSM1179375	wdNHBE uninfected at 36hpi, biological rep3	256.634

CTSB - Pandemic and seasonal H1N1 influenza virus infections of bronchial epithelial cells in vitro

**Profile**	GDS4855 / 213274_s_at
**Title**	Pandemic and seasonal H1N1 influenza virus infections of bronchial epithelial cells in vitro
**Organism**	Homo sapiens

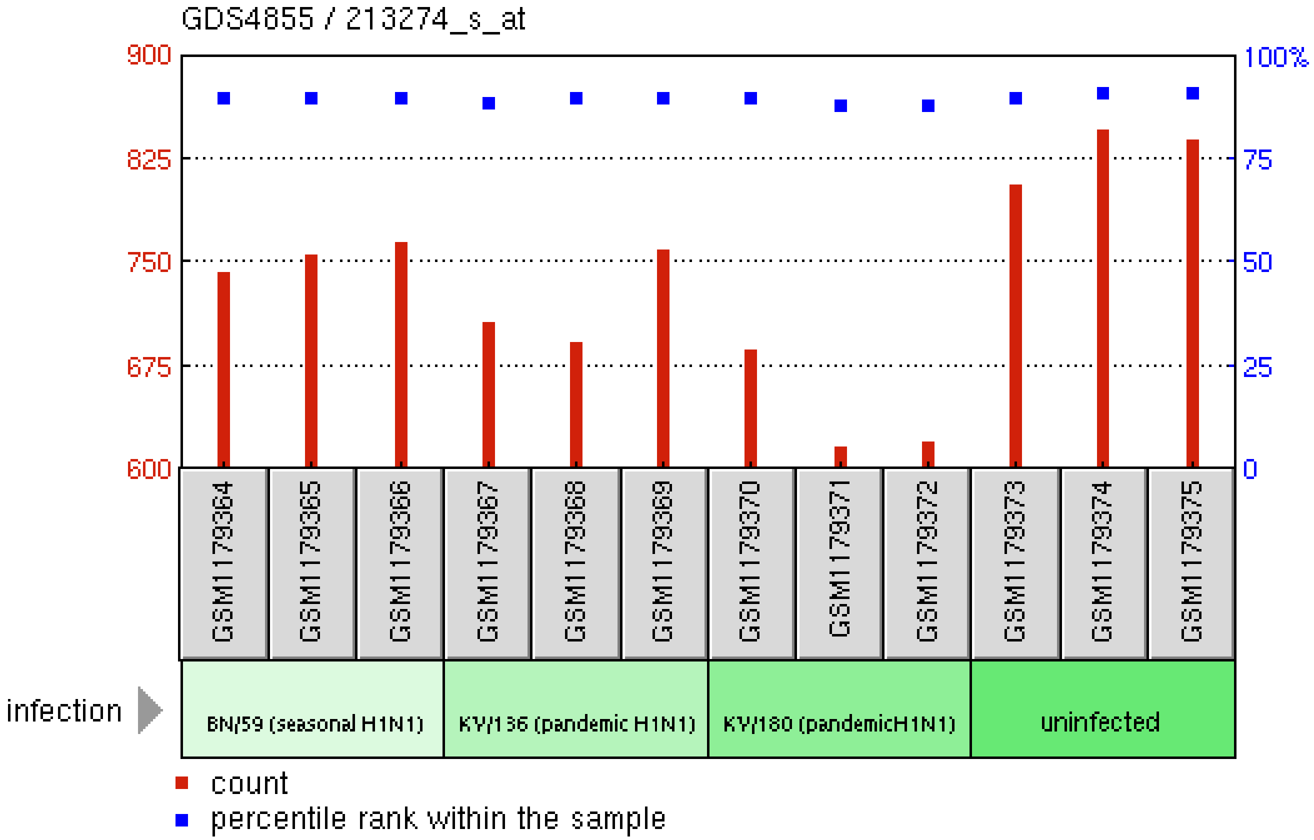

Sample	Title	Value
GSM1179364	wdNHBE with BN/59 at 36hpi, biological rep1	744.477
GSM1179365	wdNHBE with BN/59 at 36hpi, biological rep2	757.03
GSM1179366	wdNHBE with BN/59 at 36hpi, biological rep3	766.004
GSM1179367	wdNHBE with KY/136 at 36hpi, biological rep1	707.854
GSM1179368	wdNHBE with KY/136 at 36hpi, biological rep2	692.712
GSM1179369	wdNHBE with KY/136 at 36hpi, biological rep3	759.545
GSM1179370	wdNHBE with KY/180 at 36hpi, biological rep1	687.437
GSM1179371	wdNHBE with KY/180 at 36hpi, biological rep2	617.024
GSM1179372	wdNHBE with KY/180 at 36hpi, biological rep3	620.183
GSM1179373	wdNHBE uninfected at 36hpi, biological rep1	807.724
GSM1179374	wdNHBE uninfected at 36hpi, biological rep2	846.055
GSM1179375	wdNHBE uninfected at 36hpi, biological rep3	840.318

CTSB - Pandemic and seasonal H1N1 influenza virus infections of bronchial epithelial cells in vitro

**Profile**	GDS4855 / 200839_s_at
**Title**	Pandemic and seasonal H1N1 influenza virus infections of bronchial epithelial cells in vitro
**Organism**	Homo sapiens

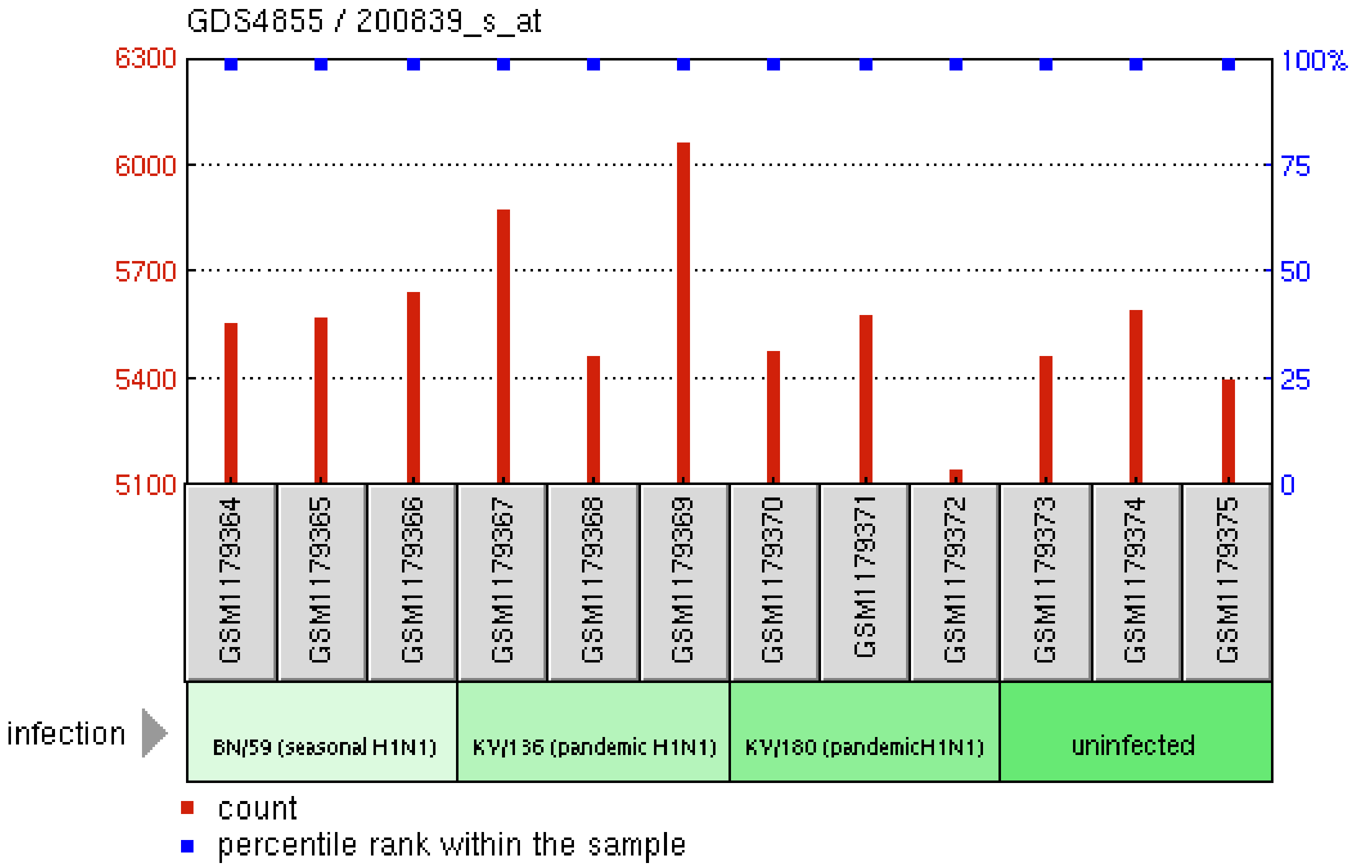

Sample	Title	Value
GSM1179364	wdNHBE with BN/59 at 36hpi, biological rep1	5560.64
GSM1179365	wdNHBE with BN/59 at 36hpi, biological rep2	5574.77
GSM1179366	wdNHBE with BN/59 at 36hpi, biological rep3	5648.88
GSM1179367	wdNHBE with KY/136 at 36hpi, biological rep1	5880.23
GSM1179368	wdNHBE with KY/136 at 36hpi, biological rep2	5463.98
GSM1179369	wdNHBE with KY/136 at 36hpi, biological rep3	6061.65
GSM1179370	wdNHBE with KY/180 at 36hpi, biological rep1	5482.89
GSM1179371	wdNHBE with KY/180 at 36hpi, biological rep2	5582.37
GSM1179372	wdNHBE with KY/180 at 36hpi, biological rep3	5150.42
GSM1179373	wdNHBE uninfected at 36hpi, biological rep1	5461.47
GSM1179374	wdNHBE uninfected at 36hpi, biological rep2	5597.5
GSM1179375	wdNHBE uninfected at 36hpi, biological rep3	5402.46

CTSL - Pandemic and seasonal H1N1 influenza virus infections of bronchial epithelial cells in vitro

**Profile**	GDS4855 / 202087_s_at
**Title**	Pandemic and seasonal H1N1 influenza virus infections of bronchial epithelial cells in vitro
**Organism**	Homo sapiens

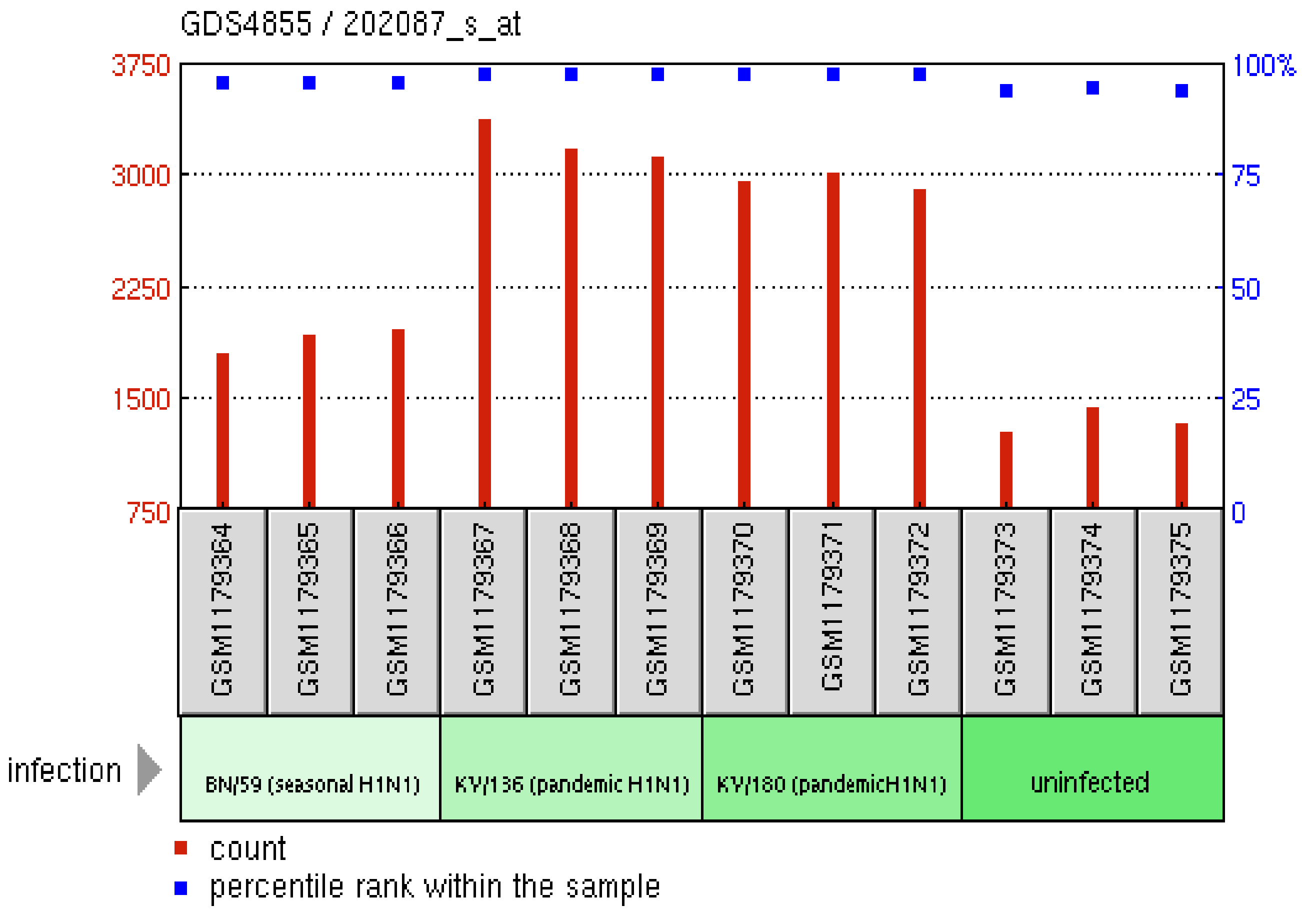

Sample	Title	Value
GSM1179364	wdNHBE with BN/59 at 36hpi, biological rep1	1798.26
GSM1179365	wdNHBE with BN/59 at 36hpi, biological rep2	1939.88
GSM1179366	wdNHBE with BN/59 at 36hpi, biological rep3	1965.45
GSM1179367	wdNHBE with KY/136 at 36hpi, biological rep1	3385.59
GSM1179368	wdNHBE with KY/136 at 36hpi, biological rep2	3180.91
GSM1179369	wdNHBE with KY/136 at 36hpi, biological rep3	3123.02
GSM1179370	wdNHBE with KY/180 at 36hpi, biological rep1	2972.69
GSM1179371	wdNHBE with KY/180 at 36hpi, biological rep2	3025.29
GSM1179372	wdNHBE with KY/180 at 36hpi, biological rep3	2903.79
GSM1179373	wdNHBE uninfected at 36hpi, biological rep1	1283.07
GSM1179374	wdNHBE uninfected at 36hpi, biological rep2	1445.09
GSM1179375	wdNHBE uninfected at 36hpi, biological rep3	1333.44

Supplementary Figure 5. Gene Ontology (GO) analysis of TMPRSS2 and CTSB/L genes.

GO Biological Process 2018

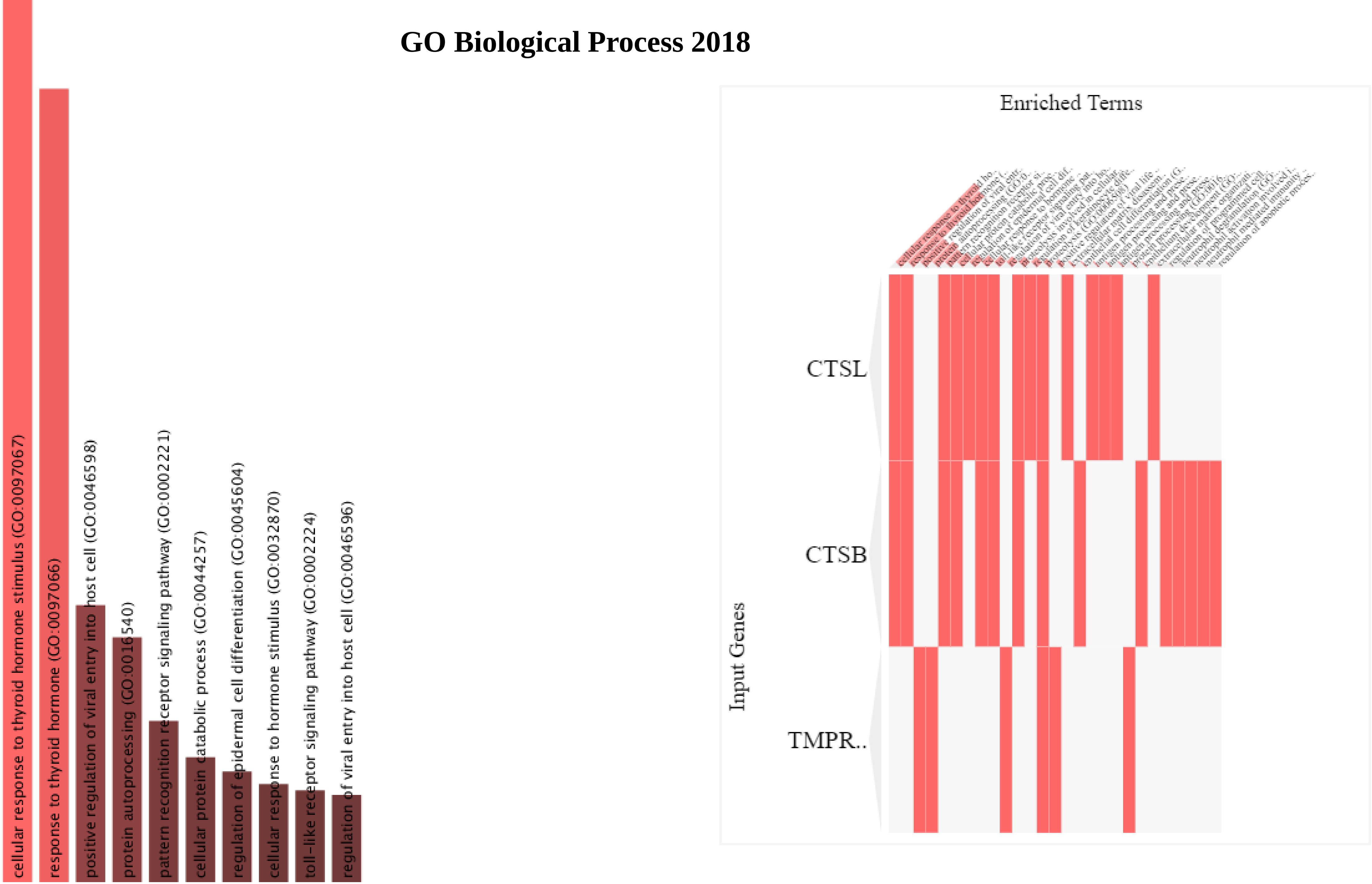

GO Molecular Function 2018

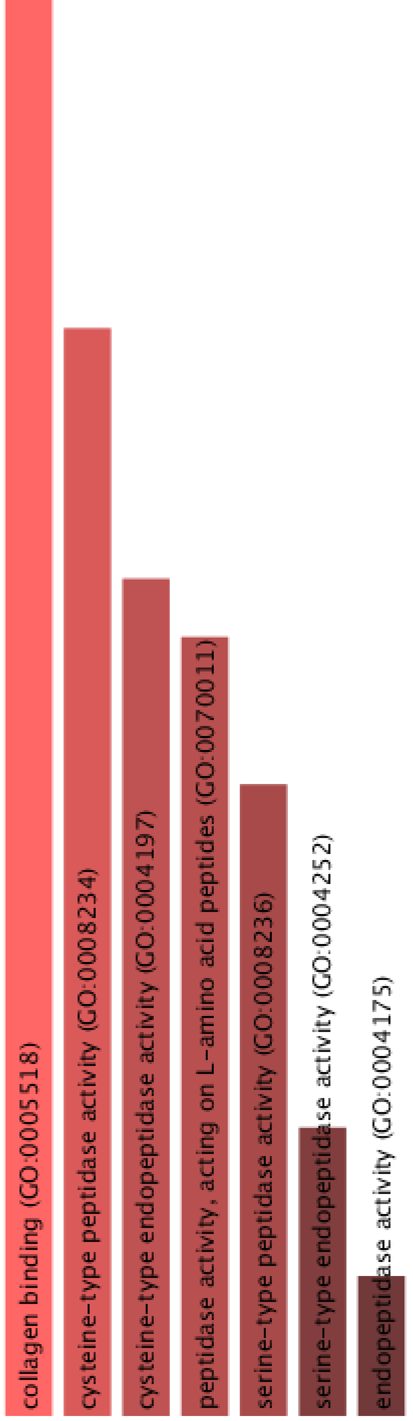

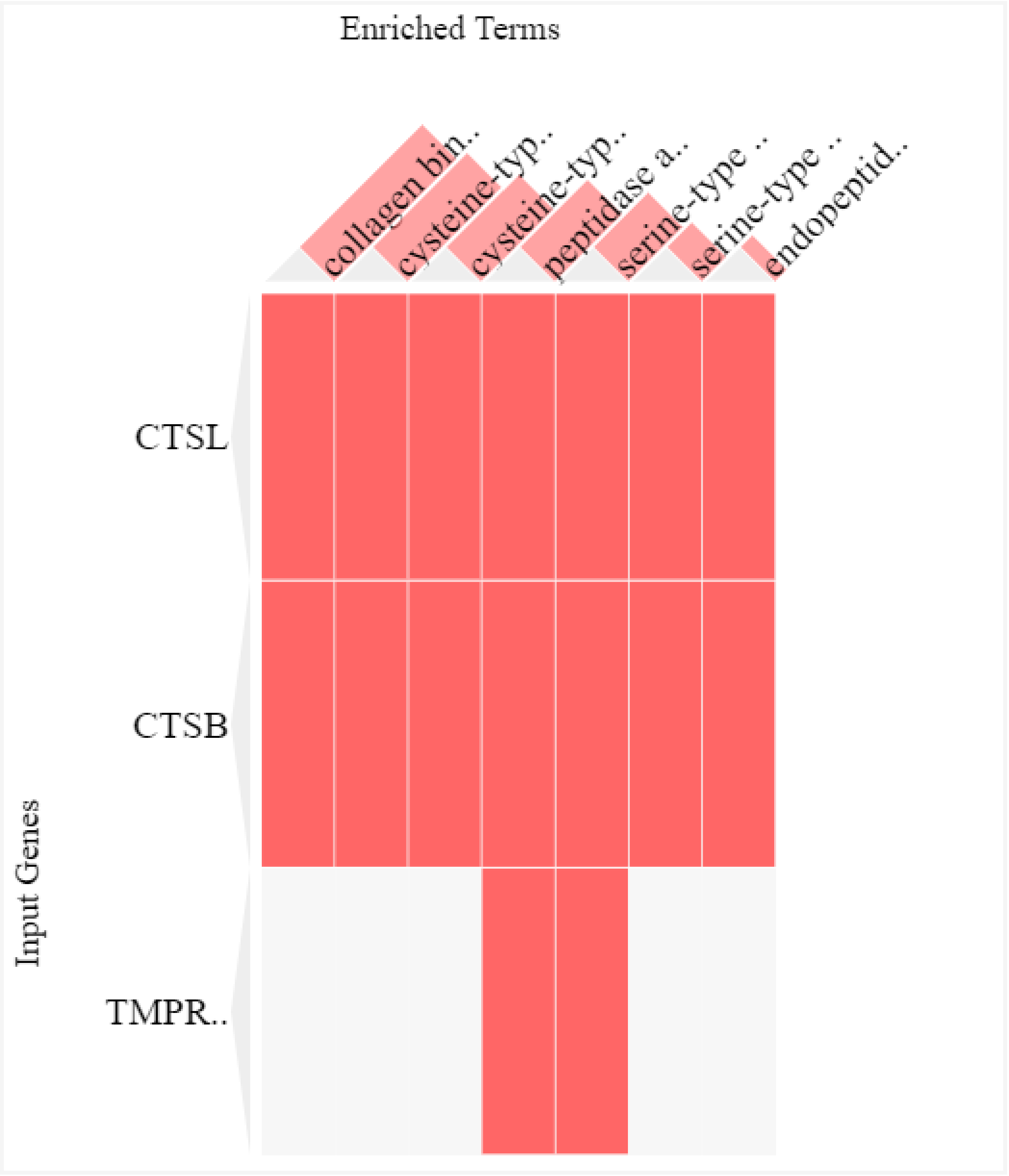

GO Cellular Component 2018

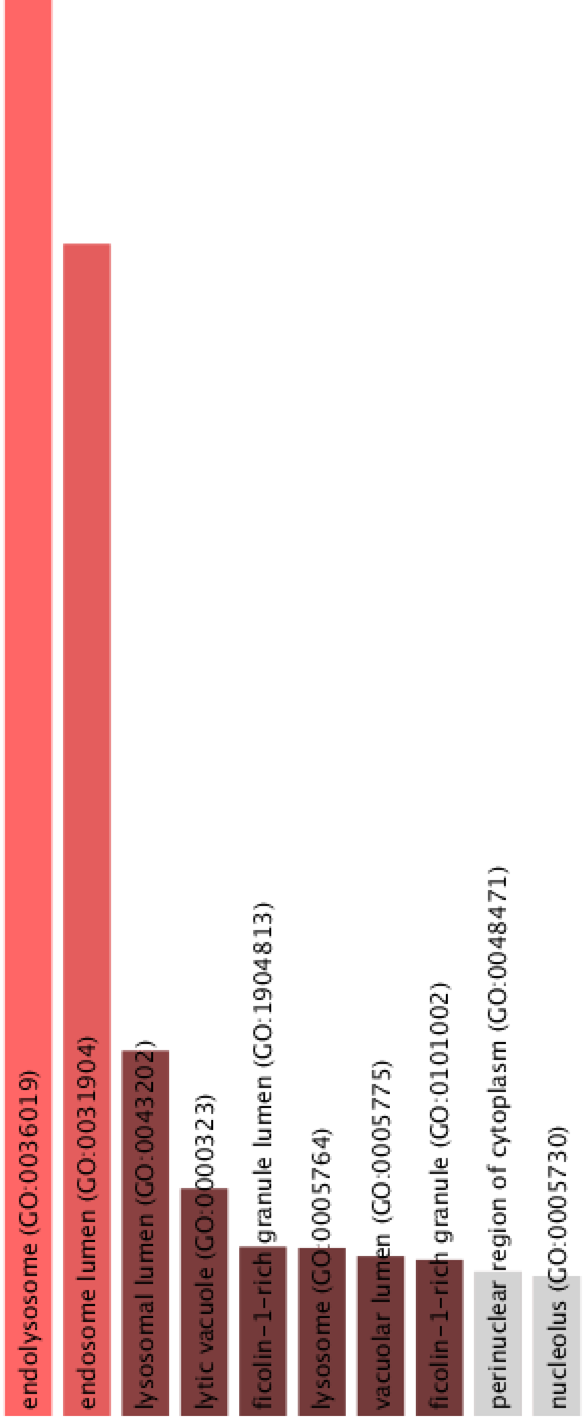

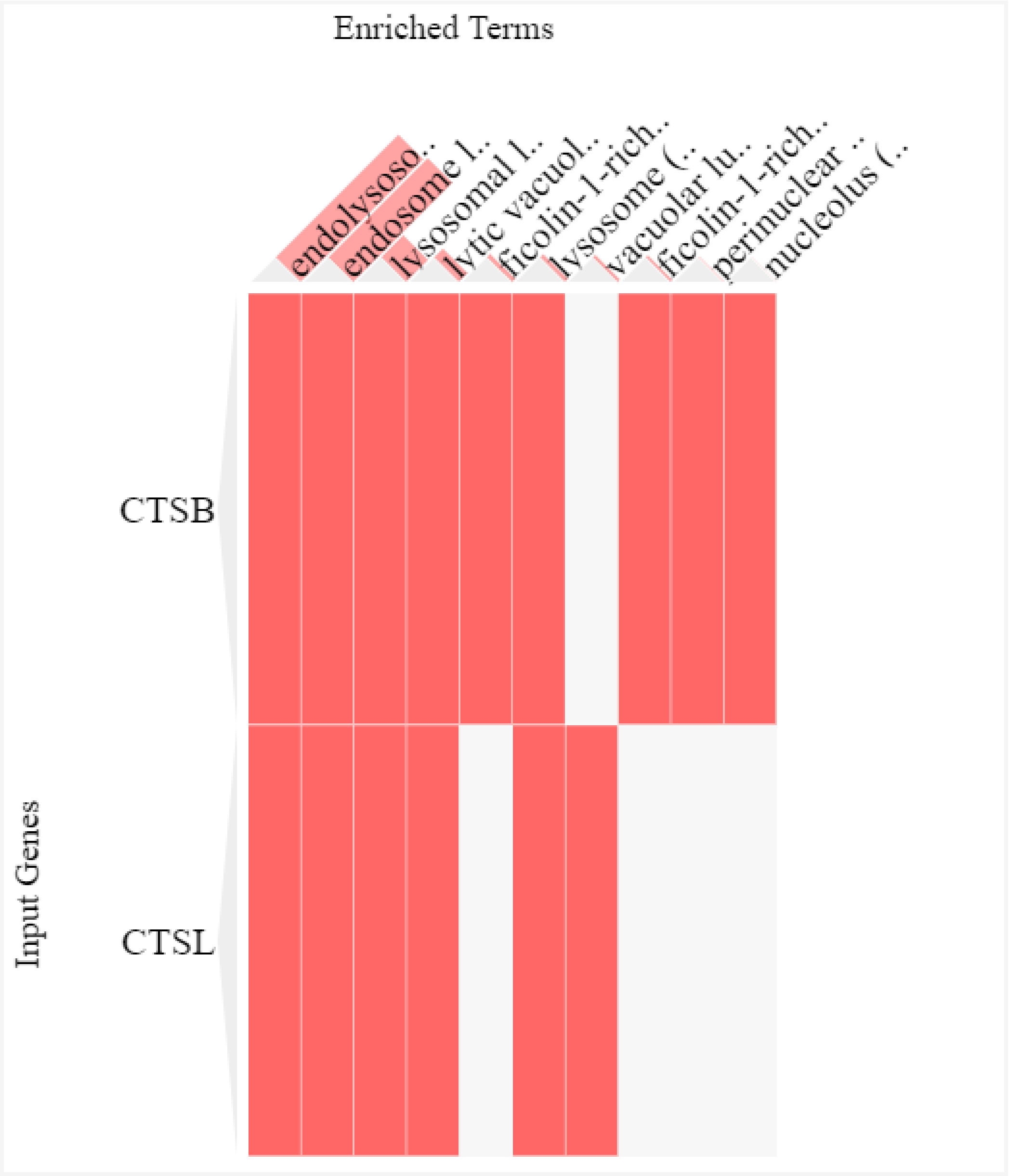

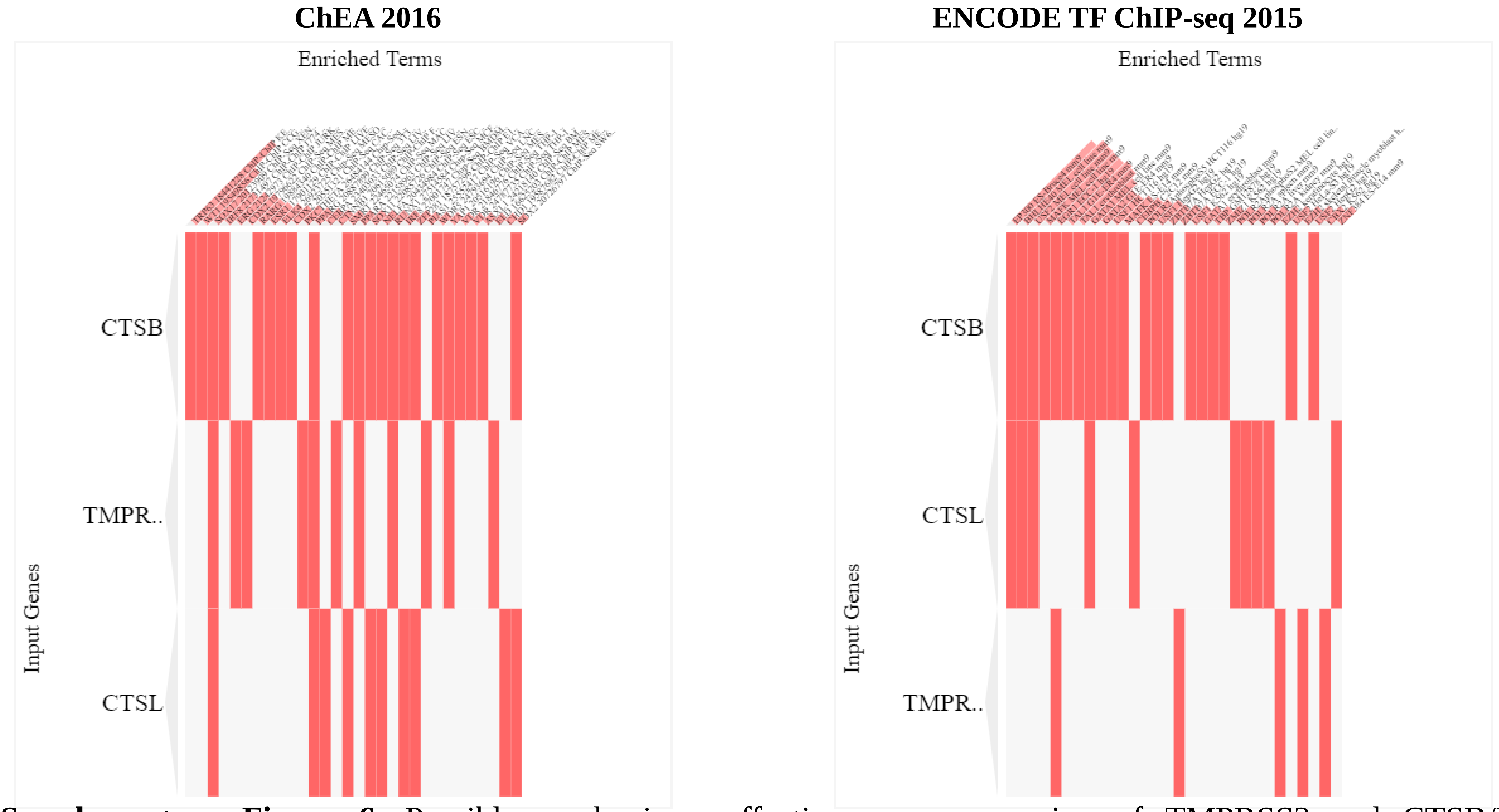

Supplementary Figure 6. Possible mechanisms affecting gene expression of TMPRSS2 and CTSB/L. Identifications of the enriched records of transcription factor-binding sites.

Supplementary Figure 7. Expression of transcription factors affected in SARS infection as identified through enriched GEO records.

PPARG gene is getting downregulated in SARS-CoV-2 Patient

Title: A study of differential circRNA and lncRNA expressions in COVID-19-infected peripheral blood
Organism: Homo Sapiens
GSE-ID: GSE166552

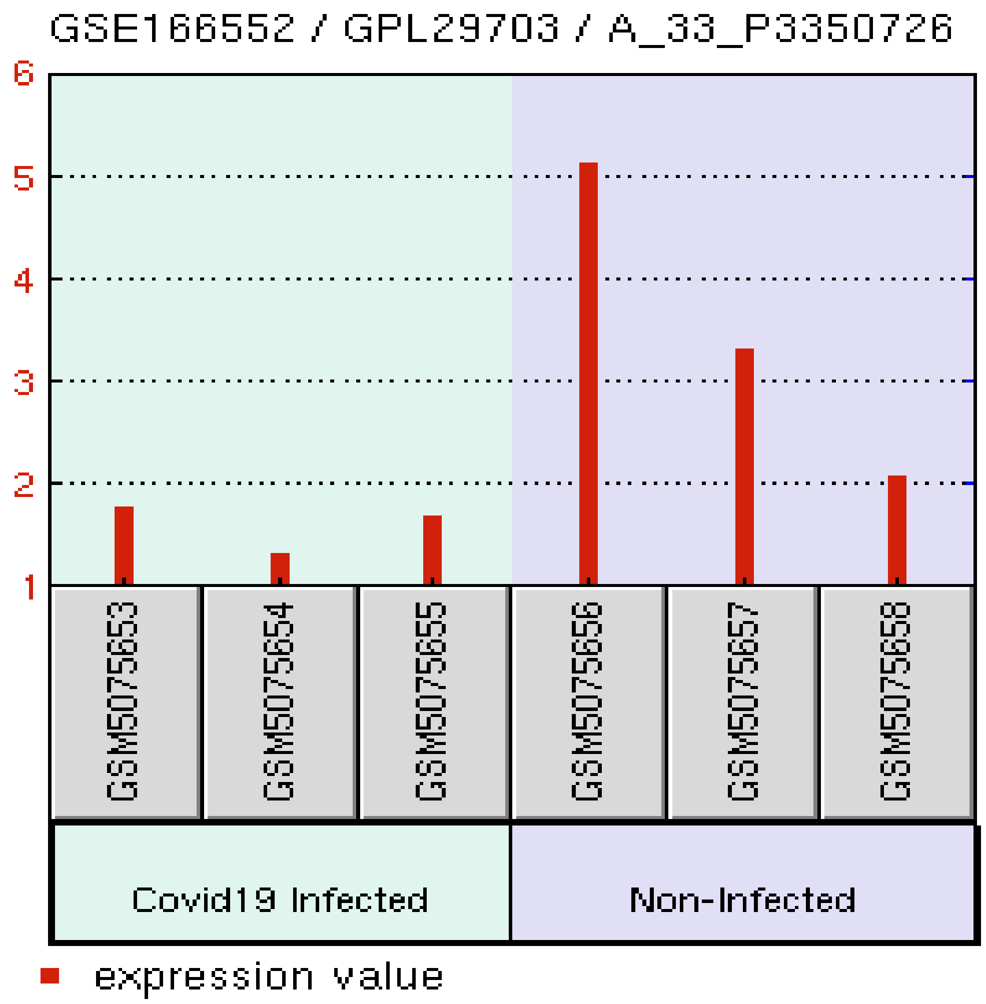

Sample	Title	Value
GSM5075653	P2: COVID-19 patient N0.1	1.79325
GSM5075654	P3: COVID-19 patient N0.2	1.36046
GSM5075655	P9: COVID-19 patient N0.3	1.7006
GSM5075656	P21: Healthy control N0.1	5.13659
GSM5075657	P22: Healthy control N0.2	3.34216
GSM5075658	P23: Healthy control N0.3	2.10922

PPARG - Severe acute respiratory syndrome expression profile

**Profile**	GDS1028 / 208510_s_at
**Title**	Severe acute respiratory syndrome expression profile
**Organism**	Homo sapiens

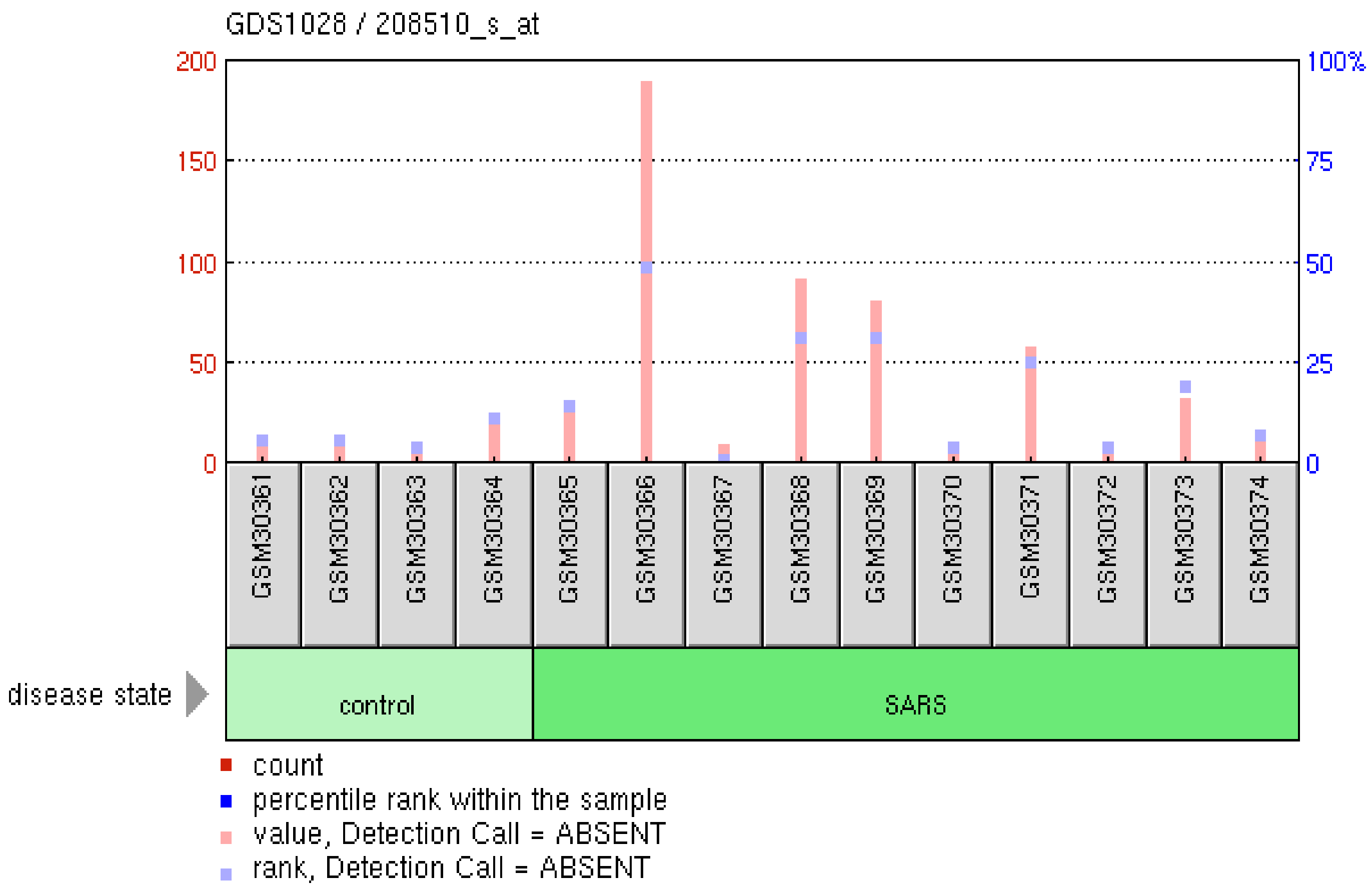

Sample	Title	Value
GSM30361	N1	14.8
GSM30362	N2	11.1
GSM30363	N3	9.5
GSM30364	N4	24.4
GSM30365	S1	28.2
GSM30366	S2	189.4
GSM30367	S3	10.3
GSM30368	S4	91.7
GSM30369	S5	80.9
GSM30370	S6	10.5
GSM30371	S7	58.9
GSM30372	S8	10.9
GSM30373	S9	33
GSM30374	S10	11.5

PPARA downregulated in SARS-CoV-2 Patient

Title: A study of differential circRNA and lncRNA expressions in COVID-19-infected peripheral blood
Organism: Homo Sapiens
GSE-ID: GSE166552

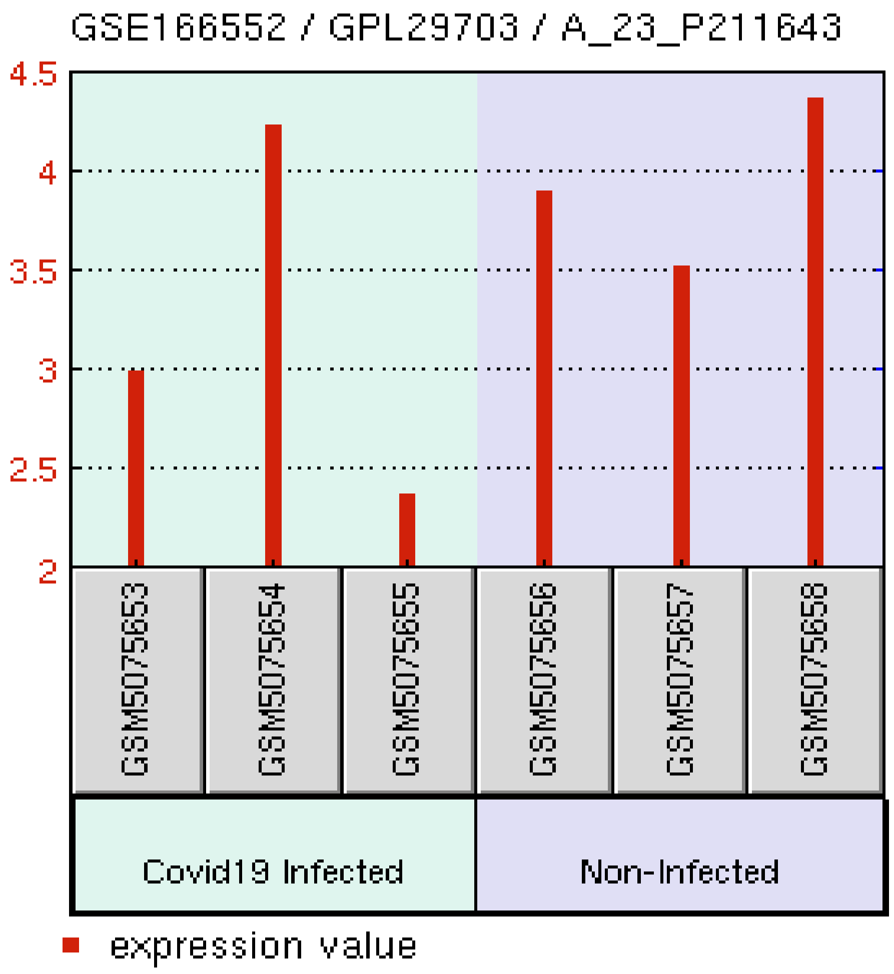

Sample	Title	Value
GSM5075653	P2: COVID-19 patient N0.1	2.99729
GSM5075654	P3: COVID-19 patient N0.2	4.23814
GSM5075655	P9: COVID-19 patient N0.3	2.38785
GSM5075656	P21: Healthy control N0.1	3.90587
GSM5075657	P22: Healthy control N0.2	3.52613
GSM5075658	P23: Healthy control N0.3	4.3785

PPARA - Severe acute respiratory syndrome expression profile

**Profile**	GDS1028 / 210771_at
**Title**	Severe acute respiratory syndrome expression profile
**Organism**	Homo sapiens

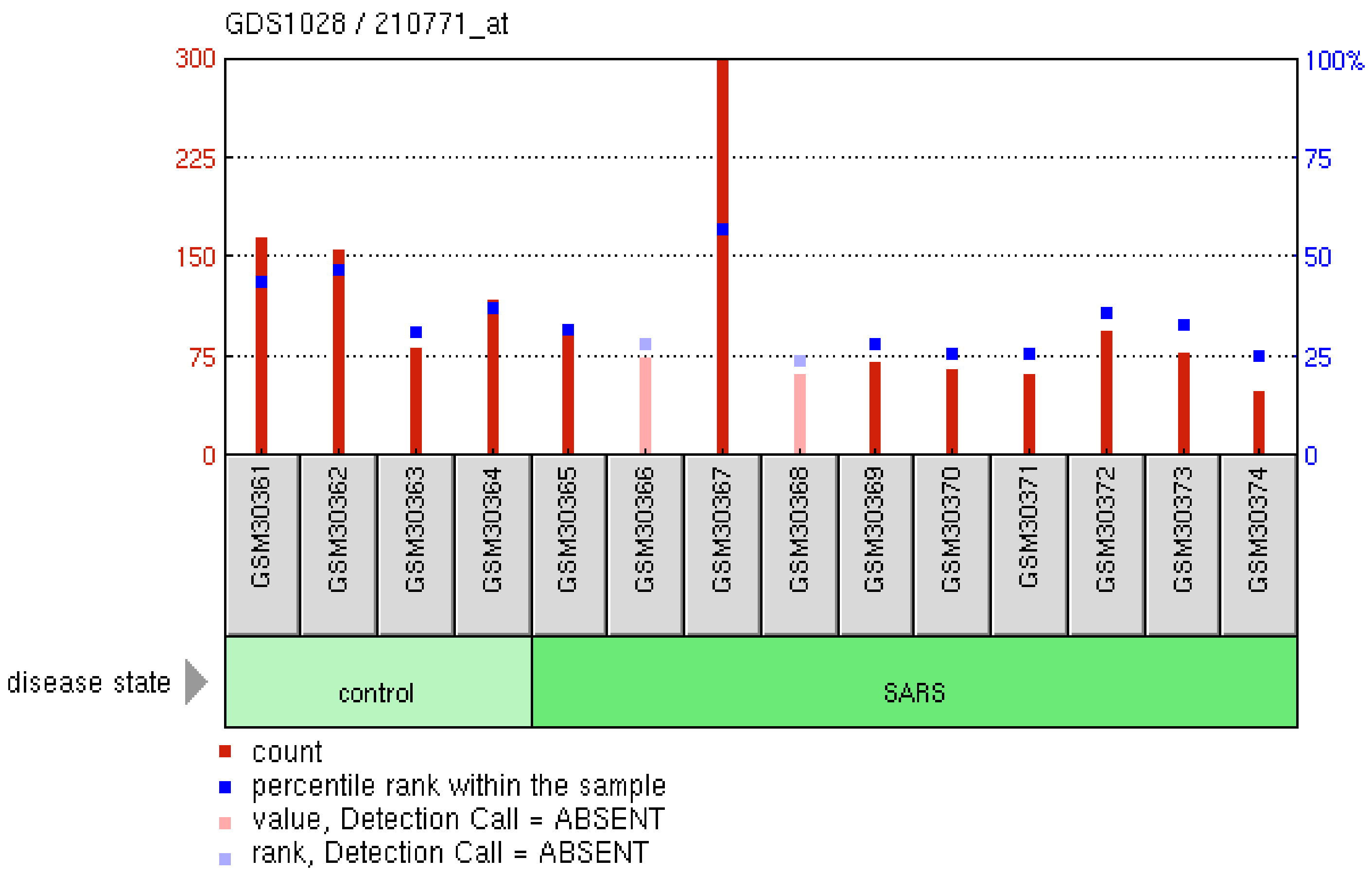

Sample	Title	Value
GSM30361	N1	164.6
GSM30362	N2	156.9
GSM30363	N3	82.7
GSM30364	N4	118.5
GSM30365	S1	95.7
GSM30366	S2	74.4
GSM30367	S3	298.5
GSM30368	S4	62.4
GSM30369	S5	71.4
GSM30370	S6	66.7
GSM30371	S7	61.7
GSM30372	S8	95.7
GSM30373	S9	77.8
GSM30374	S10	50

SOX17 gene is upregulated in SARS-CoV-2 Patient

Title: A study of differential circRNA and lncRNA expressions in COVID-19-infected peripheral blood
Organism: Homo Sapiens
GSE-ID: GSE166552

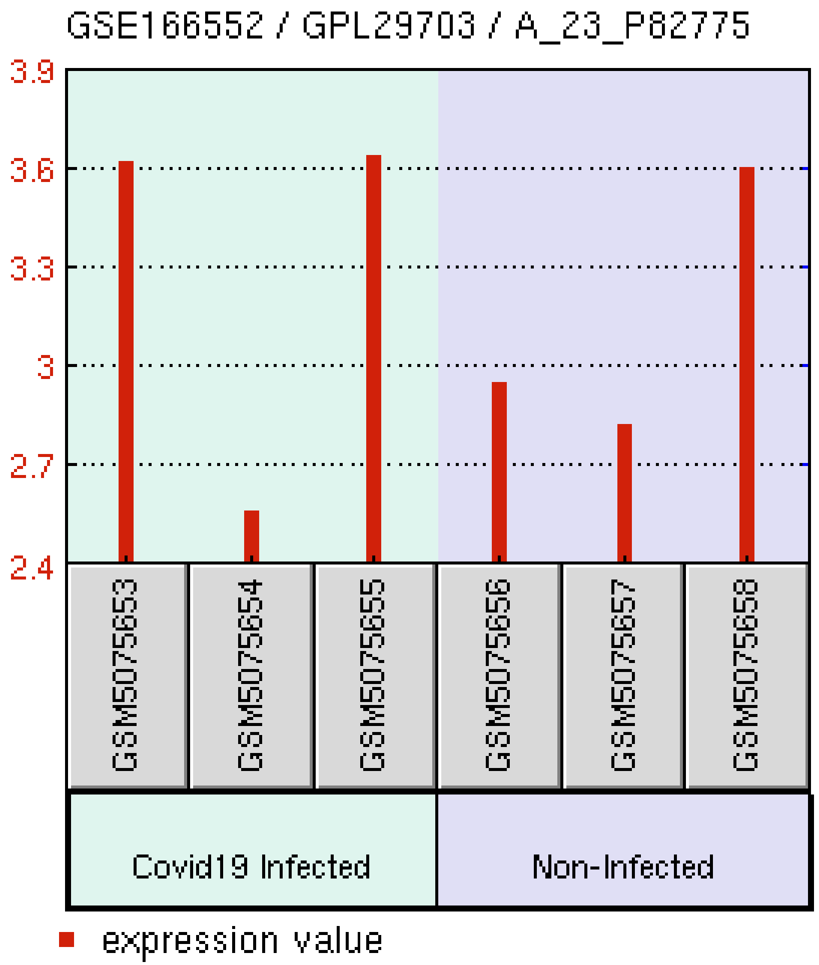

Sample	Title	Value
GSM5075653	P2: COVID-19 patient N0.1	3.62219
GSM5075654	P3: COVID-19 patient N0.2	2.57053
GSM5075655	P9: COVID-19 patient N0.3	3.64608
GSM5075656	P21: Healthy control N0.1	2.95647
GSM5075657	P22: Healthy control N0.2	2.82883
GSM5075658	P23: Healthy control N0.3	3.61072

SOX17 - Severe acute respiratory syndrome expression profile

**Profile**	GDS1028 / 219993_at
**Title**	Severe acute respiratory syndrome expression profile
**Organism**	Homo sapiens

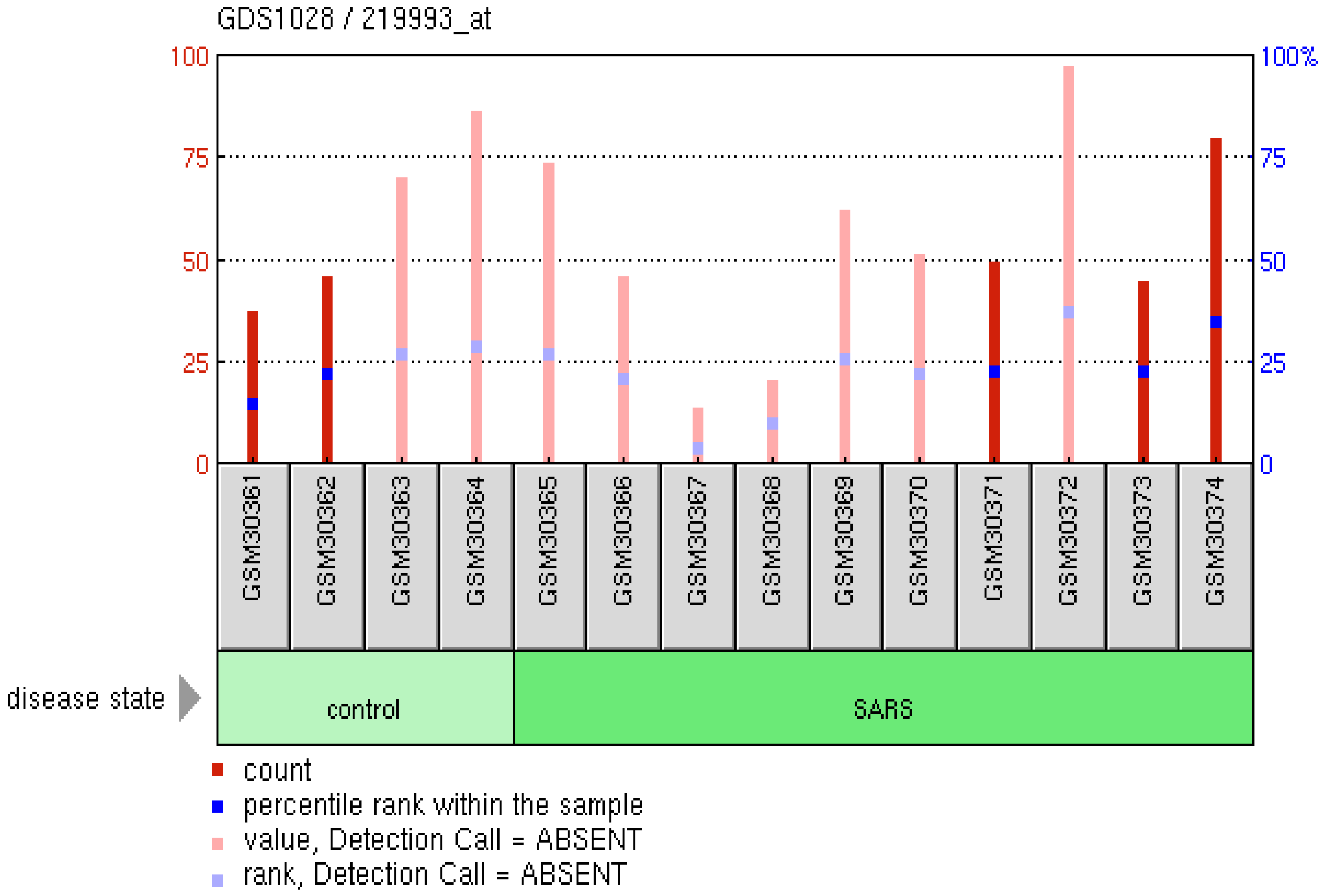

Sample	Title	Value
GSM30361	N1	37.5
GSM30362	N2	46.1
GSM30363	N3	69.9
GSM30364	N4	86.7
GSM30365	S1	74
GSM30366	S2	46.1
GSM30367	S3	14.4
GSM30368	S4	20.5
GSM30369	S5	62.4
GSM30370	S6	51.6
GSM30371	S7	49.9
GSM30372	S8	97.4
GSM30373	S9	45
GSM30374	S10	80.1

RUNX1 gene upregulated in SARS-CoV-2 Patient

Title: A study of differential circRNA and lncRNA expressions in COVID-19-infected peripheral blood
Organism: Homo Sapiens
GSE-ID: GSE166552

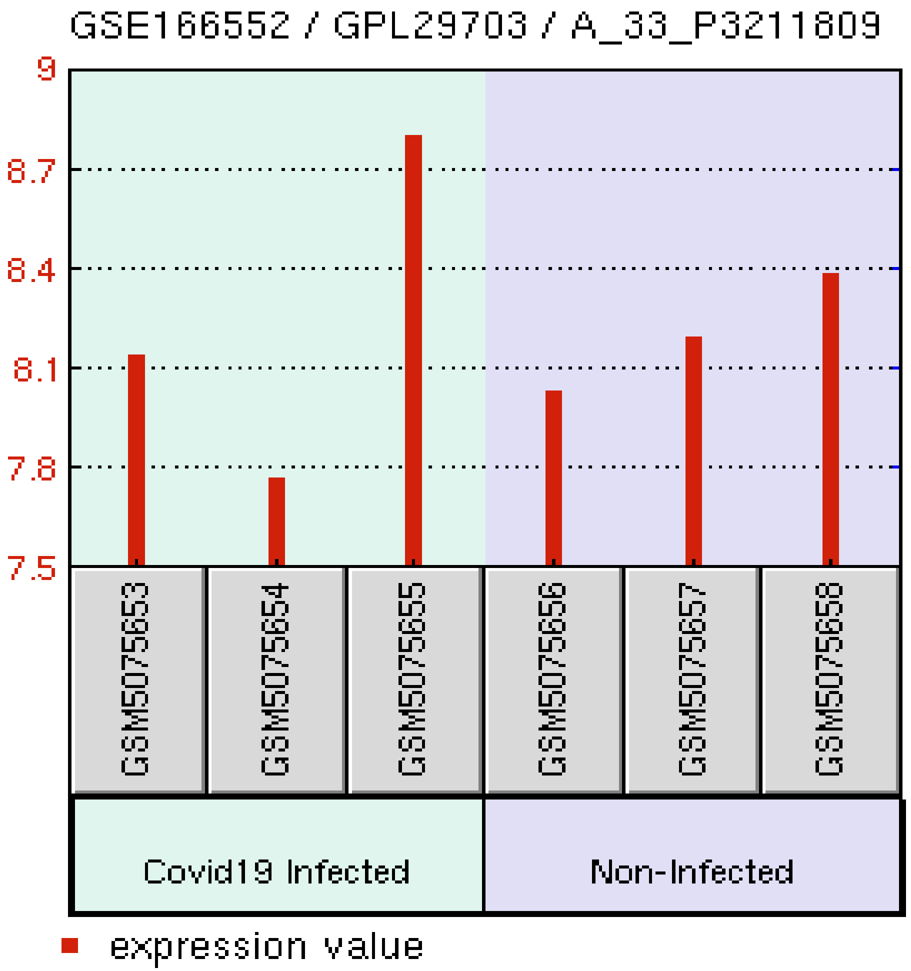

Sample	Title	Value
GSM5075653	P2: COVID-19 patient N0.1	8.14211
GSM5075654	P3: COVID-19 patient N0.2	7.77979
GSM5075655	P9: COVID-19 patient N0.3	8.80459
GSM5075656	P21: Healthy control N0.1	8.03636
GSM5075657	P22: Healthy control N0.2	8.20016
GSM5075658	P23: Healthy control N0.3	8.38974

Supplementary Figure 8. Possible mechanisms affecting gene expression of TMPRSS2 and CTSB/L. Identifications of the enriched GEO records of candidate transcription factors that affect expression of target genes.

Tmprss2 - Effect of macrophage-specific peroxisome proliferator-activated receptor-gamma deficiency on induced inflammatory bowel disease

**Profile**	GDS4370 / 1419154_at
**Title**	Effect of macrophage-specific peroxisome proliferator-activated receptor-gamma deficiency on induced inflammatory bowel disease
**Organism**	Mus musculus

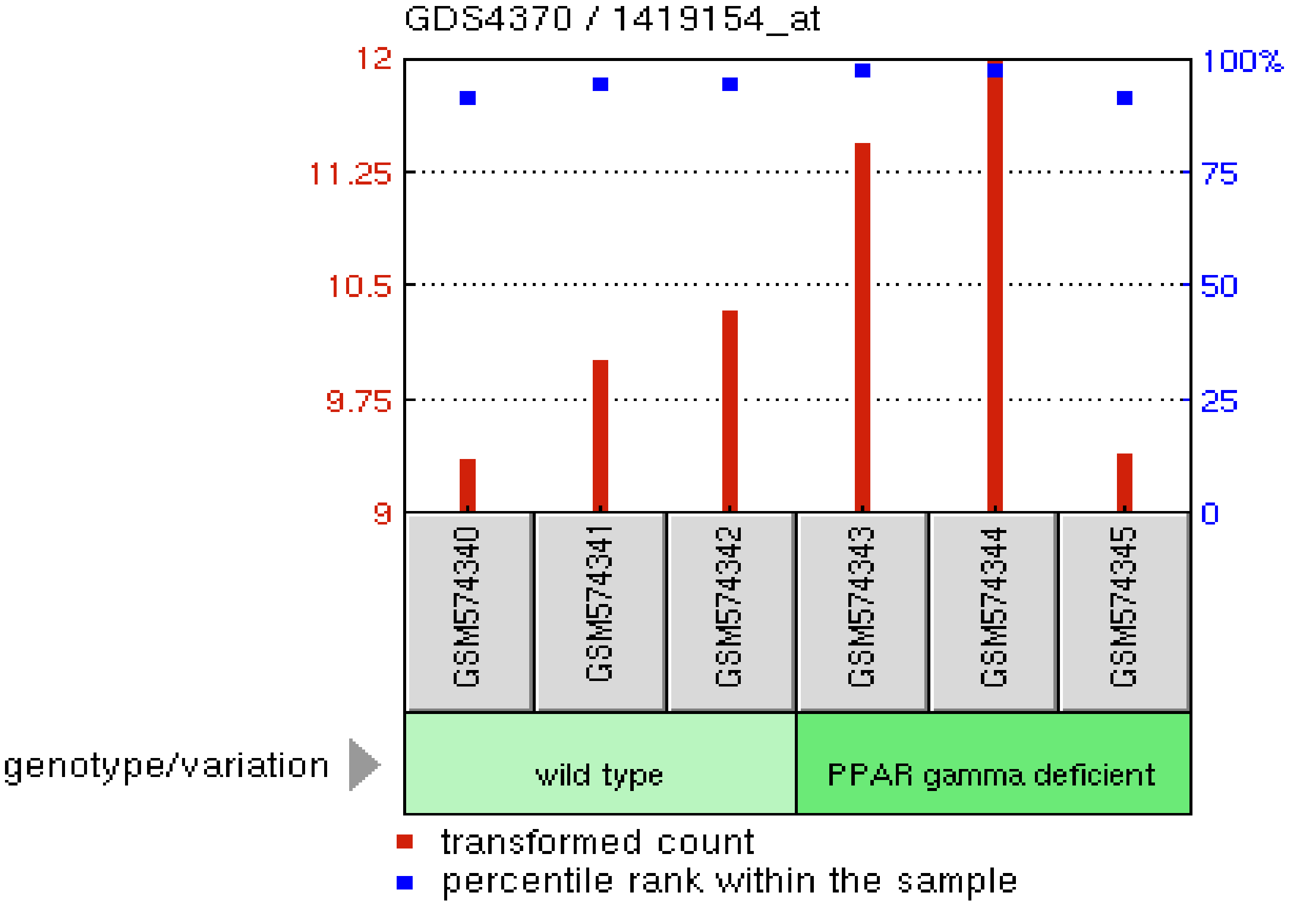

Sample	Title	Value
GSM574340	colonic tissue from floxed mouse, biological rep1	9.37187
GSM574341	colonic tissue from floxed mouse, biological rep2	10.0271
GSM574342	colonic tissue from floxed mouse, biological rep3	10.3411
GSM574343	colonic tissue from macrophage-specific PPARgamma-deficient mouse, biological rep1	11.441
GSM574344	colonic tissue from macrophage-specific PPARgamma-deficient mouse, biological rep2	11.9891
GSM574345	colonic tissue from macrophage-specific PPARgamma-deficient mouse, biological rep3	9.41078

Tmprss2 - Aortic endothelial cell response to dominant negative **PPAR** gamma expression in vitro

Profile GDS3440 / 1458347_s_at

Title Aortic endothelial cell response to dominant negative PPAR gamma expression in vitro

Organism Mus musculus

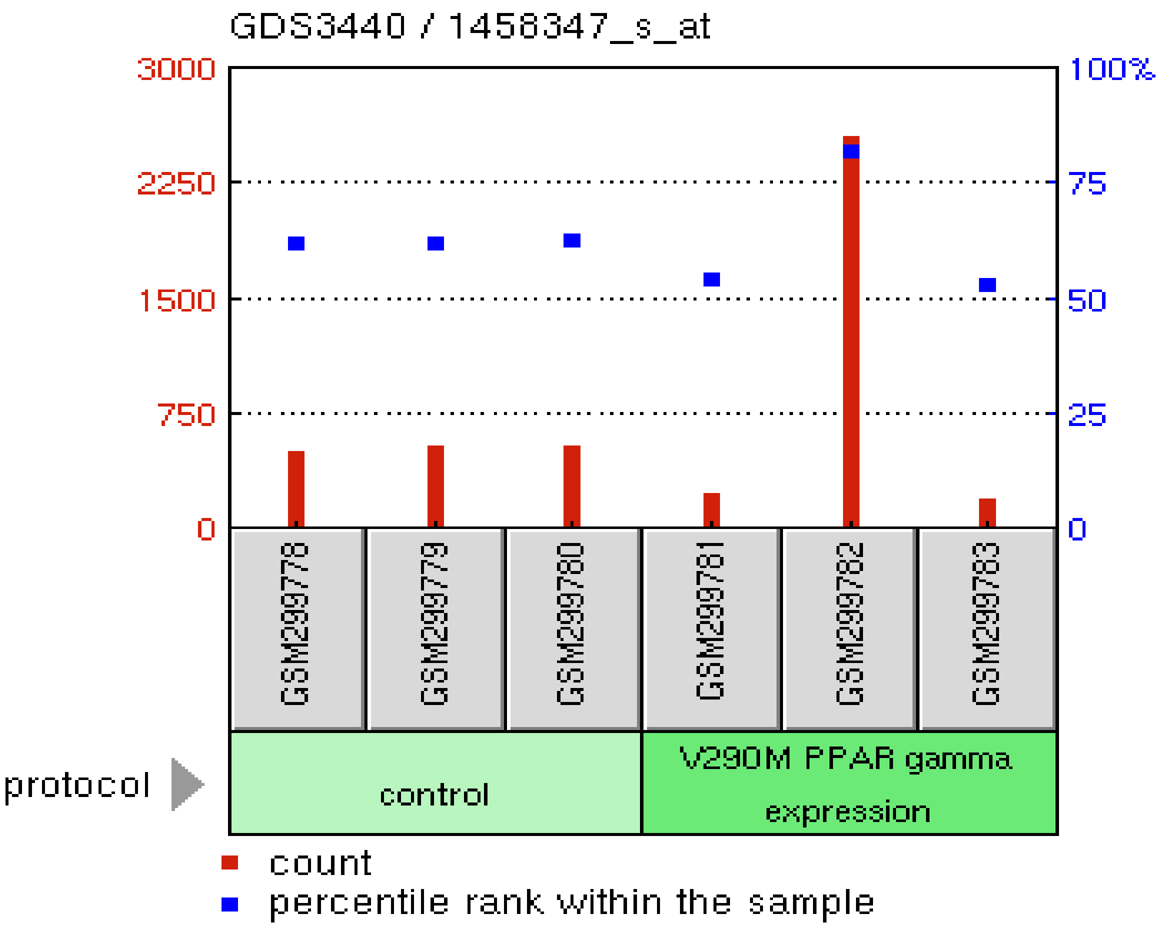

Sample	Title	Value
GSM299778	Primary aortic endothelial cells, Non_transgenic_control_1	516.3
GSM299779	Primary aortic endothelial cells, Non_transgenic_control_2	551.8
GSM299780	Primary aortic endothelial cells, Non_transgenic_control_3	552.3
GSM299781	Primary aortic endothelial cells, transgenic_PPARG_V290M_1	235.1
GSM299782	Primary aortic endothelial cells, transgenic_PPARG_V290M_2	2562.2
GSM299783	Primary aortic endothelial cells, transgenic_PPARG_V290M_3	212.8

Dominant negative PPAR gamma expression **is associated with decreased expression of the *TMPRSS2* gene**

Tmprss2 - Peroxisome proliferator-activated receptor alpha deficiency effect on phthalate-exposed liver

**Profile**	GDS3748 / 10441254
**Title**	Peroxisome proliferator-activated receptor alpha deficiency effect on phthalate-exposed liver
**Organism**	Mus musculus

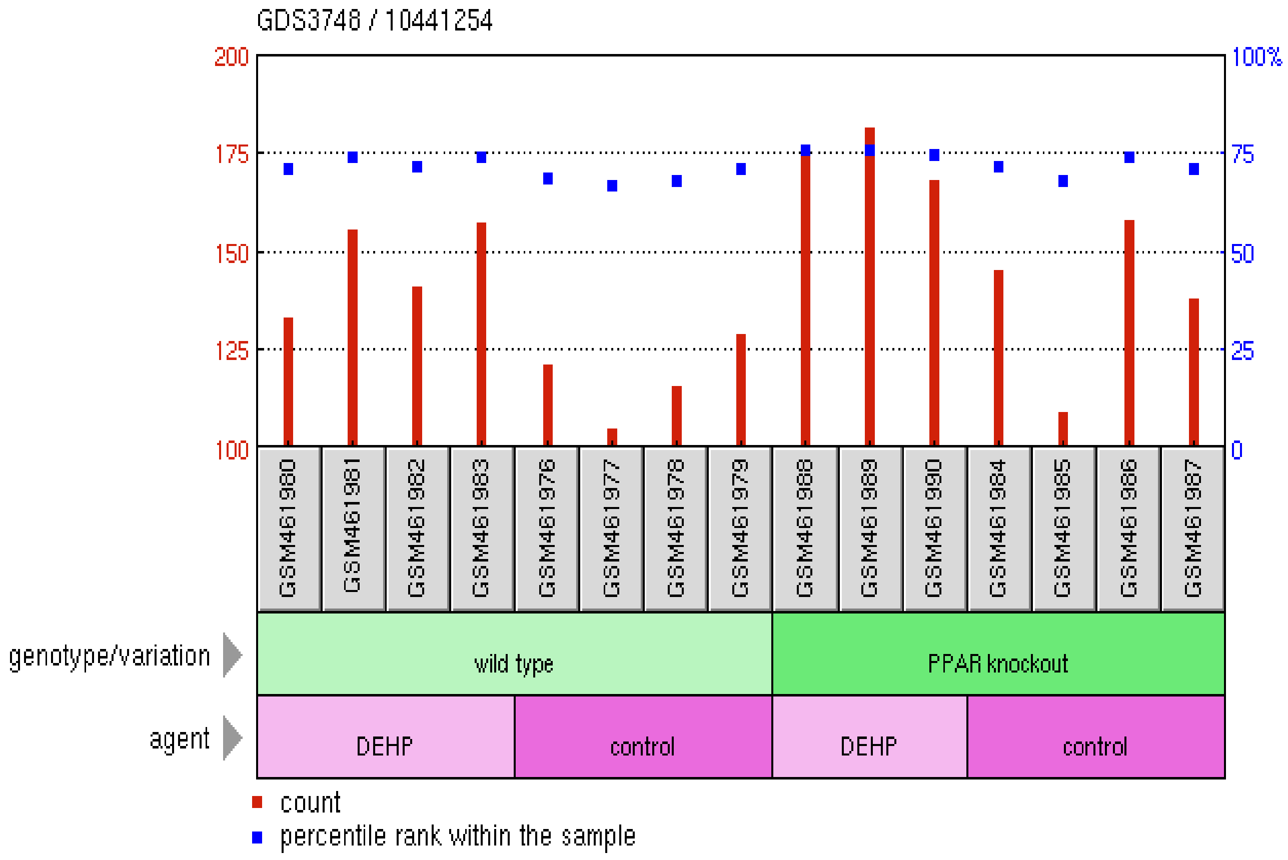

Sample	Title	Value
GSM461980	PPAR WT_DEHP 1150_56	133.32
GSM461981	PPAR WT_DEHP 1150_57	155.45
GSM461982	PPAR WT_DEHP 1150_58	141.3
GSM461983	PPAR WT_DEHP 1150_59	157.43
GSM461976	PPAR WT_ctr_55	121.44
GSM461977	PPAR WT_ctr_54	105.01
GSM461978	PPAR WT_ctr_53	115.83
GSM461979	PPAR WT_ctr_52	129.12
GSM461988	PPAR KO_DEHP 1150_121	176.17
GSM461989	PPAR KO_DEHP 1150_122	181.53
GSM461990	PPAR KO_DEHP 1150_123	168.27
GSM461984	PPAR KO_ctr_115	145.61
GSM461985	PPAR KO_ctr_116	109.6
GSM461986	PPAR KO_ctr_117	157.88
GSM461987	PPAR KO_ctr_118	138

PPAR-alpha knockout **is associated with increased expression of the *TMPRSS2* gene**

Ctsb - Aortic endothelial cell response to dominant negative **PPAR** gamma expression in vitro

**Profile**	GDS3440 / 1444987_at
**Title**	Aortic endothelial cell response to dominant negative PPAR gamma expression in vitro
**Organism**	Mus musculus

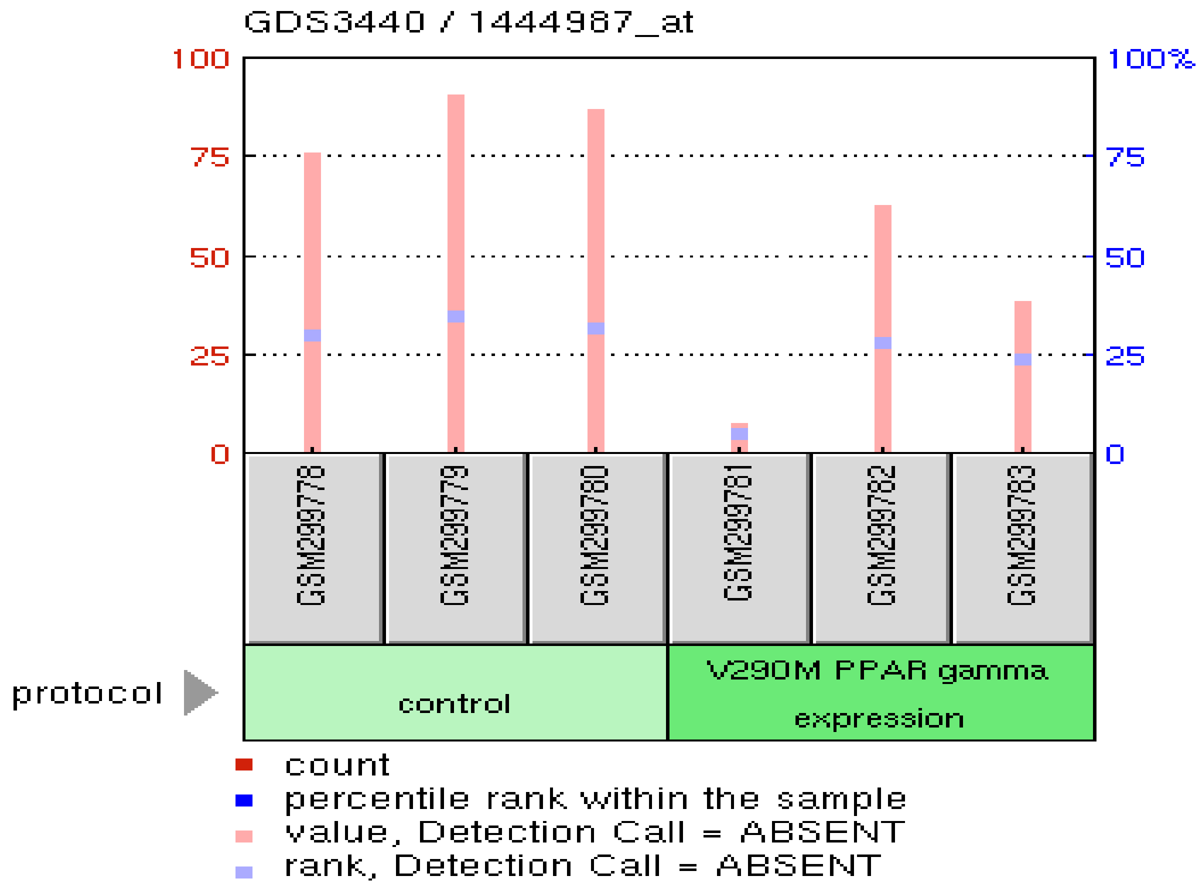

Sample	Title	Value
GSM299778	Primary aortic endothelial cells, Non_transgenic_control_1	76.4
GSM299779	Primary aortic endothelial cells, Non_transgenic_control_2	90.9
GSM299780	Primary aortic endothelial cells, Non_transgenic_control_3	86.9
GSM299781	Primary aortic endothelial cells, transgenic_PPARG_V290M_1	8
GSM299782	Primary aortic endothelial cells, transgenic_PPARG_V290M_2	62.8
GSM299783	Primary aortic endothelial cells, transgenic_PPARG_V290M_3	38.8

***PPAR gamma negative expression* is associated with decreased expression of the *CTSB* gene**

Ctsb - Peroxisome proliferator-activated receptor alpha deficiency effect on phthalate-exposed liver

**Profile**	GDS3748 / 10415844
**Title**	Peroxisome proliferator-activated receptor alpha deficiency effect on phthalate-exposed liver
**Organism**	Mus musculus

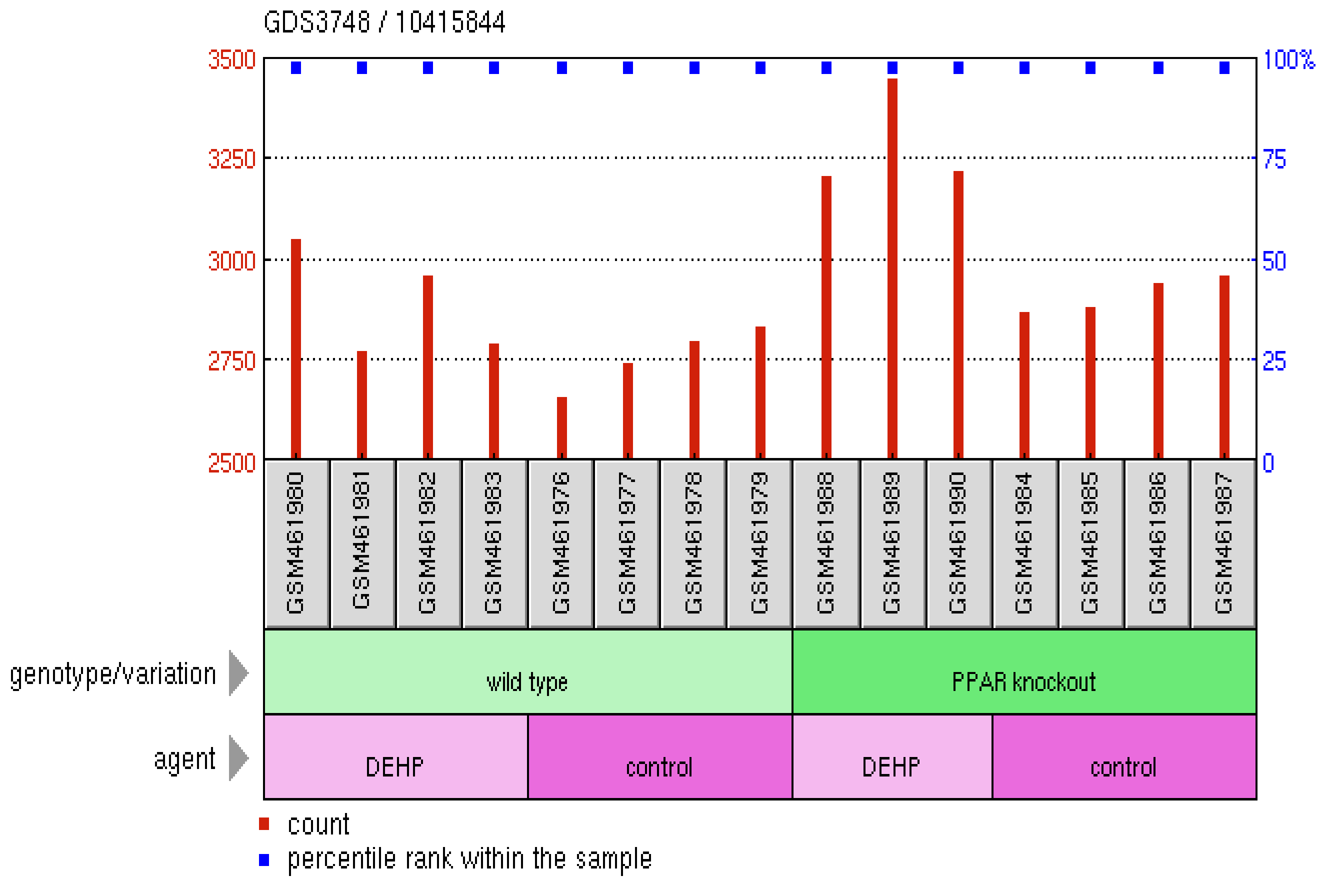

Sample	Title	Value
GSM461980	PPAR WT_DEHP 1150_56	3050.88
GSM461981	PPAR WT_DEHP 1150_57	2773.03
GSM461982	PPAR WT_DEHP 1150_58	2961.95
GSM461983	PPAR WT_DEHP 1150_59	2793.83
GSM461976	PPAR WT_ctr_55	2660.3
GSM461977	PPAR WT_ctr_54	2745.3
GSM461978	PPAR WT_ctr_53	2798.25
GSM461979	PPAR WT_ctr_52	2835.57
GSM461988	PPAR KO_DEHP 1150_121	3210.83
GSM461989	PPAR KO_DEHP 1150_122	3446.7
GSM461990	PPAR KO_DEHP 1150_123	3221.99
GSM461984	PPAR KO_ctr_115	2870.97
GSM461985	PPAR KO_ctr_116	2879.53
GSM461986	PPAR KO_ctr_117	2940.73
GSM461987	PPAR KO_ctr_118	2958.05

PPAR-alpha knockout **is associated with increased expression of the** ***CTSB*** **gene**

Ctsl - Aortic endothelial cell response to dominant negative PPAR gamma expression in vitro

**Profile**	GDS3440 / 1457724_at
**Title**	Aortic endothelial cell response to dominant negative PPAR gamma expression in vitro
**Organism**	Mus musculus

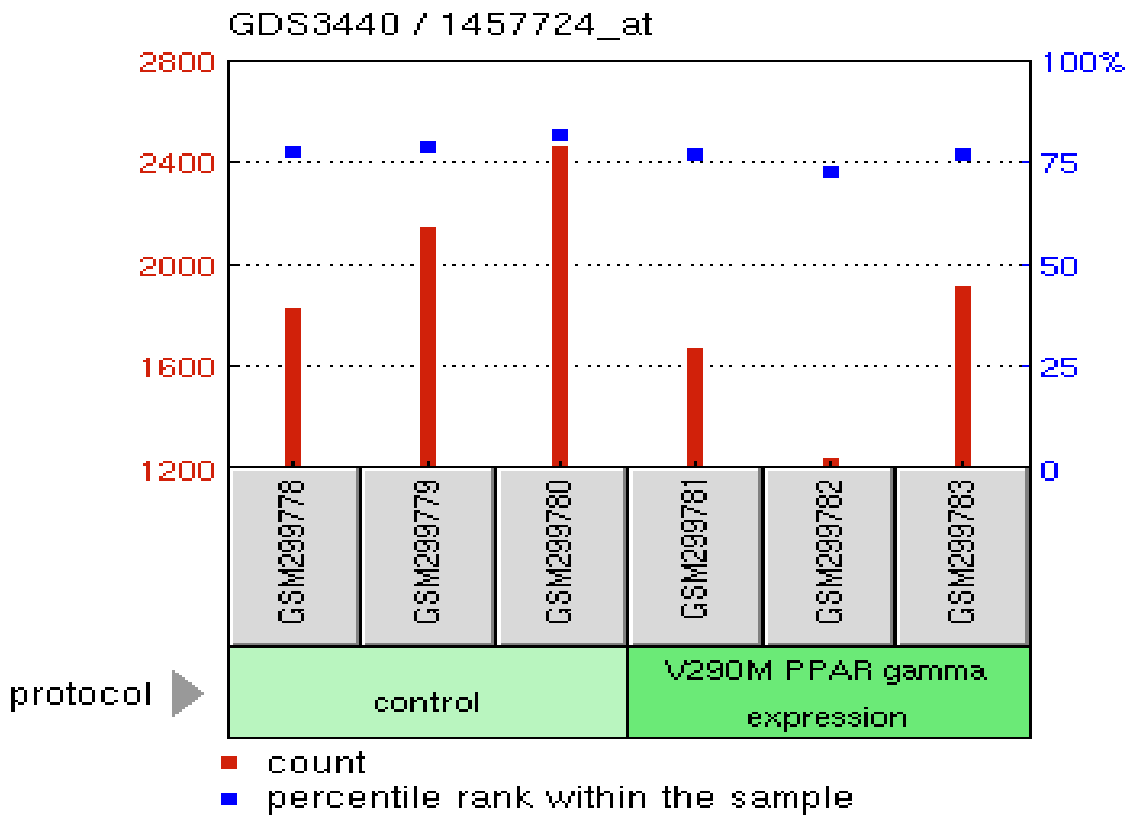

Sample	Title	Value
GSM299778	Primary aortic endothelial cells, Non_transgenic_control_1	1834.6
GSM299779	Primary aortic endothelial cells, Non_transgenic_control_2	2154.1
GSM299780	Primary aortic endothelial cells, Non_transgenic_control_3	2469.3
GSM299781	Primary aortic endothelial cells, transgenic_PPARG_V290M_1	1675.9
GSM299782	Primary aortic endothelial cells, transgenic_PPARG_V290M_2	1214.7
GSM299783	Primary aortic endothelial cells, transgenic_PPARG_V290M_3	1915.2

***PPAR gamma negative expression* is associated with decreased expression of the *CTSL* gene**

Ctsl - Peroxisome proliferator-activated receptor alpha deficiency effect on phthalate-exposed liver

**Profile**	GDS3748 / 10410124
**Title**	Peroxisome proliferator-activated receptor alpha deficiency effect on phthalate-exposed liver
**Organism**	Mus musculus

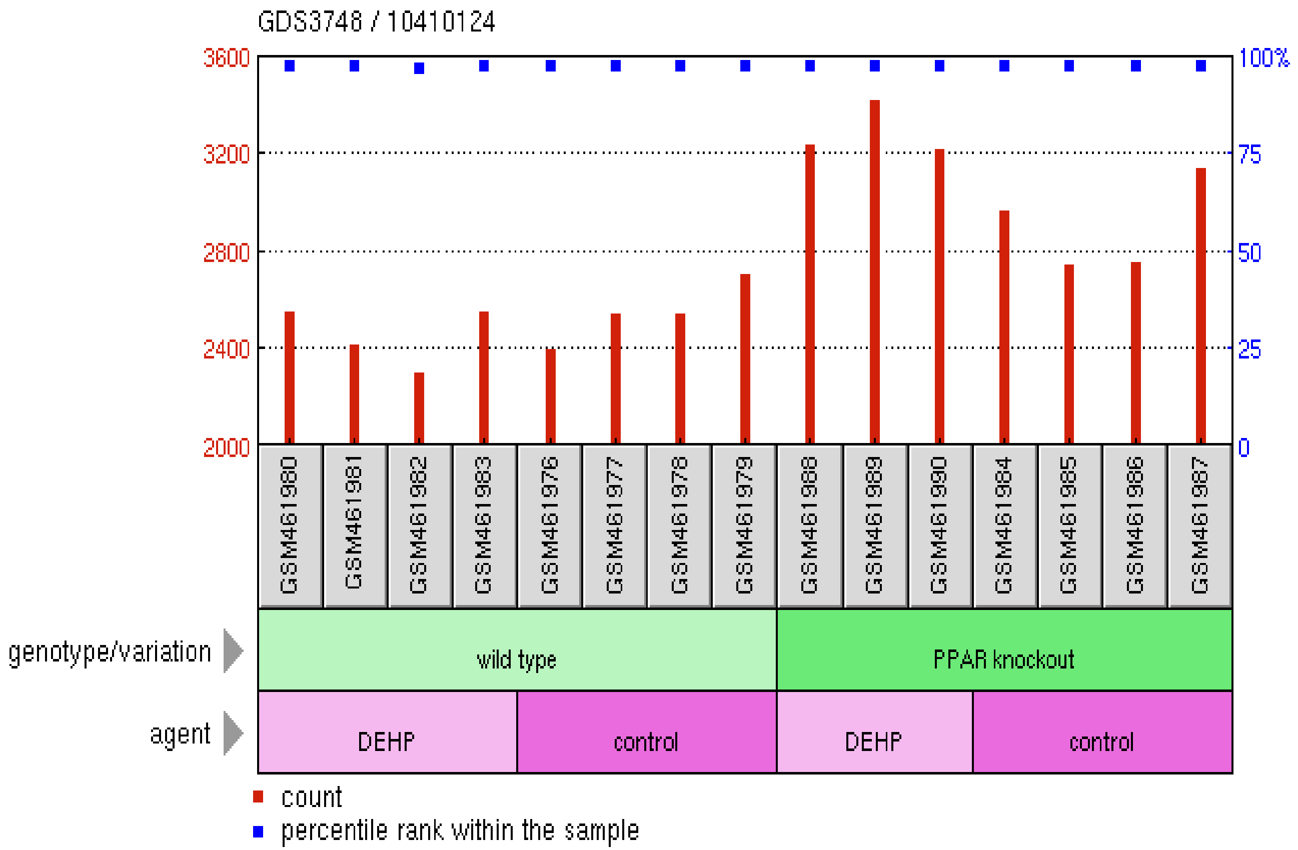

Sample	Title	Value
GSM461980	PPAR WT_DEHP 1150_56	2551.4
GSM461981	PPAR WT_DEHP 1150_57	2418.97
GSM461982	PPAR WT_DEHP 1150_58	2300.34
GSM461983	PPAR WT_DEHP 1150_59	2558.59
GSM461976	PPAR WT_ctr_55	2404.35
GSM461977	PPAR WT_ctr_54	2547.77
GSM461978	PPAR WT_ctr_53	2544.5
GSM461979	PPAR WT_ctr_52	2706.45
GSM461988	PPAR KO_DEHP 1150_121	3233.75
GSM461989	PPAR KO_DEHP 1150_122	3423.84
GSM461990	PPAR KO_DEHP 1150_123	3221.37
GSM461984	PPAR KO_ctr_115	2970.05
GSM461985	PPAR KO_ctr_116	2746.06
GSM461986	PPAR KO_ctr_117	2755.1
GSM461987	PPAR KO_ctr_118	3142.8

PPAR-alpha knockout **is associated with increased expression of the** ***CTSL*** **gene**

TMPRSS2 - SOX transcription factor overexpression in embryonic stem cells

**Profile**	GDS3300 / 226553_at
**Title**	SOX transcription factor overexpression in embryonic stem cells
**Organism**	Homo sapiens

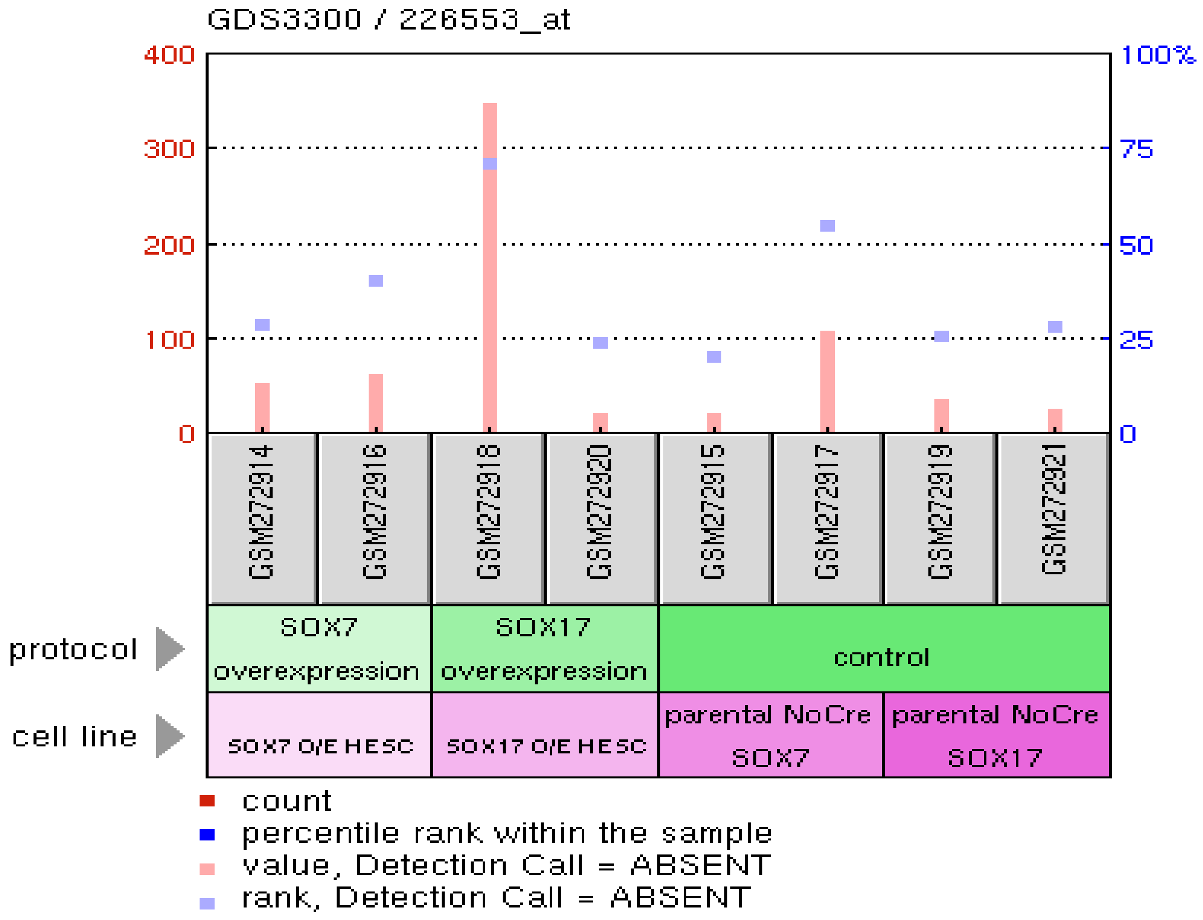

Sample	Title	Value
GSM272914	CA1 Cre Sox7H	53.3074
GSM272916	CA1 Cre Sox7I	63.4244
GSM272918	CA2 Cre Sox17D	347.613
GSM272920	CA2 Cre Sox17E	24.0521
GSM272915	CA1 NoCre Sox7H	24.0059
GSM272917	CA1 NoCre Sox7I	109.408
GSM272919	CA2 NoCre Sox17D	38.2221
GSM272921	CA2 NoCre Sox17E	28.0096

CTSB - SOX transcription factor overexpression in embryonic stem cells

**Profile**	GDS3300 / 200839_s_at
**Title**	SOX transcription factor overexpression in embryonic stem cells
**Organism**	Homo sapiens

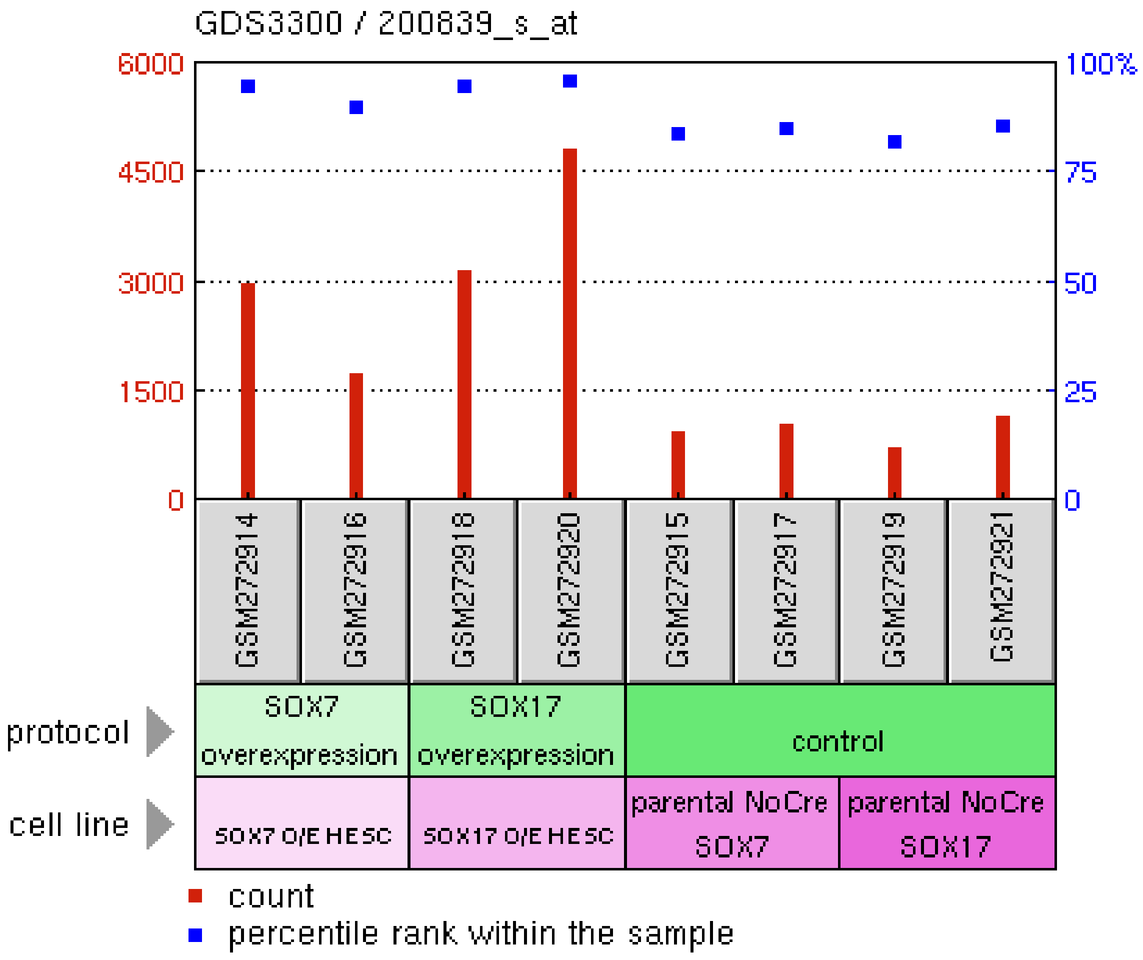

Sample	Title	Value
GSM272914	CA1 Cre Sox7H	2985.73
GSM272916	CA1 Cre Sox7I	1757.14
GSM272918	CA2 Cre Sox17D	3158.22
GSM272920	CA2 Cre Sox17E	4825.36
GSM272915	CA1 NoCre Sox7H	955.038
GSM272917	CA1 NoCre Sox7I	1049.2
GSM272919	CA2 NoCre Sox17D	732.798
GSM272921	CA2 NoCre Sox17E	1173.15

CTSL - SOX transcription factor overexpression in embryonic stem cells

**Profile**	GDS3300 / 202087_s_at
**Title**	SOX transcription factor overexpression in embryonic stem cells
**Organism**	Homo sapiens

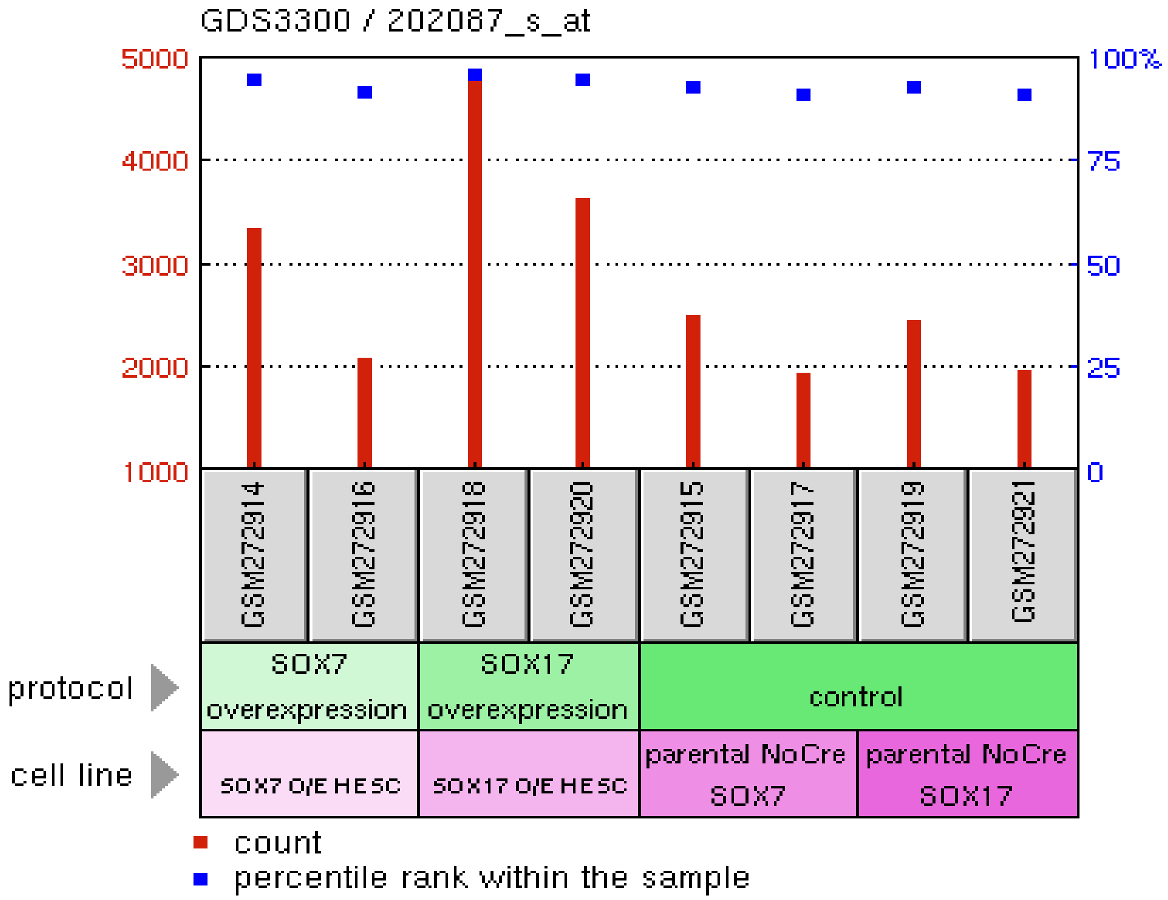

Sample	Title	Value
GSM272914	CA1 Cre Sox7H	3344.16
GSM272916	CA1 Cre Sox7I	2108.18
GSM272918	CA2 Cre Sox17D	4759.38
GSM272920	CA2 Cre Sox17E	3631.73
GSM272915	CA1 NoCre Sox7H	2497.88
GSM272917	CA1 NoCre Sox7I	1944.45
GSM272919	CA2 NoCre Sox17D	2455.53
GSM272921	CA2 NoCre Sox17E	1980.77

Tmprss2 - Transcription factor RUNX1 knockout

**Profile**	GDS1511 / 1449369_at
**Title**	Transcription factor RUNX1 knockout
**Organism**	Mus musculus

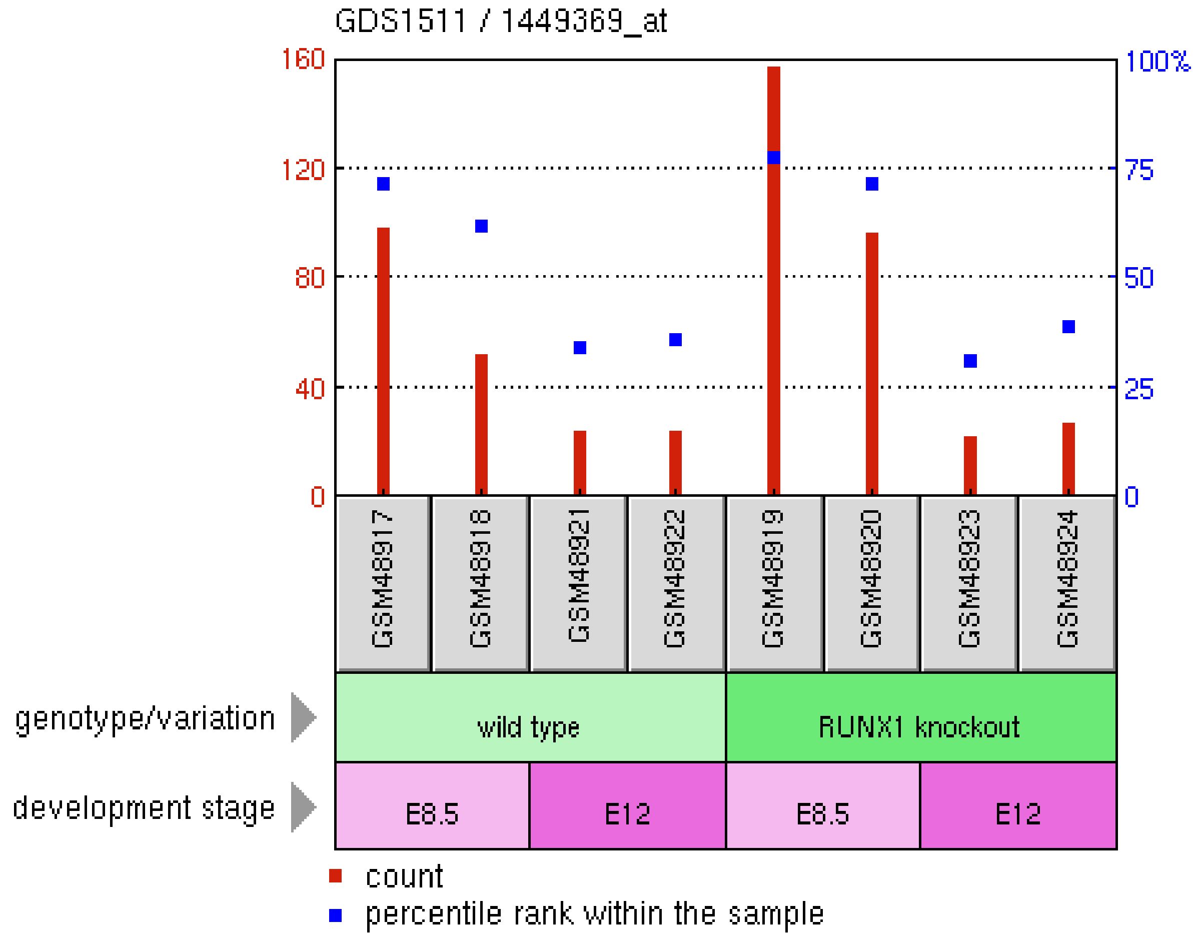

Sample	Title	Value
GSM48917	E8.5 wt.1	98.5563
GSM48918	E8.5 wt.2	52.2892
GSM48921	E12 wt.1	24.4254
GSM48922	E12 wt.2	24.3728
GSM48919	E8.5 ko.1	157.13
GSM48920	E8.5 ko.2	97.1358
GSM48923	E12 ko.1	22.3922
GSM48924	E12 ko.2	27.6408

[**Ctsb** - Transcription factor **RUNX1** knockout]

**Profile**	GDS1511 / 1444987_at
**Title**	Transcription factor RUNX1 knockout
**Organism**	Mus musculus

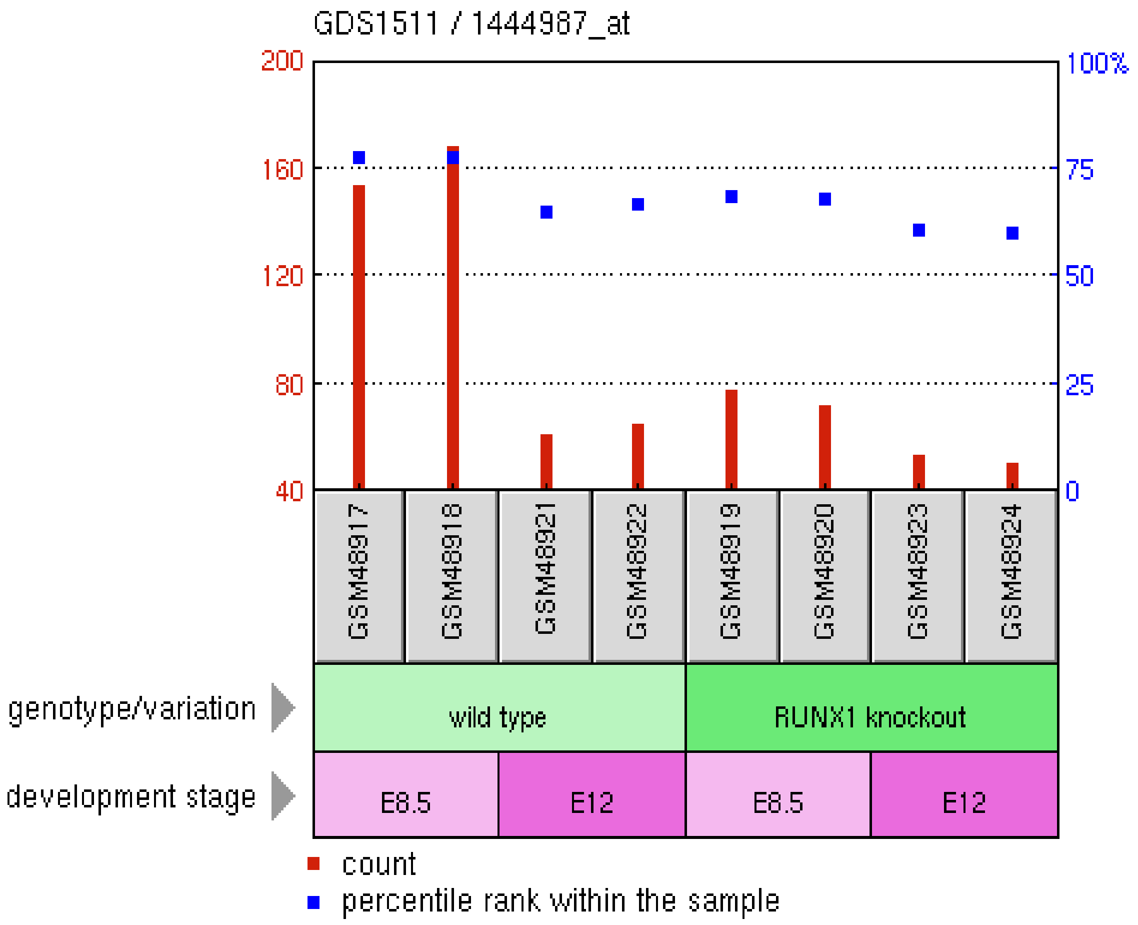

Sample	Title	Value
**GSM48917**	E8.5 wt.1	154.576
**GSM48918**	E8.5 wt.2	168.388
**GSM48921**	E12 wt.1	61.9214
**GSM48922**	E12 wt.2	65.5225
**GSM48919**	E8.5 ko.1	77.9184
**GSM48920**	E8.5 ko.2	72.6096
**GSM48923**	E12 ko.1	53.62
**GSM48924**	E12 ko.2	51.1008

Ctsl - Transcription factor **RUNX1** knockout

**Profile**	GDS1511 / 1457724_at
**Title**	Transcription factor RUNX1 knockout
**Organism**	Mus musculus

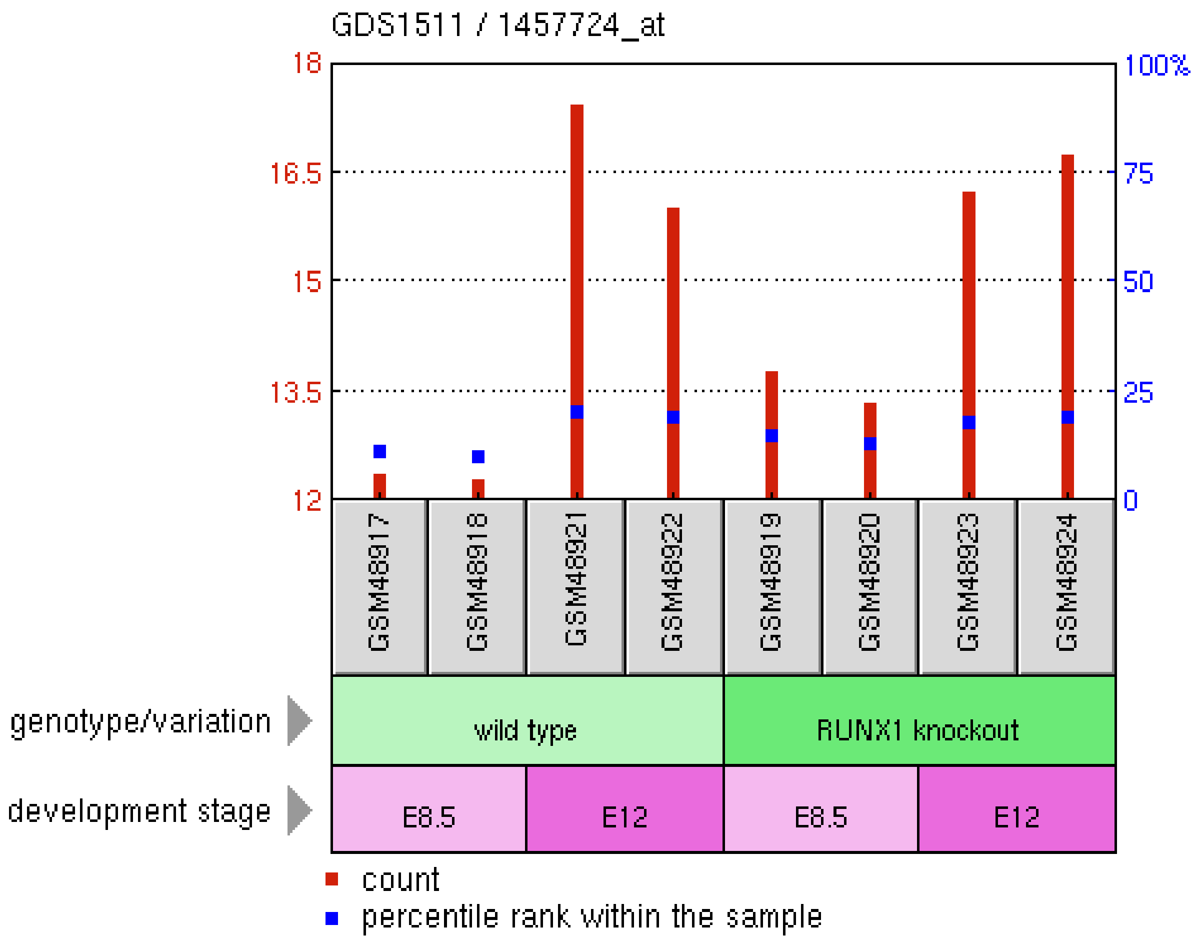

Sample	Title	Value
**GSM48917**	E8.5 wt.1	12.3917
**GSM48918**	E8.5 wt.2	12.3148
**GSM48921**	E12 wt.1	17.4507
**GSM48922**	E12 wt.2	16.0288
**GSM48919**	E8.5 ko.1	13.783
**GSM48920**	E8.5 ko.2	13.3608
**GSM48923**	E12 ko.1	16.2344
**GSM48924**	E12 ko.2	16.7351

Supplementary Figure 9. Possible mechanisms affecting gene expression of TMPRSS2 and CTSB/L. Mostly different transcription factors affect expression of the target genes.

TF Perturbations Followed by Expression

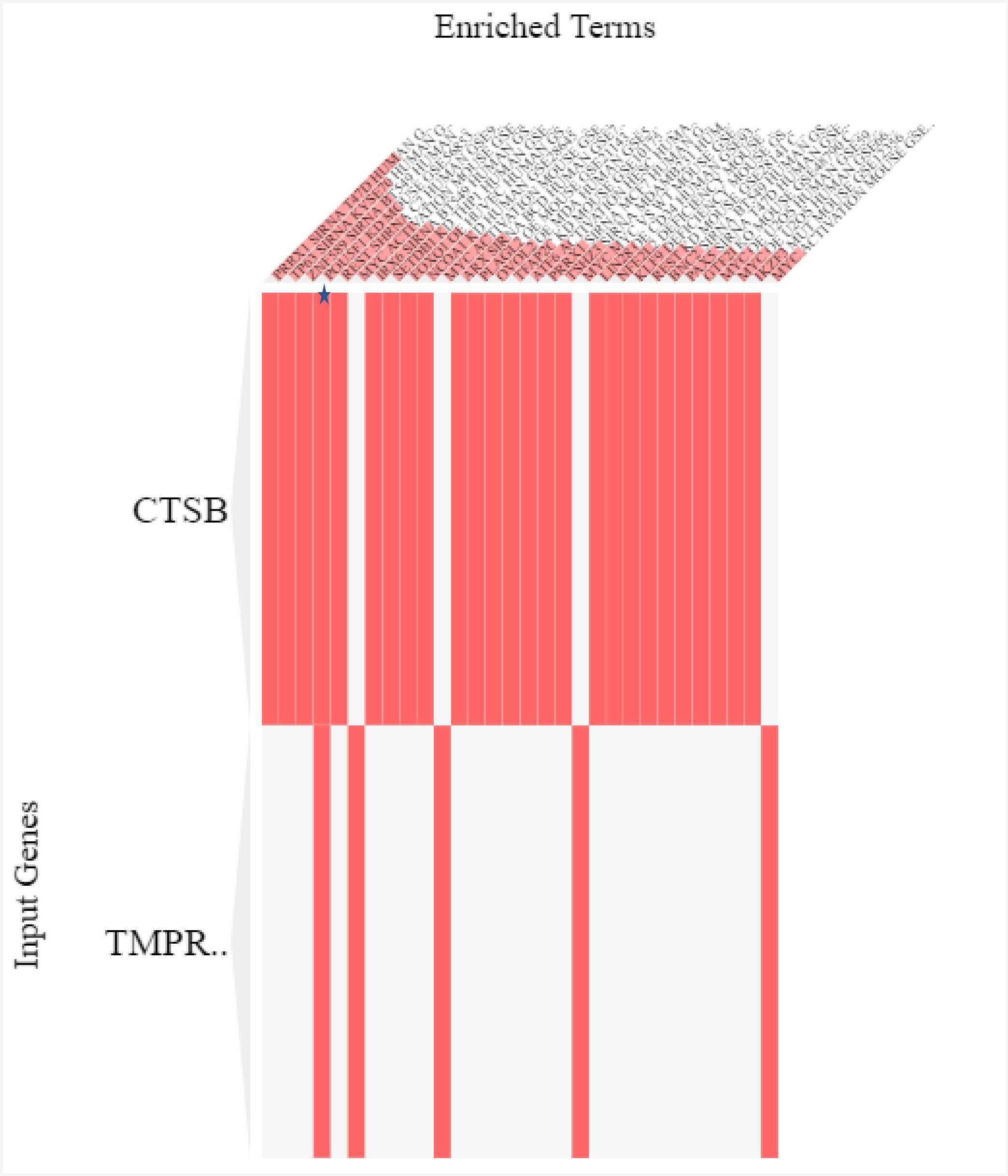

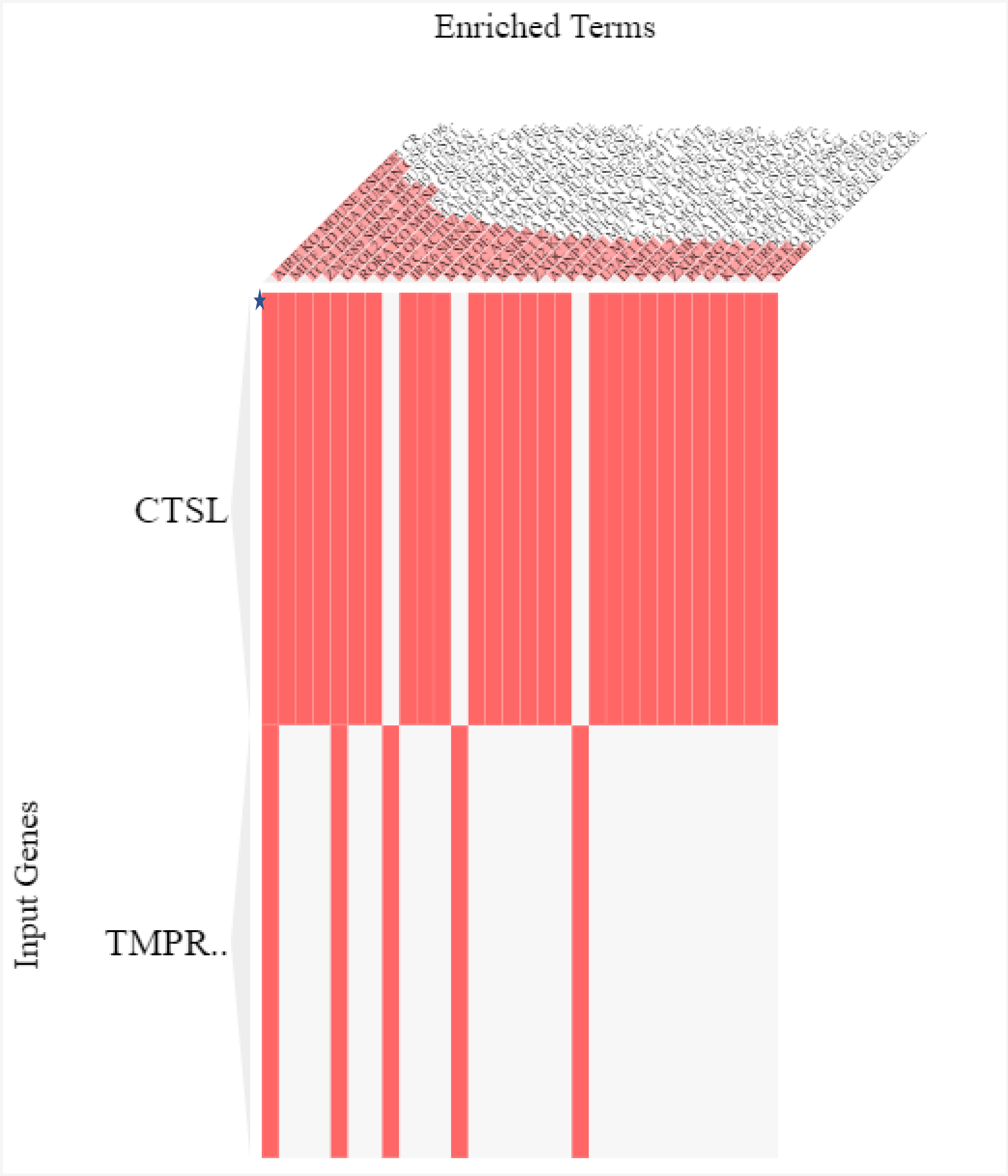

***POU5F1* and *AIRE* gene product as potential repressor of the target gene expression**

Gene Perturbations from GEO up

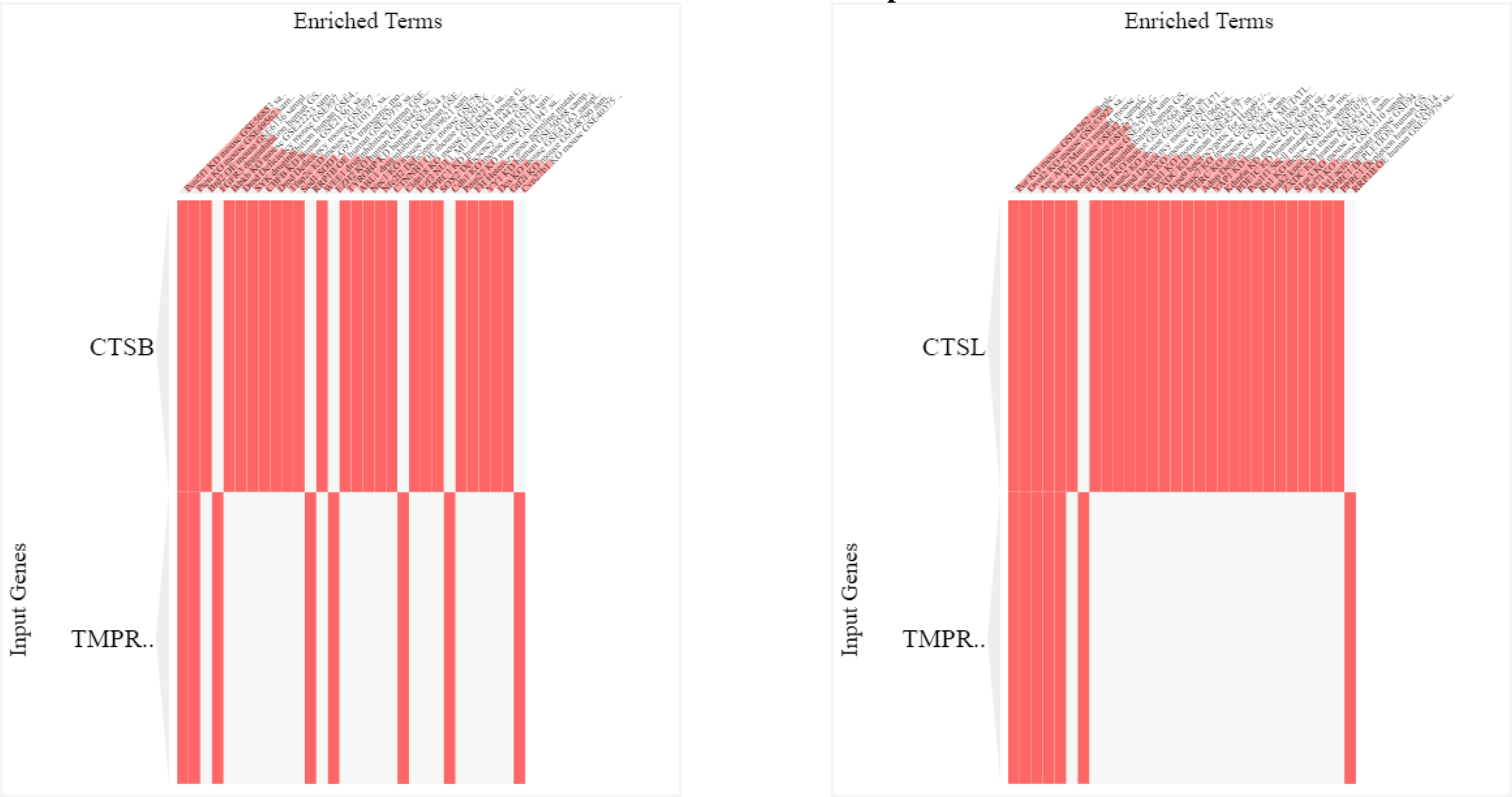

***POU5F1* and *AIRE* gene product as potential repressor of the target gene expression**

Supplementary Figure 10. Possible mechanisms affecting gene expression of TMPRSS2 and CTSB/L. Identification of enriched GEO records revealed that transcription factors POU5F1 and AIRE downregulates the expression of the target genes.

Tmprss2 - Transcription factors Nanog and Oct4 knockdown effect on embryonic stem cells

**Profile**	GDS1824 / 1449369_at
**Title**	Transcription factors Nanog and Oct4 knockdown effect on embryonic stem cells
**Organism**	Mus musculus

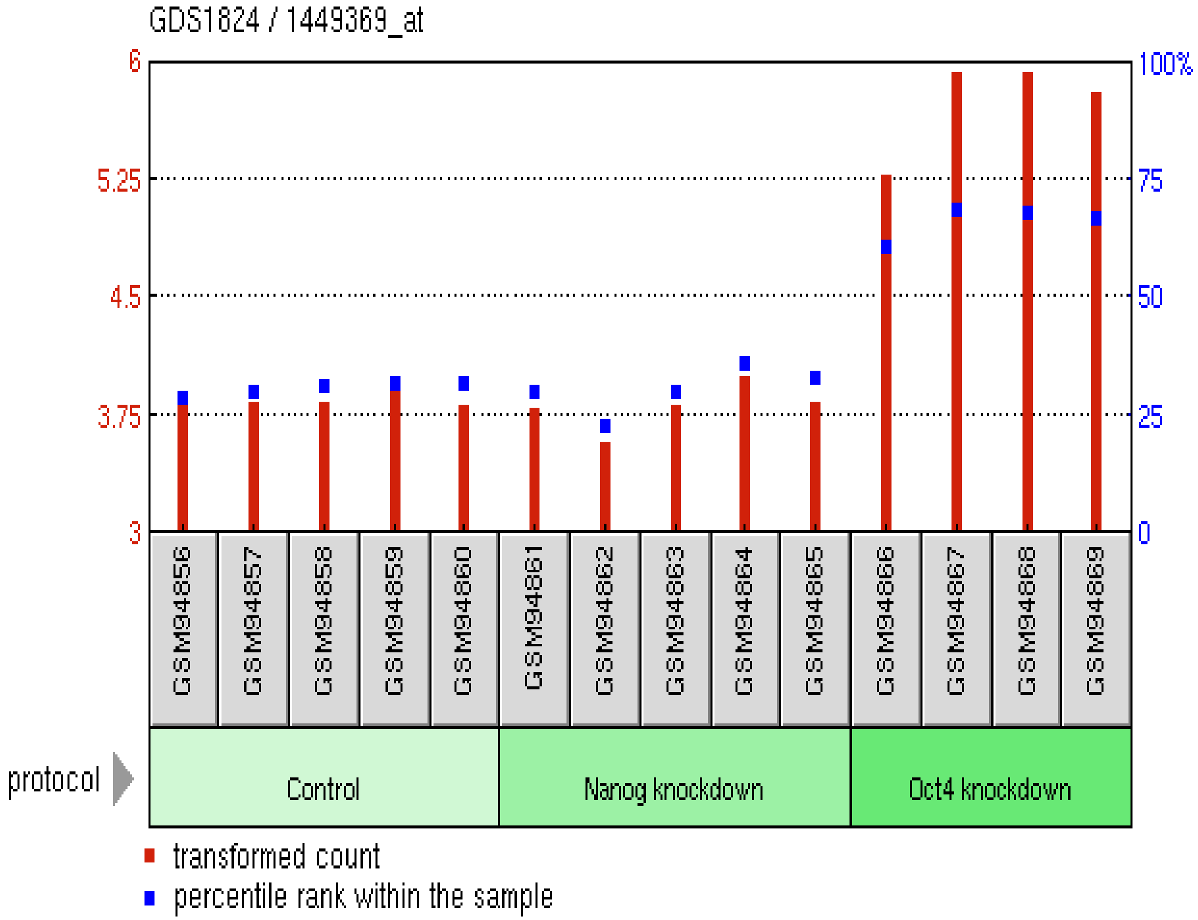

Sample	Title	Value
GSM94856	Parental vector, pSUPER-puro replicate 1	3.81676
GSM94857	Parental vector, pSUPER-puro replicate 2	3.83908
GSM94858	Parental vector, pSUPER-puro replicate 3	3.8432
GSM94859	Parental vector, pSUPER-puro replicate 4	3.91131
GSM94860	Parental vector, pSUPER-puro replicate 5	3.82767
GSM94861	Nanog RNAi replicate-1	3.8035
GSM94862	Nanog RNAi replicate-2	3.59369
GSM94863	Nanog RNAi replicate-3	3.81672
GSM94864	Nanog RNAi replicate-4	4.00128
GSM94865	Nanog RNAi replicate-5	3.84154
GSM94866	Pou5f1 RNAi replicate-1	5.28393
GSM94867	Pou5f1 RNAi replicate-2	5.94477
GSM94868	Pou5f1 RNAi replicate-3	5.93279
GSM94869	Pou5f1 RNAi replicate-4	5.80137

Ctsb - Transcription factors Nanog and Oct4 knockdown effect on embryonic stem cells

**Profile**	GDS1824 / 1448732_at
**Title**	Transcription factors Nanog and Oct4 knockdown effect on embryonic stem cells
**Organism**	Mus musculus

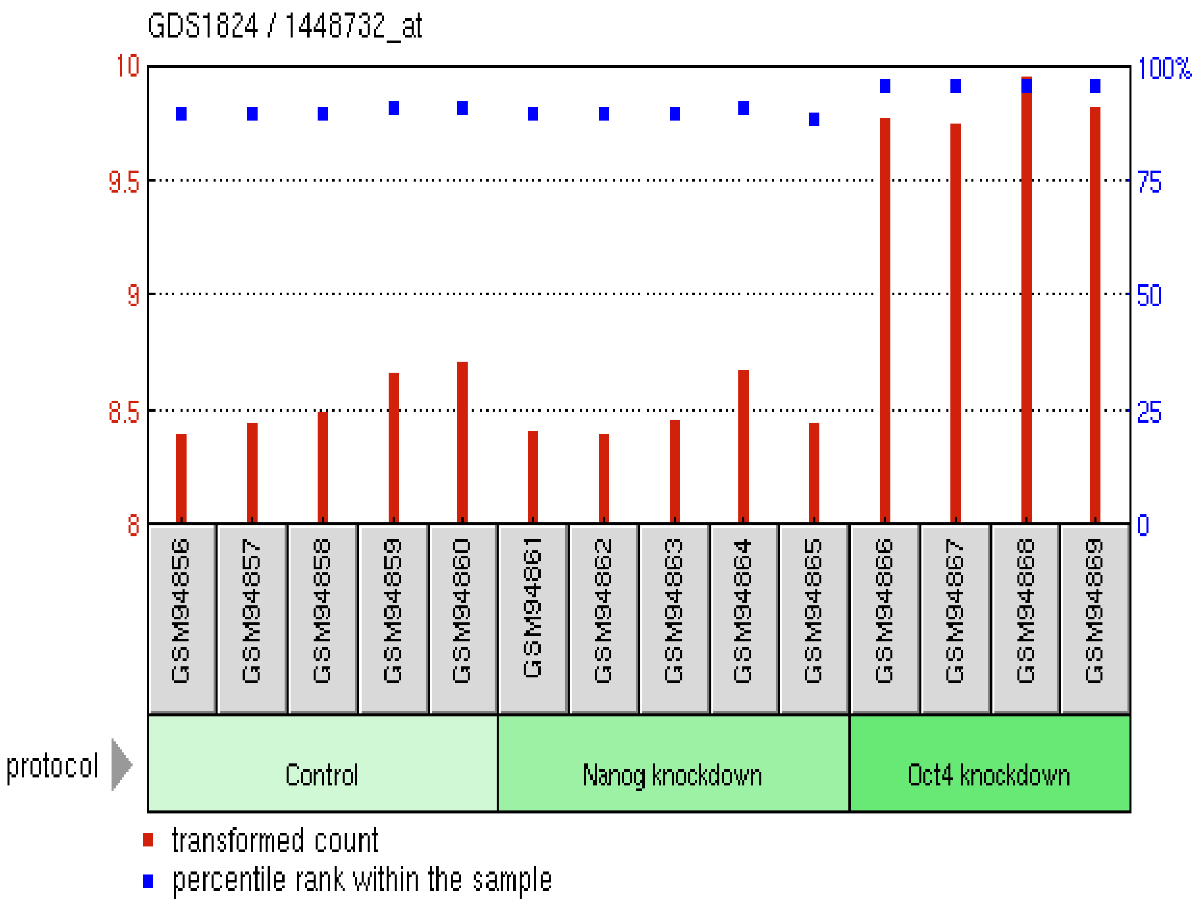

Sample	Title	Value
GSM94856	Parental vector, pSUPER-puro replicate 1	8.39775
GSM94857	Parental vector, pSUPER-puro replicate 2	8.45482
GSM94858	Parental vector, pSUPER-puro replicate 3	8.49881
GSM94859	Parental vector, pSUPER-puro replicate 4	8.67404
GSM94860	Parental vector, pSUPER-puro replicate 5	8.71712
GSM94861	Nanog RNAi replicate-1	8.41842
GSM94862	Nanog RNAi replicate-2	8.40876
GSM94863	Nanog RNAi replicate-3	8.45788
GSM94864	Nanog RNAi replicate-4	8.68011
GSM94865	Nanog RNAi replicate-5	8.45129
GSM94866	Pou5f1 RNAi replicate-1	9.77969
GSM94867	Pou5f1 RNAi replicate-2	9.74865
GSM94868	Pou5f1 RNAi replicate-3	9.95266
GSM94869	Pou5f1 RNAi replicate-4	9.82503

Ctsl - Transcription factors Nanog and Oct4 knockdown effect on embryonic stem cells

**Profile**	GDS1824 / 1451310_a_at
**Title**	Transcription factors Nanog and Oct4 knockdown effect on embryonic stem cells
**Organism**	Mus musculus

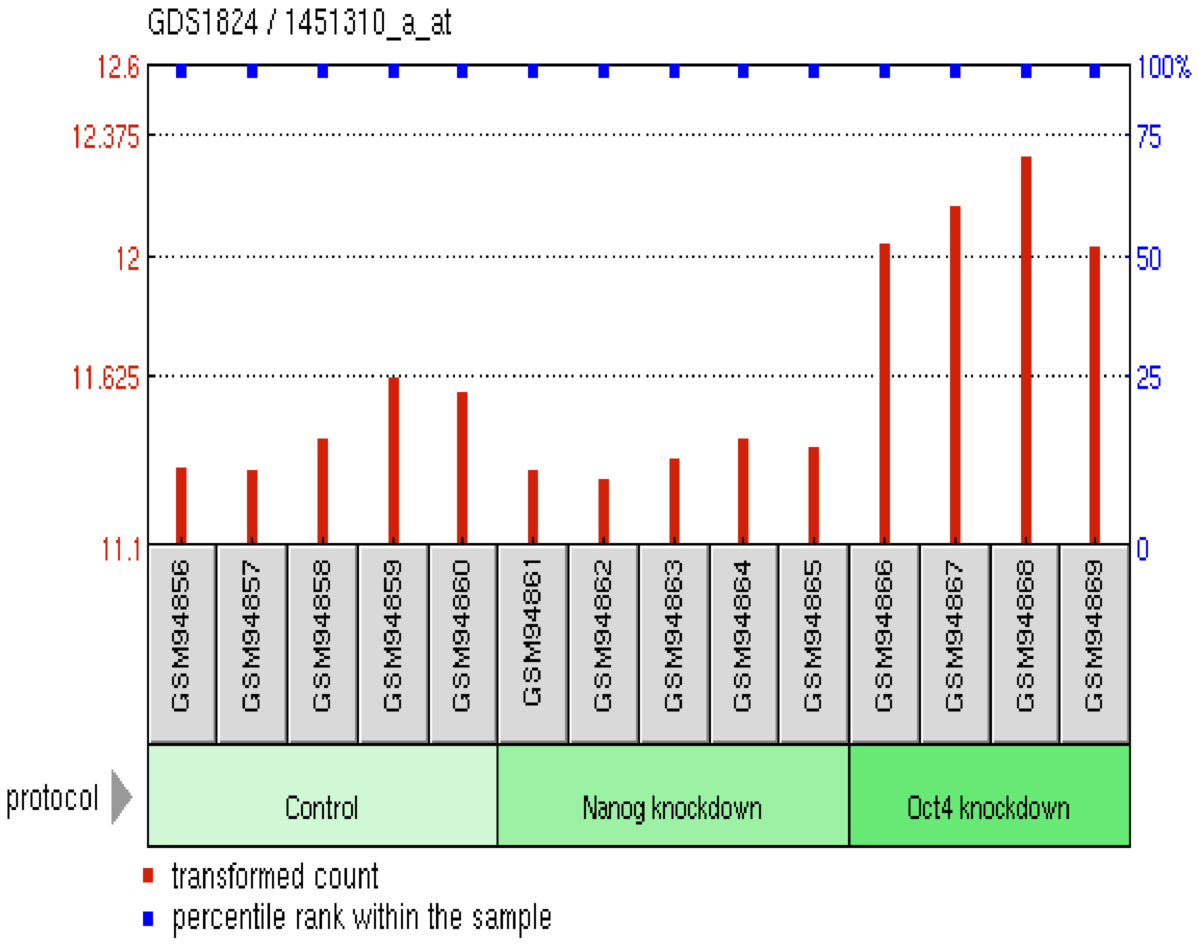

Sample	Title	Value
GSM94856	Parental vector, pSUPER-puro replicate 1	11.3468
GSM94857	Parental vector, pSUPER-puro replicate 2	11.3353
GSM94858	Parental vector, pSUPER-puro replicate 3	11.4391
GSM94859	Parental vector, pSUPER-puro replicate 4	11.6296
GSM94860	Parental vector, pSUPER-puro replicate 5	11.58
GSM94861	Nanog RNAi replicate-1	11.3356
GSM94862	Nanog RNAi replicate-2	11.3137
GSM94863	Nanog RNAi replicate-3	11.3768
GSM94864	Nanog RNAi replicate-4	11.4356
GSM94865	Nanog RNAi replicate-5	11.4116
GSM94866	Pou5f1 RNAi replicate-1	12.043
GSM94867	Pou5f1 RNAi replicate-2	12.159
GSM94868	Pou5f1 RNAi replicate-3	12.3137
GSM94869	Pou5f1 RNAi replicate-4	12.0377

Tmprss2 - Autoimmune regulator AIRE knockout effect on thymic epithelial cells

**Profile**	GDS2015 / 1419154_at
**Title**	Autoimmune regulator AIRE knockout effect on thymic epithelial cells
**Organism**	Mus musculus

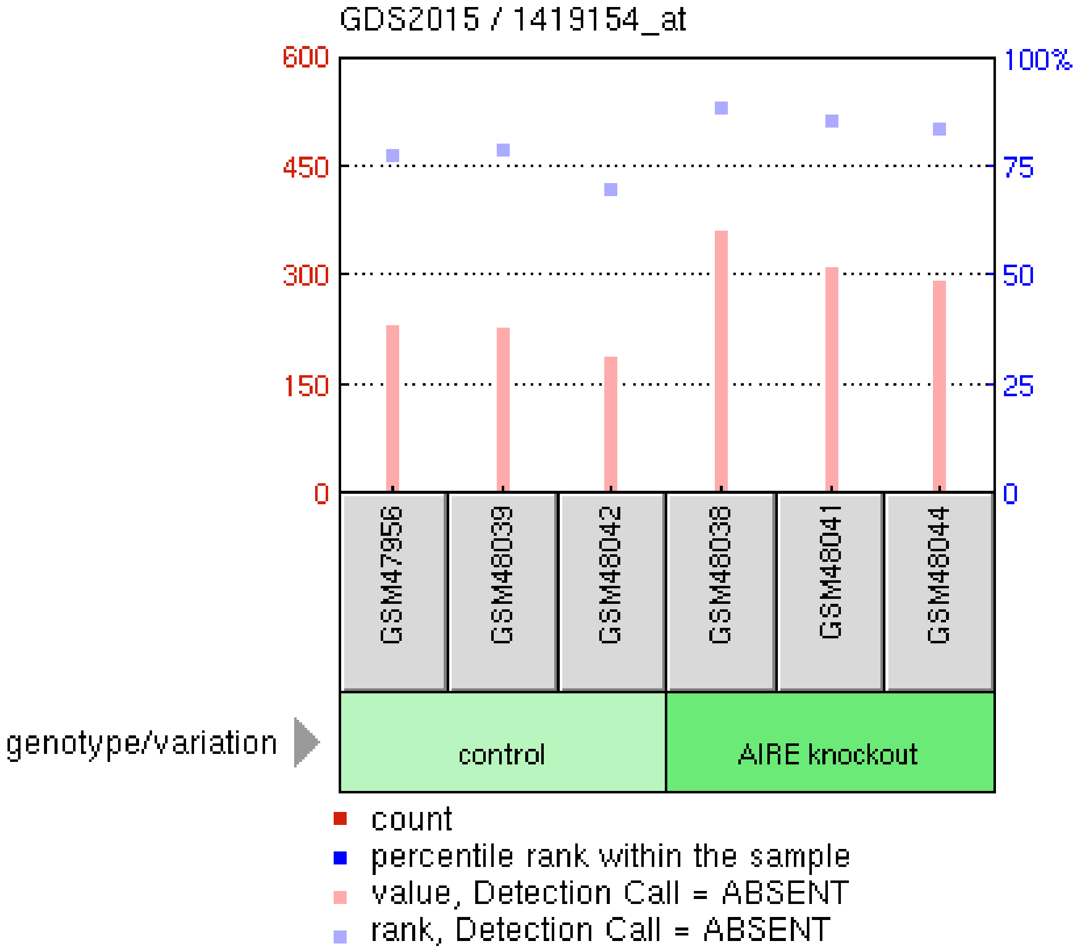

Sample	Title	Value
GSM47956	566.B6	232.1
GSM48039	568.B6	229.7
GSM48042	570.B6	189.2
GSM48038	567.KO	363.2
GSM48041	569.KO	313.2
GSM48044	571.KO	295.9

Ctsb - Autoimmune regulator AIRE knockout effect on thymic epithelial cells

Profile GDS2015 / 1448732_at

Title Autoimmune regulator AIRE knockout effect on thymic epithelial cells

Organism Mus musculus

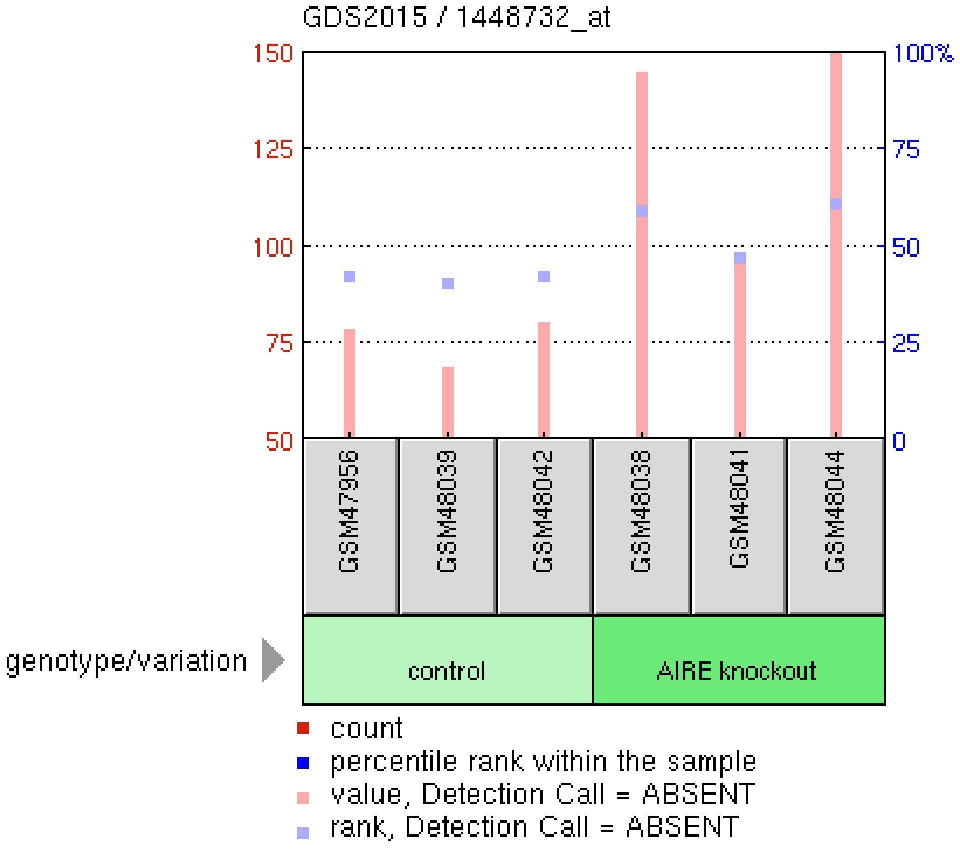

Sample	Title	Value
GSM47956	566.B6	78.7
GSM48039	568.B6	68.7
GSM48042	570.B6	80.6
GSM48038	567.KO	145
GSM48041	569.KO	97.6
GSM48044	571.KO	149.6

Ctsl - Autoimmune regulator AIRE knockout effect on thymic epithelial cells

Profile GDS2015 / 1451310_a_at

Title Autoimmune regulator AIRE knockout effect on thymic epithelial cells

Organism Mus musculus

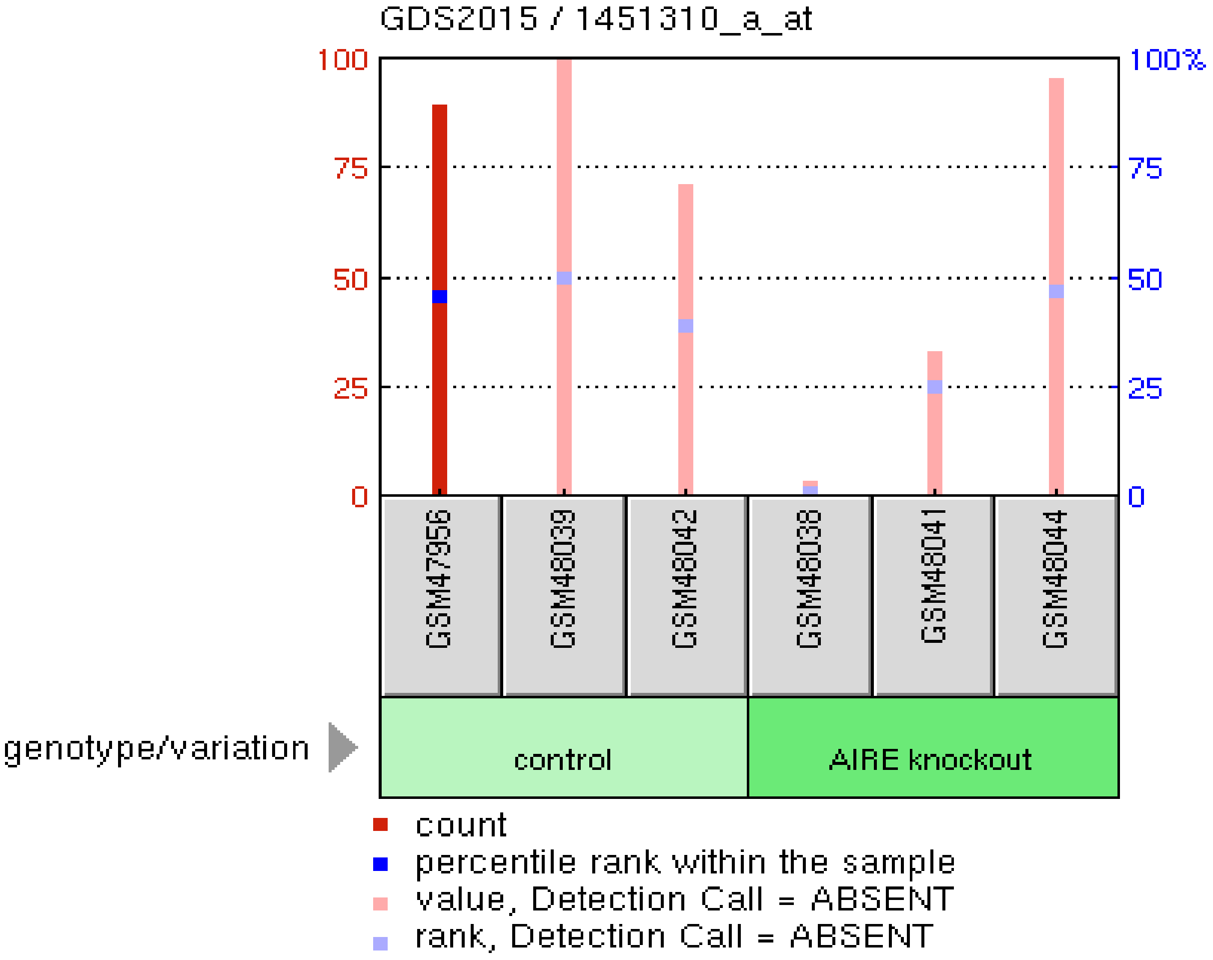

Sample	Title	Value
GSM47956	566.B6	89.6
GSM48039	568.B6	99.9
GSM48042	570.B6	71.5
GSM48038	567.KO	4.2
GSM48041	569.KO	33.7
GSM48044	571.KO	95.6

Supplementary Figure 11. GSEA identify Estradiol and Retinoic acid as potential drug candidate against SARS-CoV-2 infection.

Estradiol down-regulating expression of the *TMPRSS2 and CTSB/L* genes

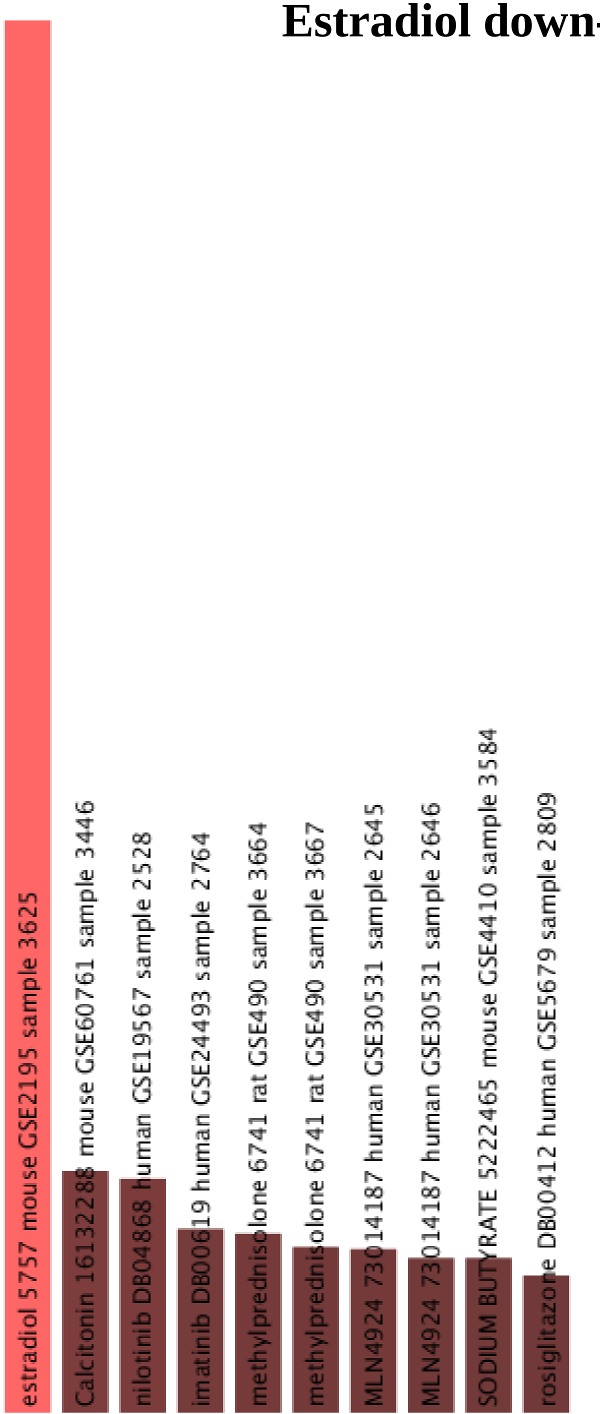

Drug Perturbations from GEO down

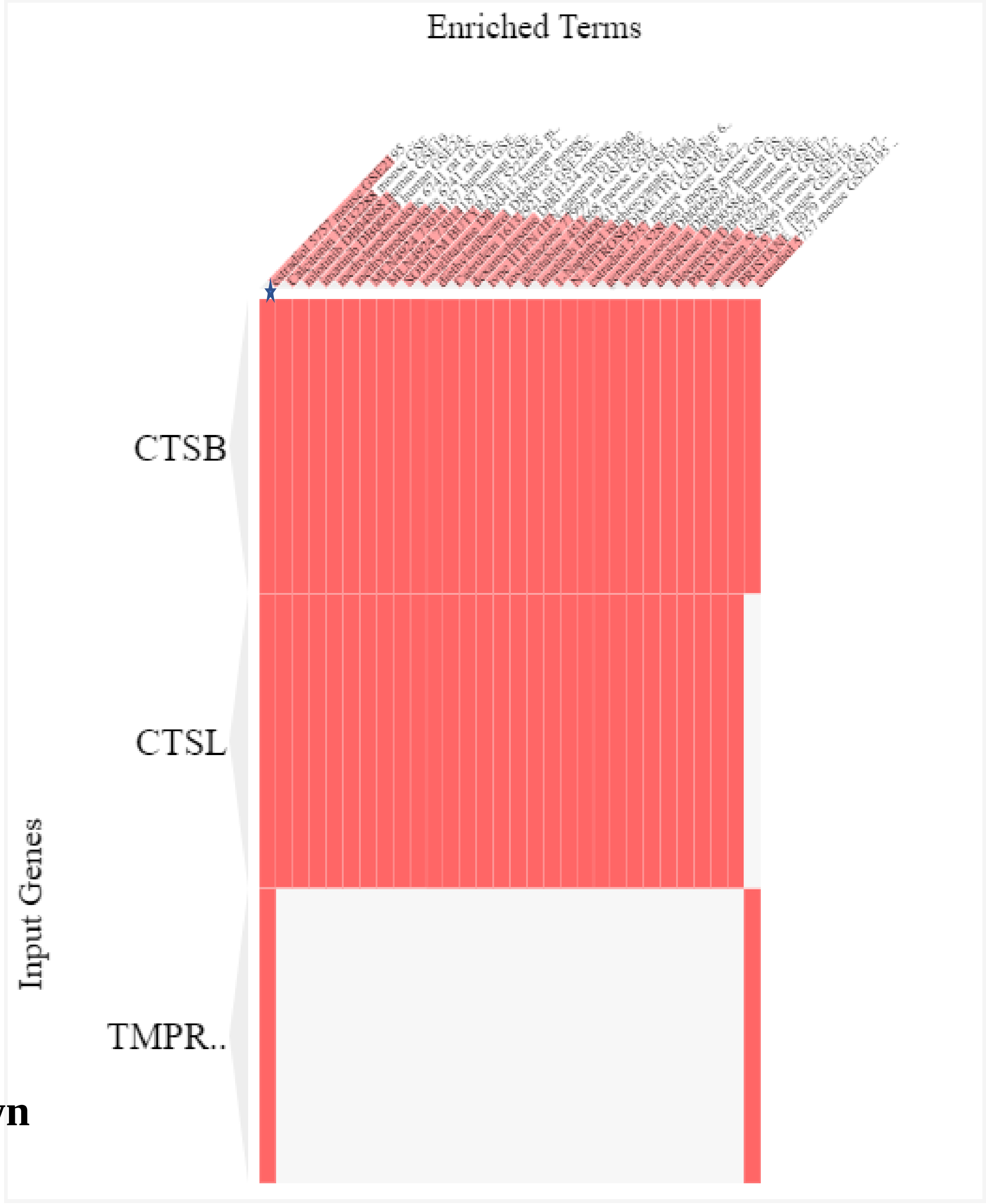

Different ligands down-regulating expression of the *TMPRSS2 and CTSB/L* genes

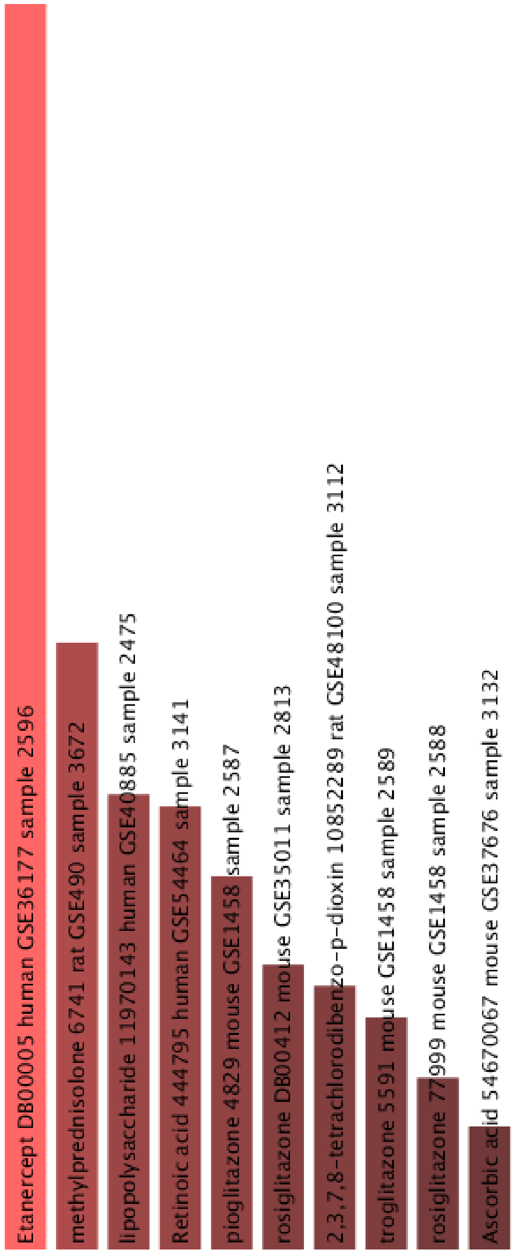

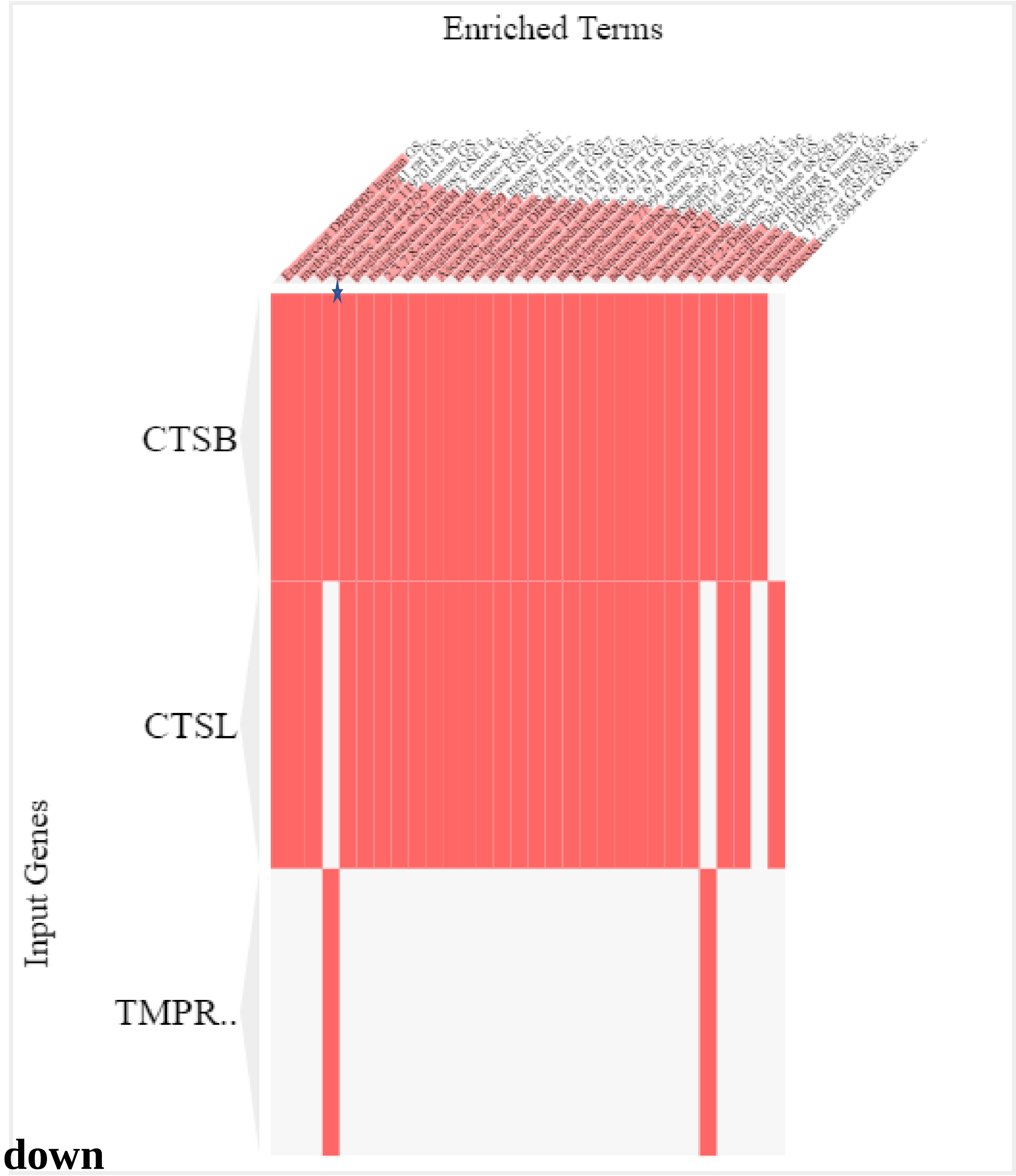

Ligand Perturbations from GEO down

Supplementary Figure 12. Estradiol and Retinoic acid as potential drug candidate mitigating COVID-19 infection. Both estradiol and retinoic acid inhibit TMPRSS2 and CTSB/L genes as shown by enriched GEO records.

TMPRSS2 - Endothelial cell response to estradiol in vitro

**Profile**	GDS3600 / 226553_at
**Title**	Endothelial cell response to estradiol in vitro
**Organism**	Homo sapiens

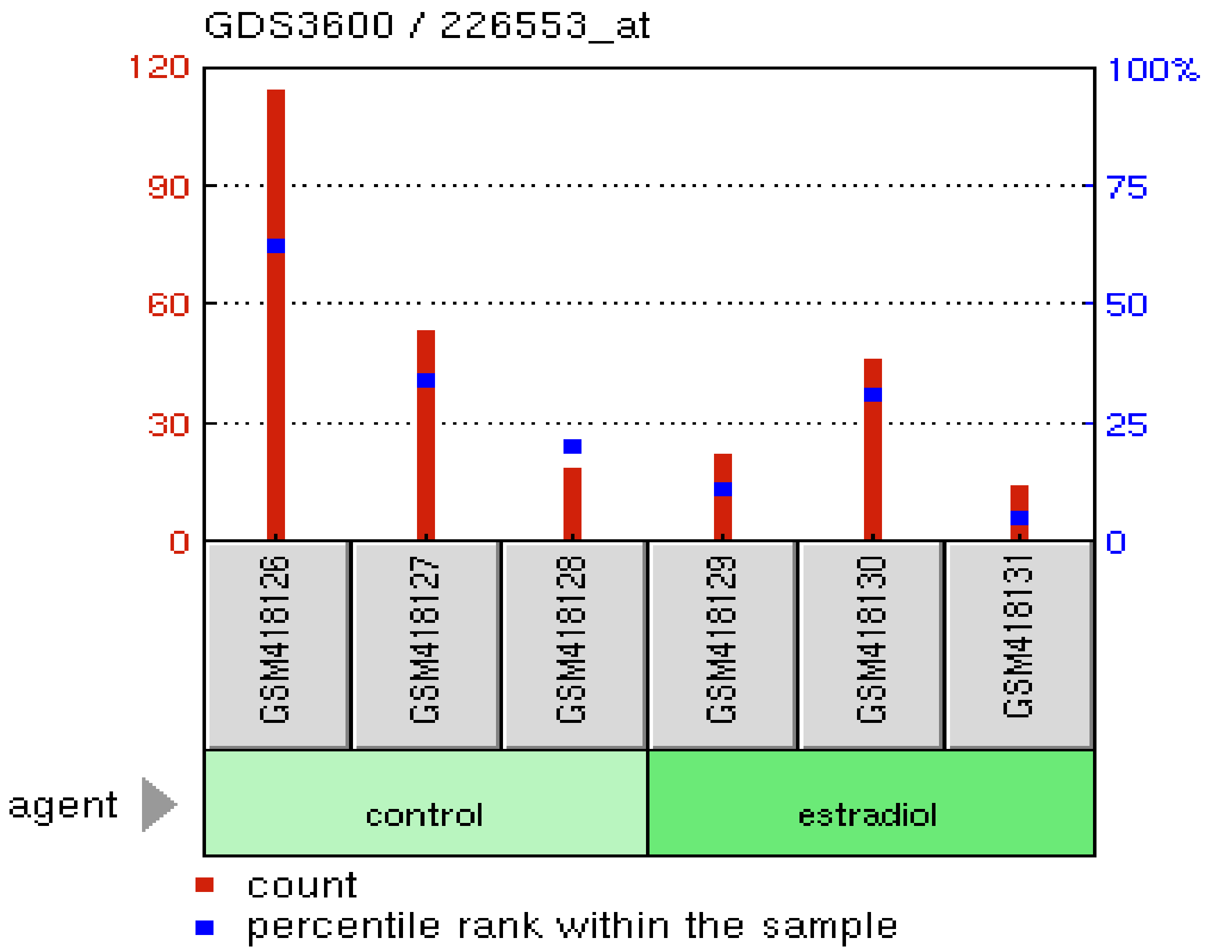

Sample	Title	Value
GSM418126	Control, rep 1	114.23
GSM418127	Control, rep 2	53.63
GSM418128	Control, rep 3	18.87
GSM418129	Estradiol, rep 1	22.92
GSM418130	Estradiol, rep 2	46.92
GSM418131	Estradiol, rep 3	15.16

CTSB - Endothelial cell response to estradiol in vitro

**Profile**	GDS3600 / 227961_at
**Title**	Endothelial cell response to estradiol in vitro
**Organism**	Homo sapiens

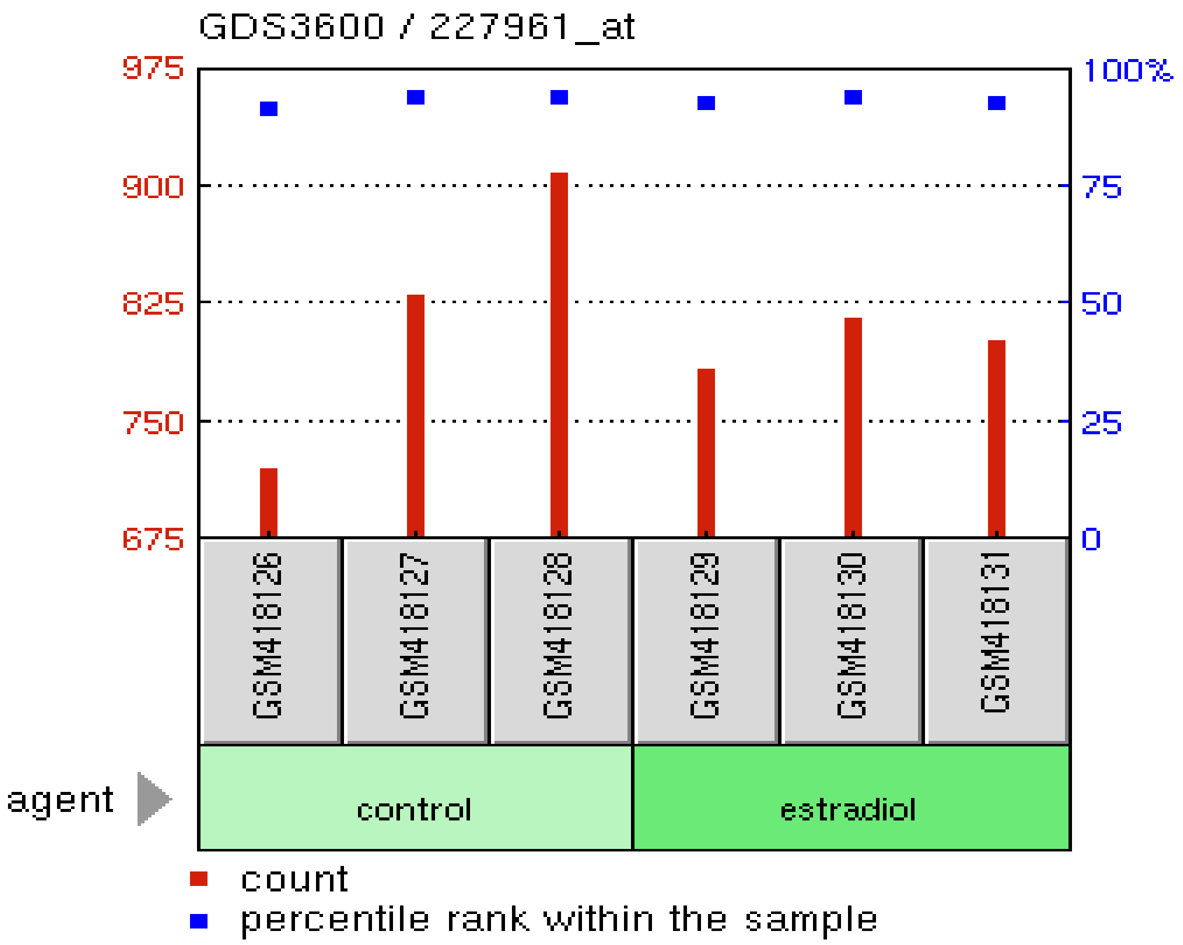

Sample	Title	Value
GSM418126	Control, rep 1	721.23
GSM418127	Control, rep 2	831.93
GSM418128	Control, rep 3	909.18
GSM418129	Estradiol, rep 1	783.53
GSM418130	Estradiol, rep 2	817.08
GSM418131	Estradiol, rep 3	801.53

CTSL - Endothelial cell response to estradiol in vitro

**Profile**	GDS3600 / 202087_s_at
**Title**	Endothelial cell response to estradiol in vitro
**Organism**	Homo sapiens

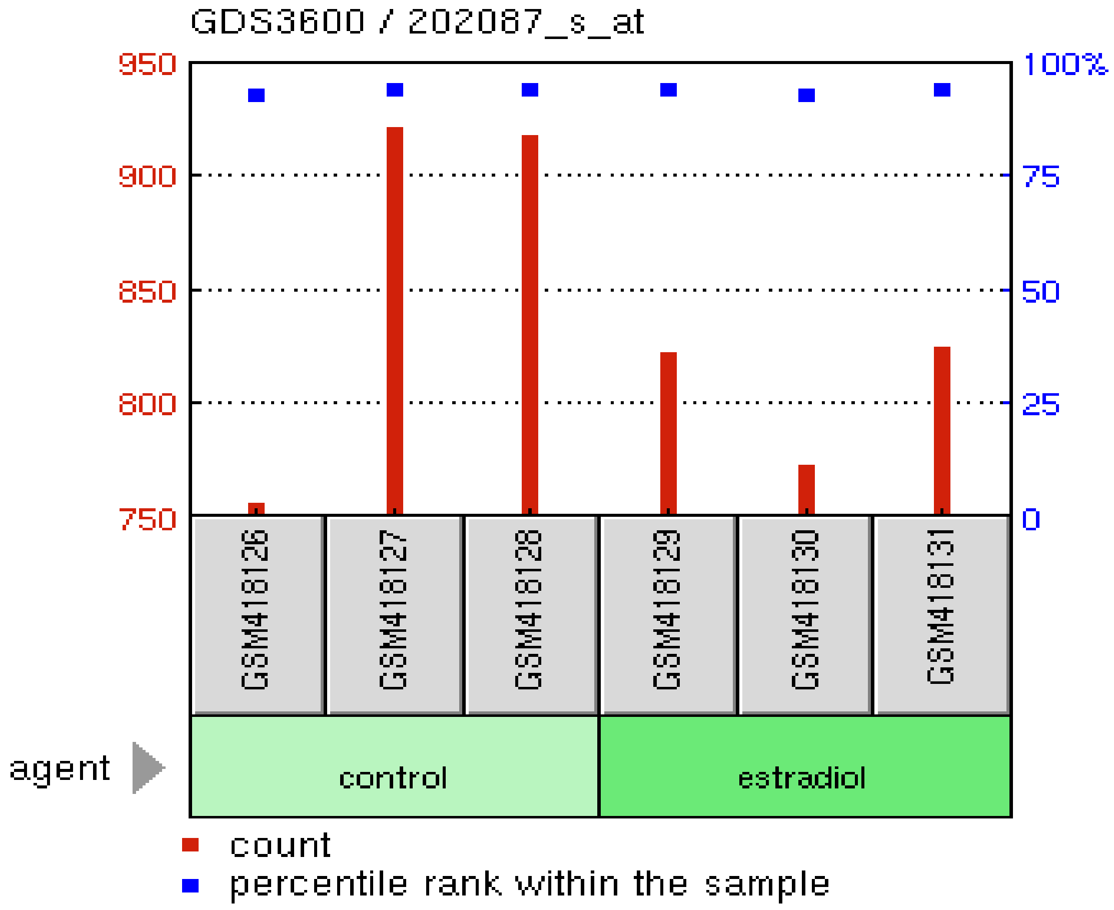

Sample	Title	Value
GSM418126	Control, rep 1	756.37
GSM418127	Control, rep 2	921.5
GSM418128	Control, rep 3	918.6
GSM418129	Estradiol, rep 1	822.67
GSM418130	Estradiol, rep 2	774.05
GSM418131	Estradiol, rep 3	824.86

TMPRSS2 - All-trans retinoic acid (ATRA) vitamin A and 1,25-dihydroxyvitamin D3 (1,25D3) vitamin D stimulated peripheral blood monocytes

**Profile**	GDS4860 / 205102_at
**Title**	All-trans retinoic acid (ATRA) vitamin A and 1,25-dihydroxyvitamin D3 (1,25D3) vitamin D stimulated peripheral blood monocytes
**Organism**	Homo sapiens

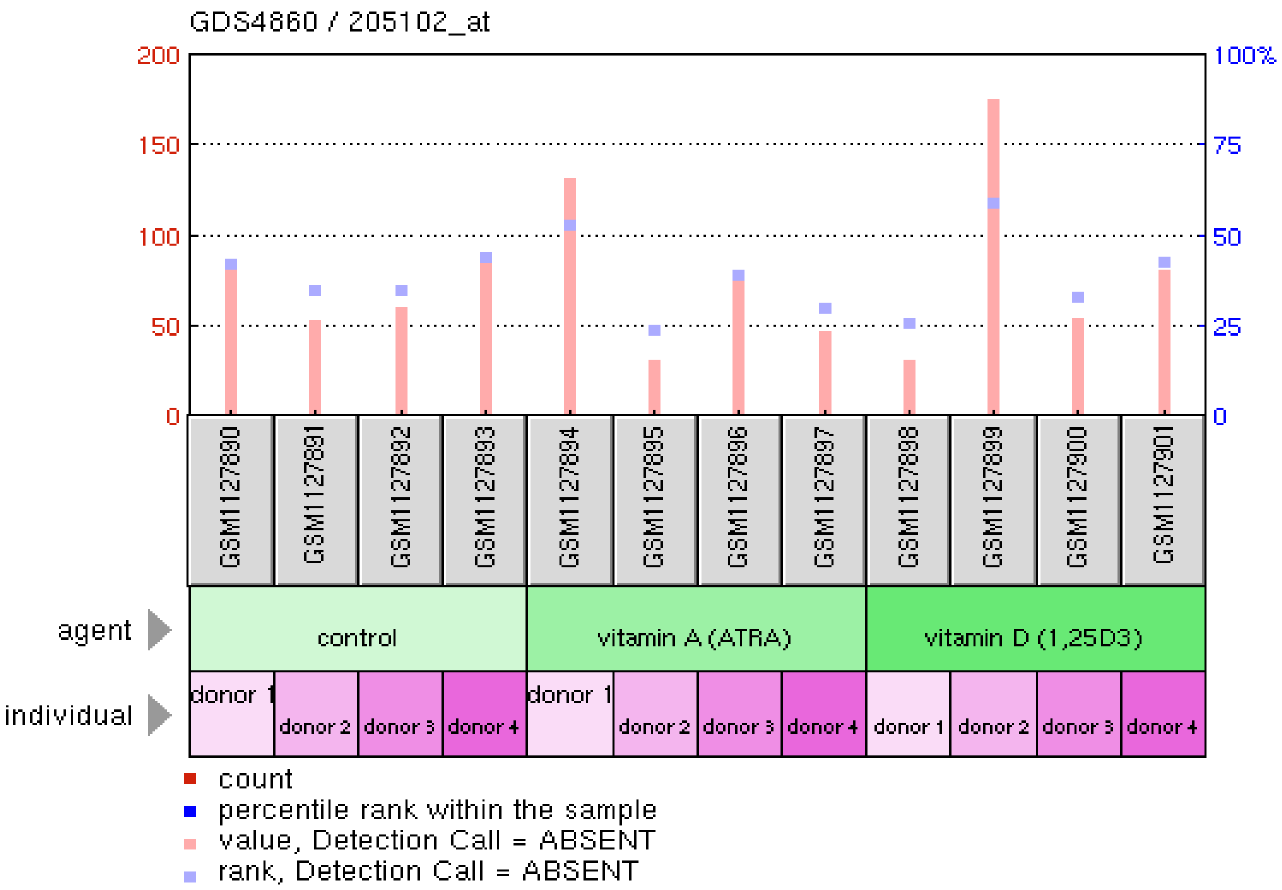

Sample	Title	Value
GSM1127890	monocyte from donor 1 stimulated with CTRL	87.4565
GSM1127891	monocyte from donor 2 stimulated with CTRL	53.2887
GSM1127892	monocyte from donor 3 stimulated with CTRL	61.3627
GSM1127893	monocyte from donor 4 stimulated with CTRL	90.4528
GSM1127894	monocyte from donor 1 stimulated with ATRA	132.264
GSM1127895	monocyte from donor 2 stimulated with ATRA	31.4246
GSM1127896	monocyte from donor 3 stimulated with ATRA	80.7065
GSM1127897	monocyte from donor 4 stimulated with ATRA	47.3273
GSM1127898	monocyte from donor 1 stimulated with 1,25D	32.2077
GSM1127899	monocyte from donor 2 stimulated with 1,25D	175.044
GSM1127900	monocyte from donor 3 stimulated with 1,25D	54.7452
GSM1127901	monocyte from donor 4 stimulated with 1,25D	80.8336

CTSB - All-trans retinoic acid (ATRA) vitamin A and 1,25-dihydroxyvitamin D3 (1,25D3) vitamin D stimulated peripheral blood monocytes

**Profile**	GDS4860 / 213275_x_at
**Title**	All-trans retinoic acid (ATRA) vitamin A and 1,25-dihydroxyvitamin D3 (1,25D3) vitamin D stimulated peripheral blood monocytes
**Organism**	Homo sapiens

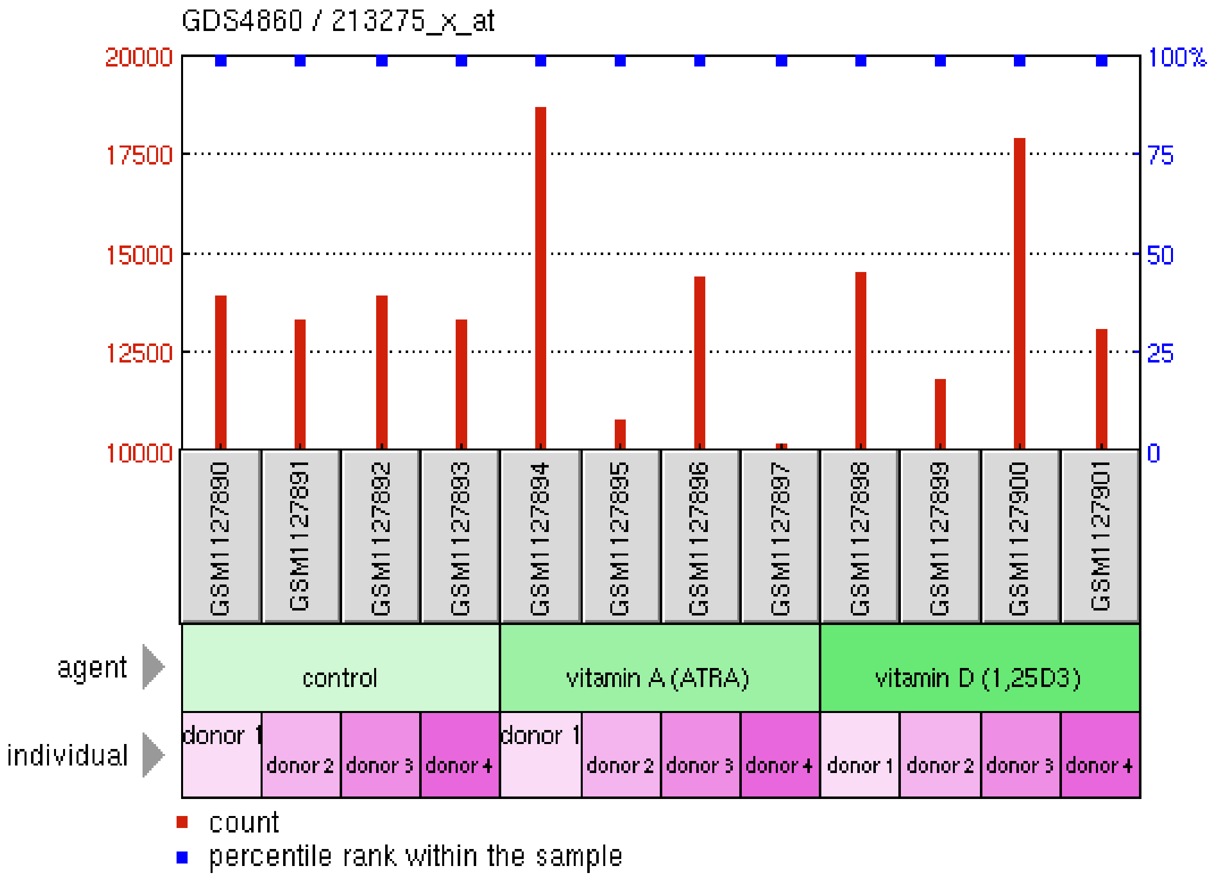

Sample	Title	Value
GSM1127890	monocyte from donor 1 stimulated with CTRL	13965.2
GSM1127891	monocyte from donor 2 stimulated with CTRL	13343.6
GSM1127892	monocyte from donor 3 stimulated with CTRL	13961.5
GSM1127893	monocyte from donor 4 stimulated with CTRL	13361.3
GSM1127894	monocyte from donor 1 stimulated with ATRA	18713.4
GSM1127895	monocyte from donor 2 stimulated with ATRA	10843.2
GSM1127896	monocyte from donor 3 stimulated with ATRA	14423.7
GSM1127897	monocyte from donor 4 stimulated with ATRA	10010.1
GSM1127898	monocyte from donor 1 stimulated with 1,25D	14567.6
GSM1127899	monocyte from donor 2 stimulated with 1,25D	11855.5
GSM1127900	monocyte from donor 3 stimulated with 1,25D	17895.4
GSM1127901	monocyte from donor 4 stimulated with 1,25D	13076.8

CTSL - All-trans retinoic acid (ATRA) vitamin A and 1,25-dihydroxyvitamin D3 (1,25D3) vitamin D stimulated peripheral blood monocytes

**Profile**	GDS4860 / 202087_s_at
**Title**	All-trans retinoic acid (ATRA) vitamin A and 1,25-dihydroxyvitamin D3 (1,25D3) vitamin D stimulated peripheral blood monocytes
**Organism**	Homo sapiens

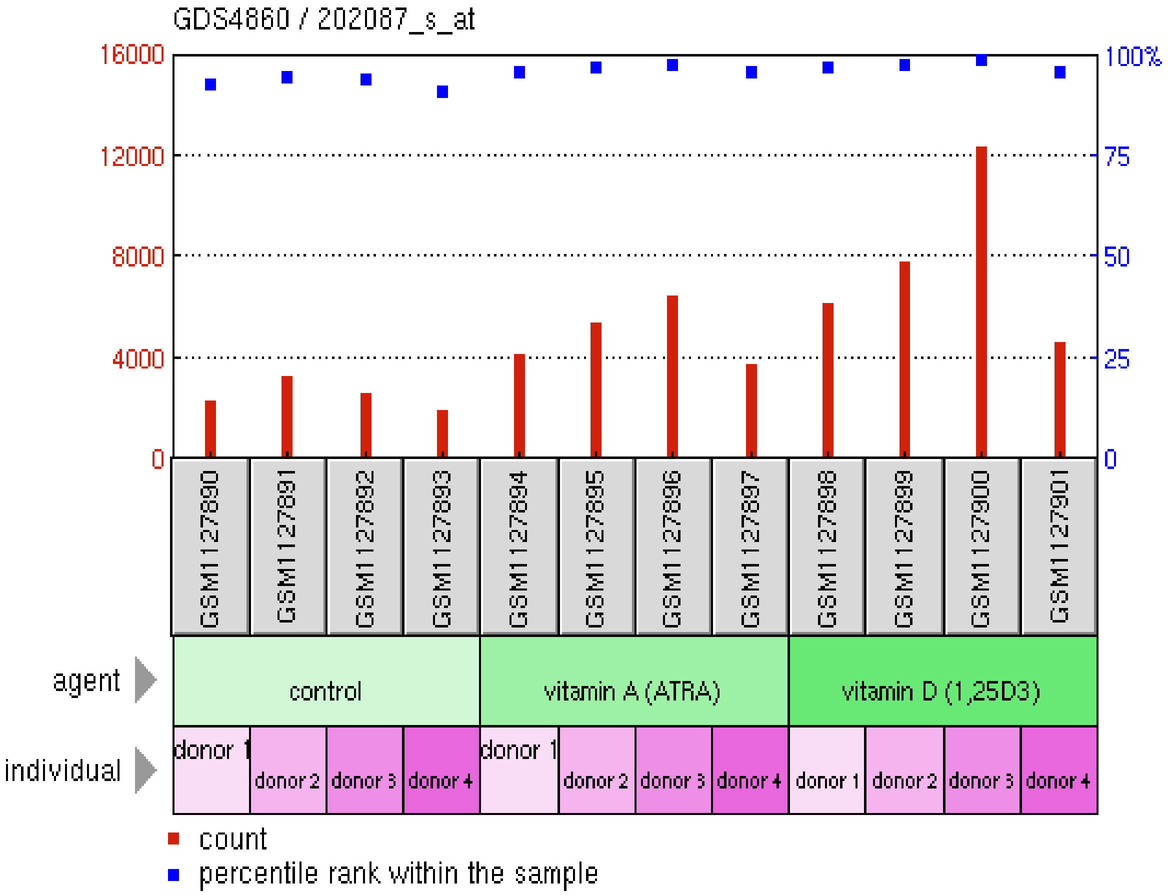

Sample	Title	Value
GSM1127890	monocyte from donor 1 stimulated with CTRL	2387.19
GSM1127891	monocyte from donor 2 stimulated with CTRL	3335.13
GSM1127892	monocyte from donor 3 stimulated with CTRL	2650.46
GSM1127893	monocyte from donor 4 stimulated with CTRL	1964.65
GSM1127894	monocyte from donor 1 stimulated with ATRA	4214.74
GSM1127895	monocyte from donor 2 stimulated with ATRA	5474.47
GSM1127896	monocyte from donor 3 stimulated with ATRA	6471.42
GSM1127897	monocyte from donor 4 stimulated with ATRA	3761.98
GSM1127898	monocyte from donor 1 stimulated with 1,25D	6190.2
GSM1127899	monocyte from donor 2 stimulated with 1,25D	7821.26
GSM1127900	monocyte from donor 3 stimulated with 1,25D	12345.9
GSM1127901	monocyte from donor 4 stimulated with 1,25D	4659.58

Supplementary Figure 13. Effect of Estradiol and Retinoic acid as putative modulators of transcription-factor binding sites of target genes, TMPRSS2 and CTSB/L. Estradiol upregulates s the expression of PPARγ, and PPARα, while downregulates the expression of SOX17 and RUNX1 genes. All-trans retinoic acid increased the expression of PPARγ and PPARα, while decreased the expression of SOX17 in human peripheral blood monocytes.

PPARG - Endothelial cell response to estradiol in vitro

**Profile**	GDS3600 / 208510_s_at
**Title**	Endothelial cell response to estradiol in vitro
**Organism**	Homo sapiens

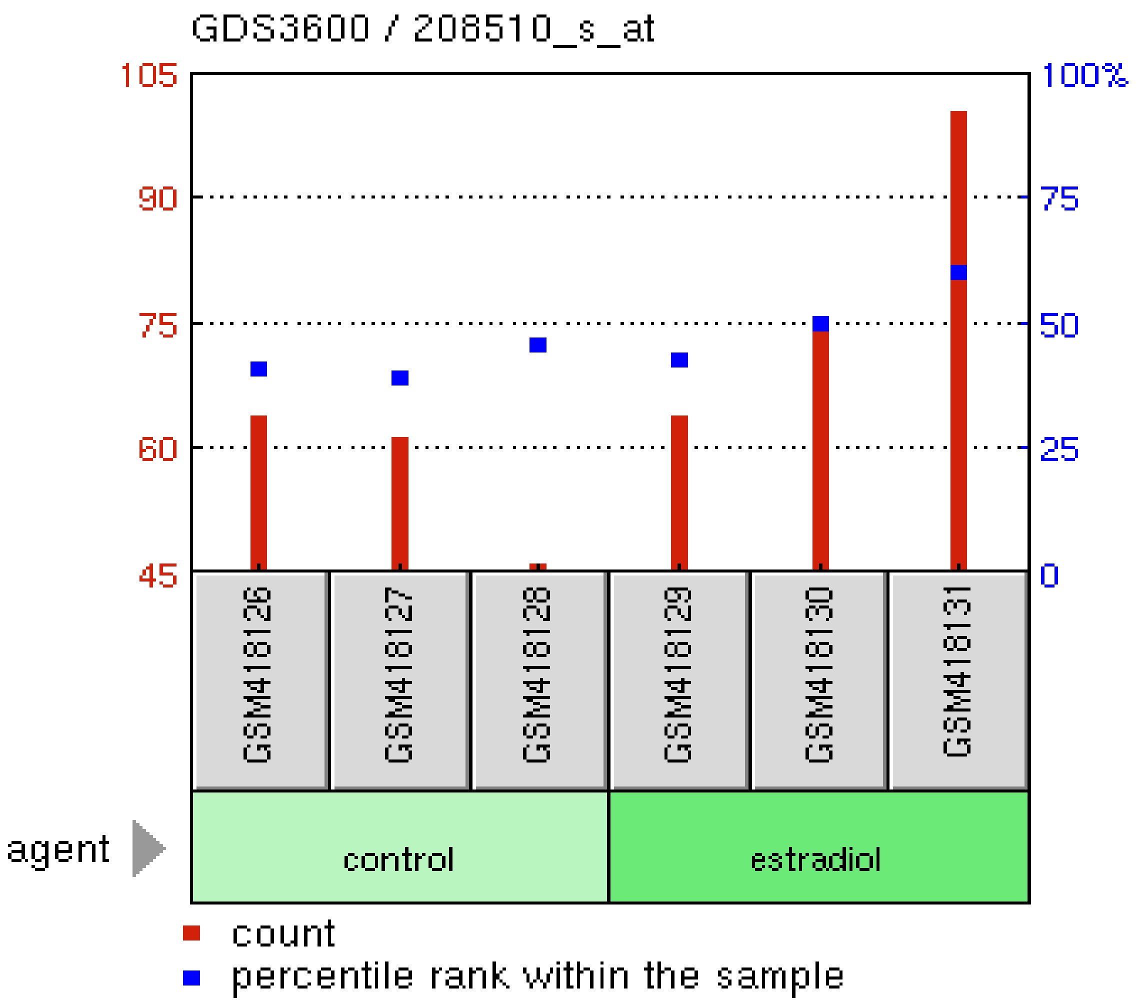

Sample	Title	Value
GSM418126	Control, rep 1	63.87
GSM418127	Control, rep 2	61.3
GSM418128	Control, rep 3	45.28
GSM418129	Estradiol, rep 1	64.15
GSM418130	Estradiol, rep 2	75.86
GSM418131	Estradiol, rep 3	100.46

[PPARG - Estradiol effect on breast cancer cell line: time course]

**Profile**	GDS3283 / 208510_s_at
**Title**	Estradiol effect on breast cancer cell line: time course
**Organism**	Homo sapiens

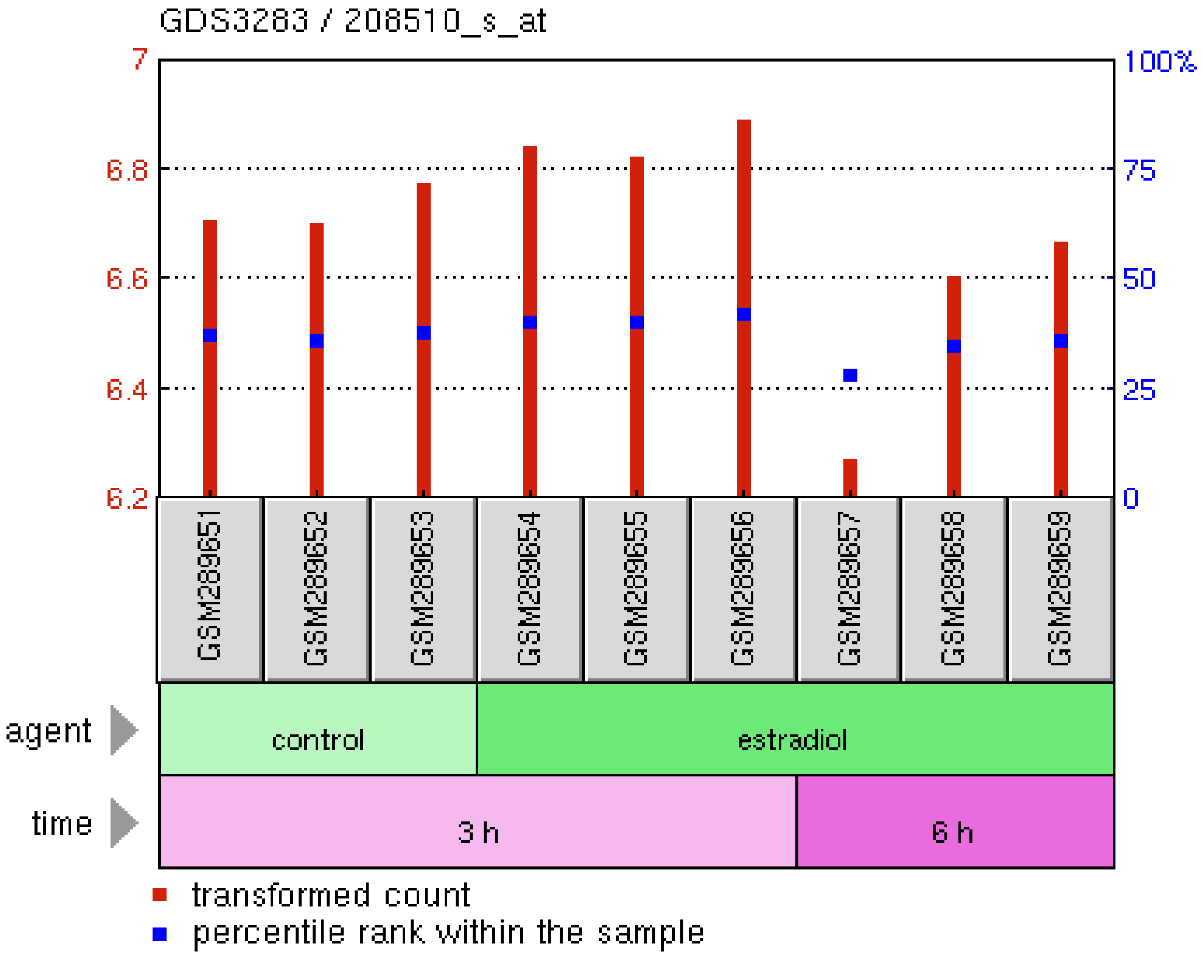

Sample	Title	Value
GSM289651	Bulun 1-3h-	6.70664
GSM289652	Bulun 2-3h-	6.70357
GSM289653	Bulun 3-3h-	6.77458
GSM289654	New 3 hr 1	6.84482
GSM289655	New 3 hr 2	6.82384
GSM289656	New 3 hr 3	6.89217
GSM289657	Bulun 1-6h+	6.27418
GSM289658	Bulun 2-6h+	6.60527
GSM289659	Bulun 3-6h+	6.66944

PPARA - Endothelial cell response to estradiol in vitro

**Profile**	GDS3600 / 1558631_at
**Title**	Endothelial cell response to estradiol in vitro
**Organism**	Homo sapiens

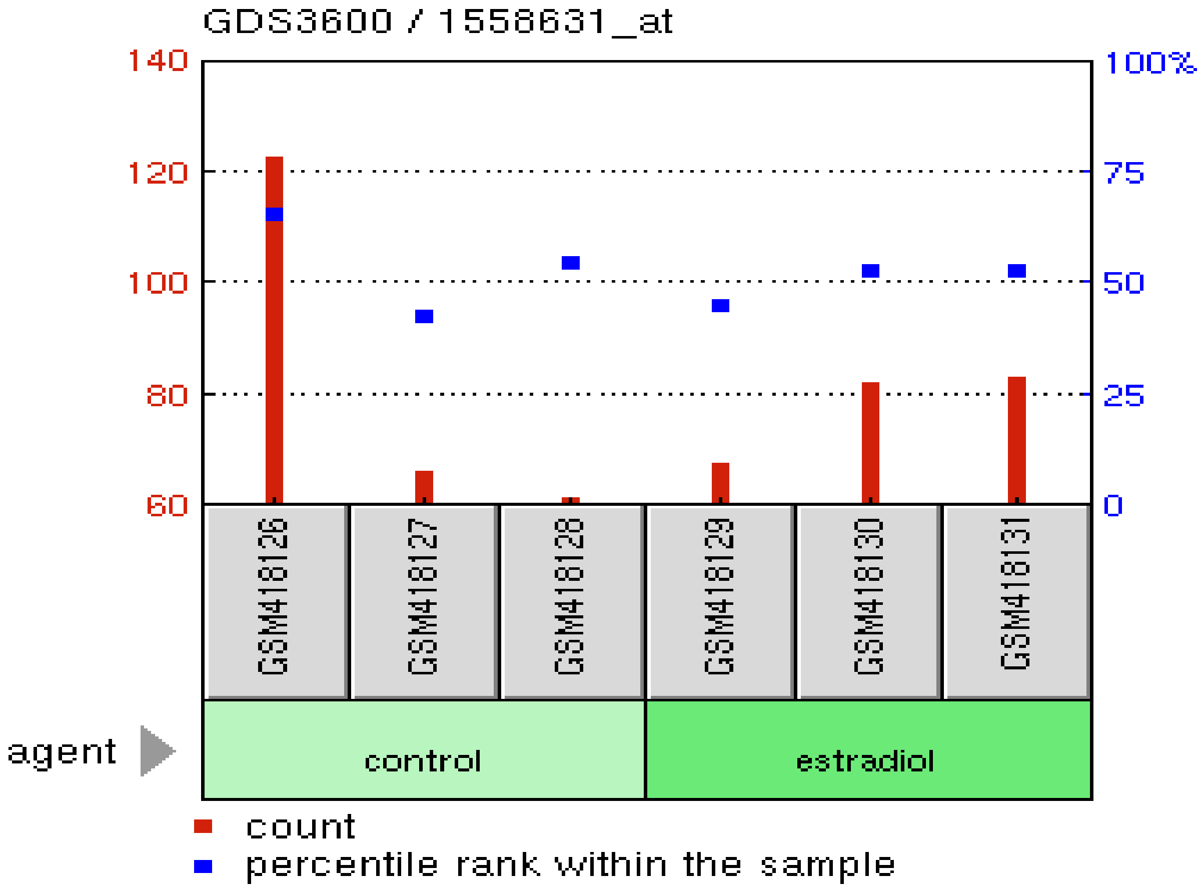

Sample	Title	Value
GSM418126	Control, rep 1	123.01
GSM418127	Control, rep 2	66.51
GSM418128	Control, rep 3	61.81
GSM418129	Estradiol, rep 1	67.98
GSM418130	Estradiol, rep 2	82.33
GSM418131	Estradiol, rep 3	83.24

PPARA - Estradiol effect on breast cancer cell line: time course

**Profile**	GDS3283 / 1558631_at
**Title**	Estradiol effect on breast cancer cell line: time course
**Organism**	Homo sapiens

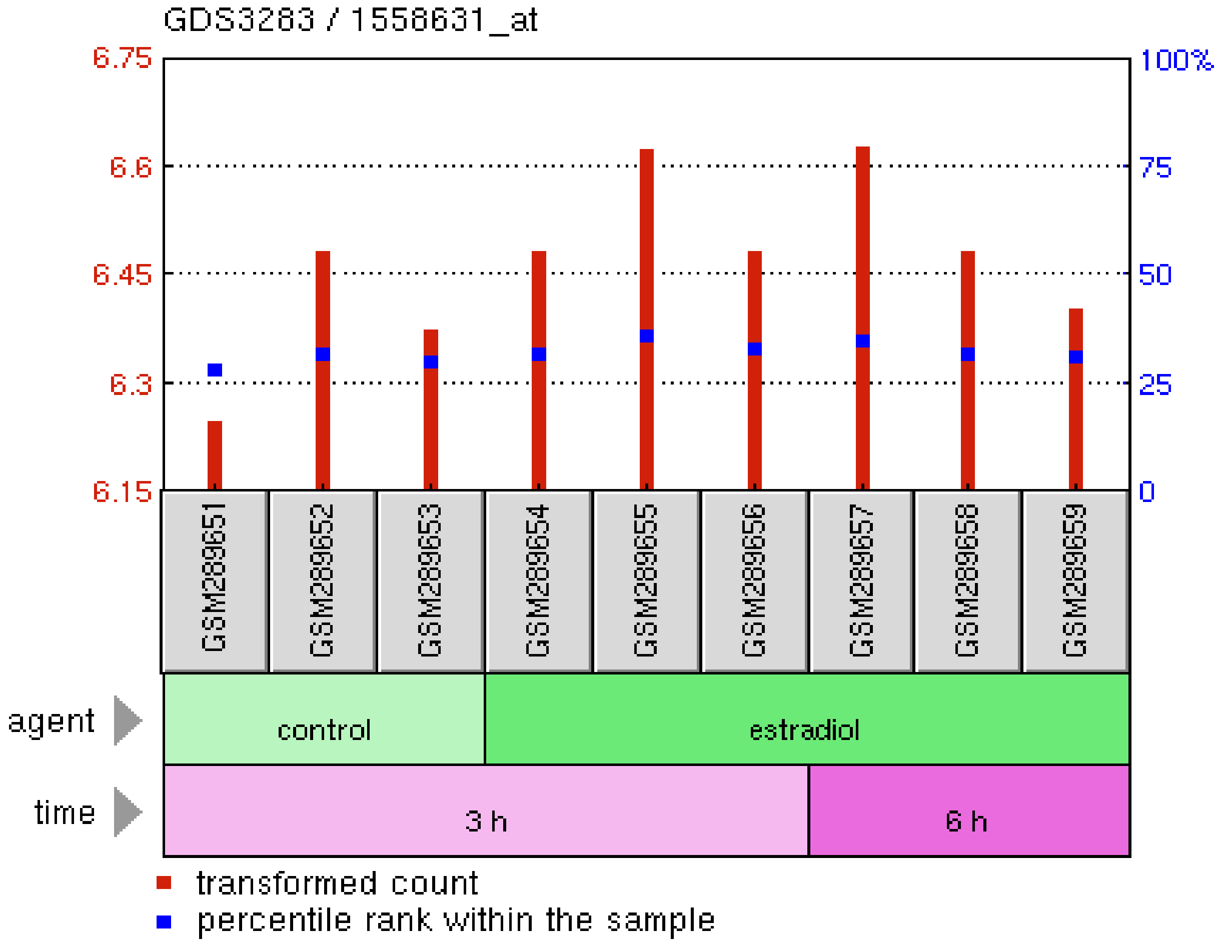

Sample	Title	Value
GSM289651	Bulun 1-3h-	6.25086
GSM289652	Bulun 2-3h-	6.486
GSM289653	Bulun 3-3h-	6.37529
GSM289654	New 3 hr 1	6.486
GSM289655	New 3 hr 2	6.62351
GSM289656	New 3 hr 3	6.486
GSM289657	Bulun 1-6h+	6.62949
GSM289658	Bulun 2-6h+	6.486
GSM289659	Bulun 3-6h+	6.40405

PPARA - Endothelial cell response to estradiol in vitro

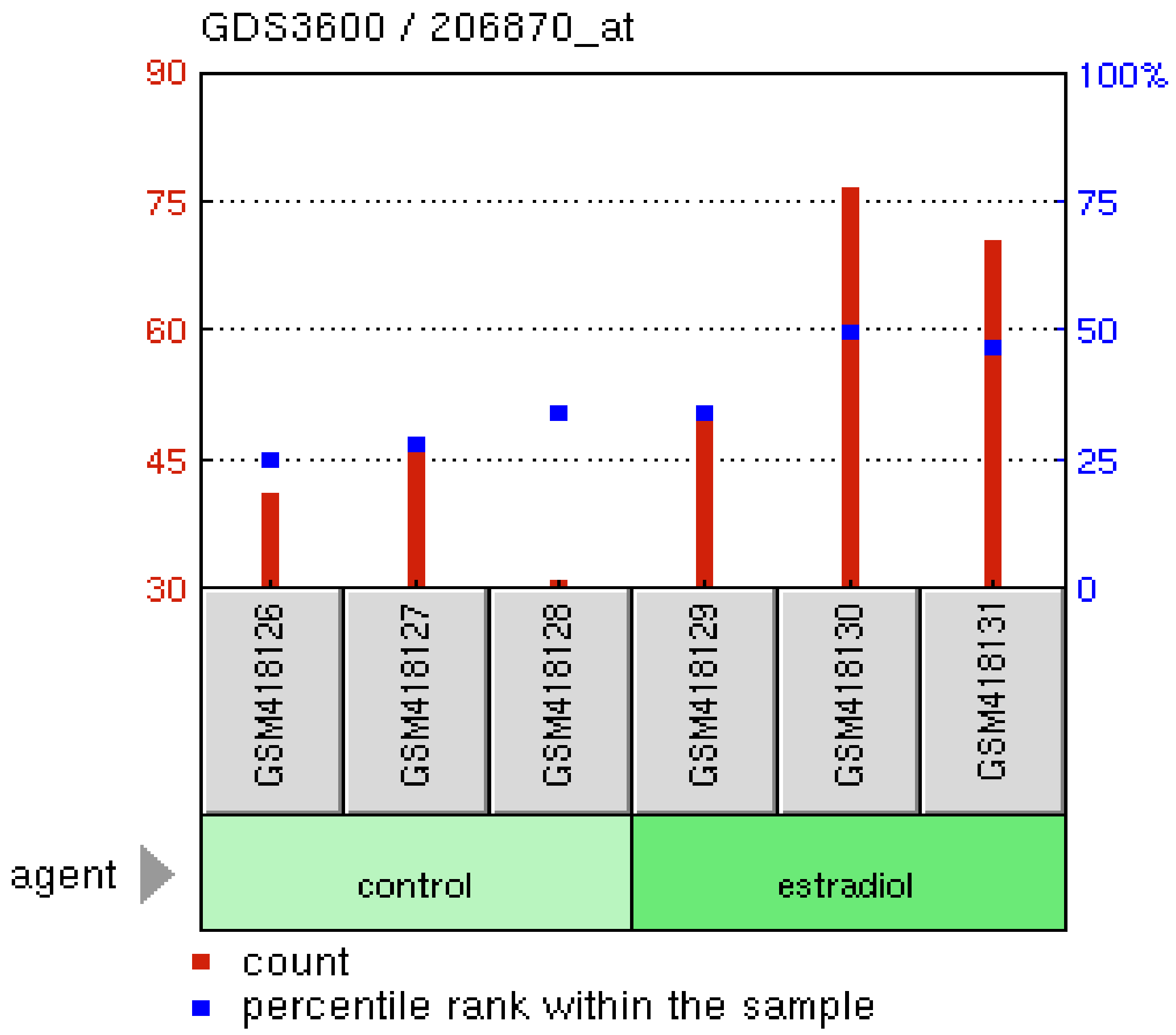

Sample	Title	Value
GSM418126	Control, rep 1	41.25
GSM418127	Control, rep 2	46.49
GSM418128	Control, rep 3	31.15
GSM418129	Estradiol, rep 1	51.59
GSM418130	Estradiol, rep 2	76.8
GSM418131	Estradiol, rep 3	70.64

SOX17 - Endothelial cell response to estradiol in vitro

**Profile**	GDS3600 / 230943_at
**Title**	Endothelial cell response to estradiol in vitro
**Organism**	Homo sapiens

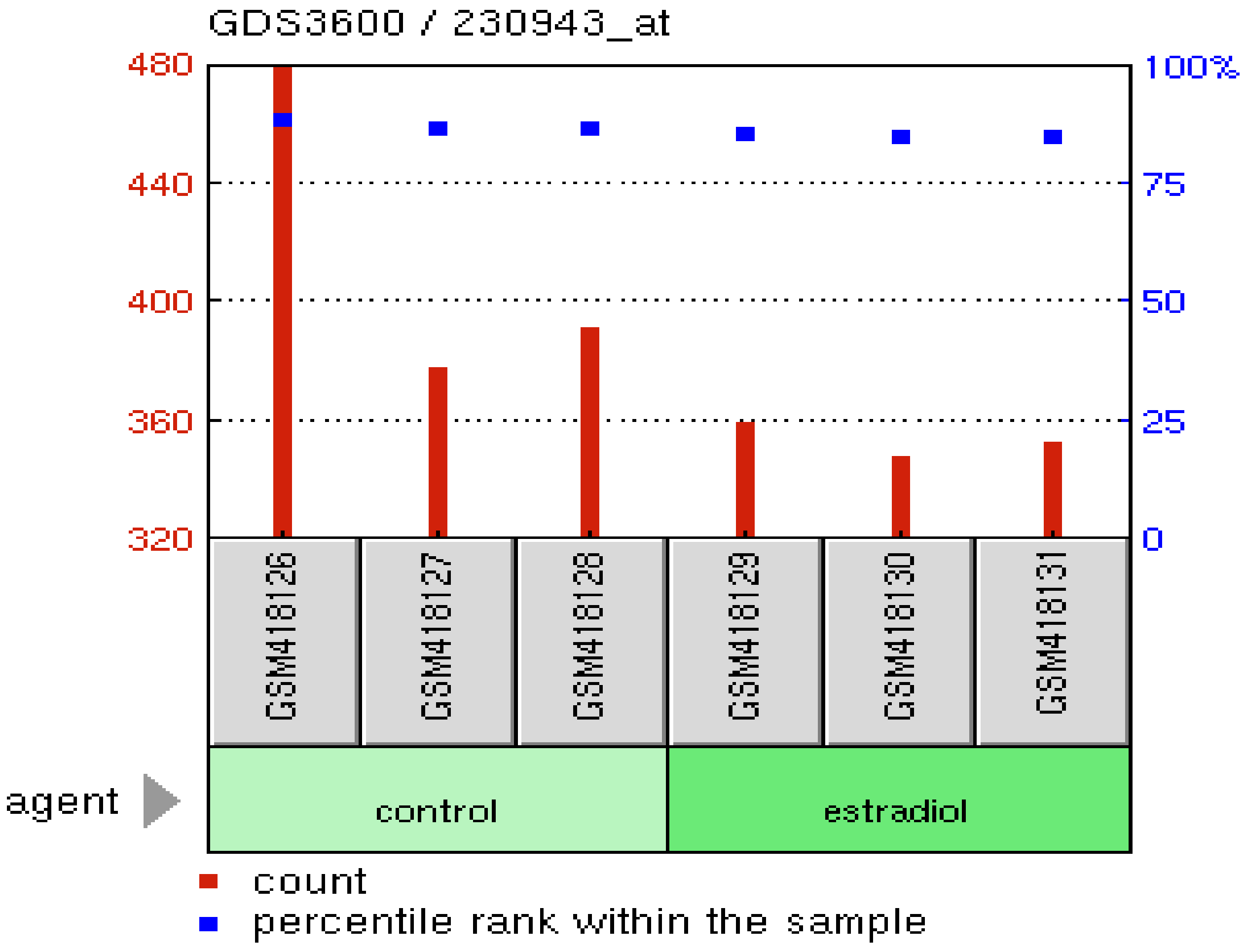

Sample	Title	Value
GSM418126	Control, rep 1	479.89
GSM418127	Control, rep 2	378.72
GSM418128	Control, rep 3	391.59
GSM418129	Estradiol, rep 1	359.99
GSM418130	Estradiol, rep 2	348
GSM418131	Estradiol, rep 3	353.19

Runx1 - Estradiol effect on the ischemic brain

**Profile**	GDS2311 / L35271_at
**Title**	Estradiol effect on the ischemic brain
**Organism**	Rattus norvegicus

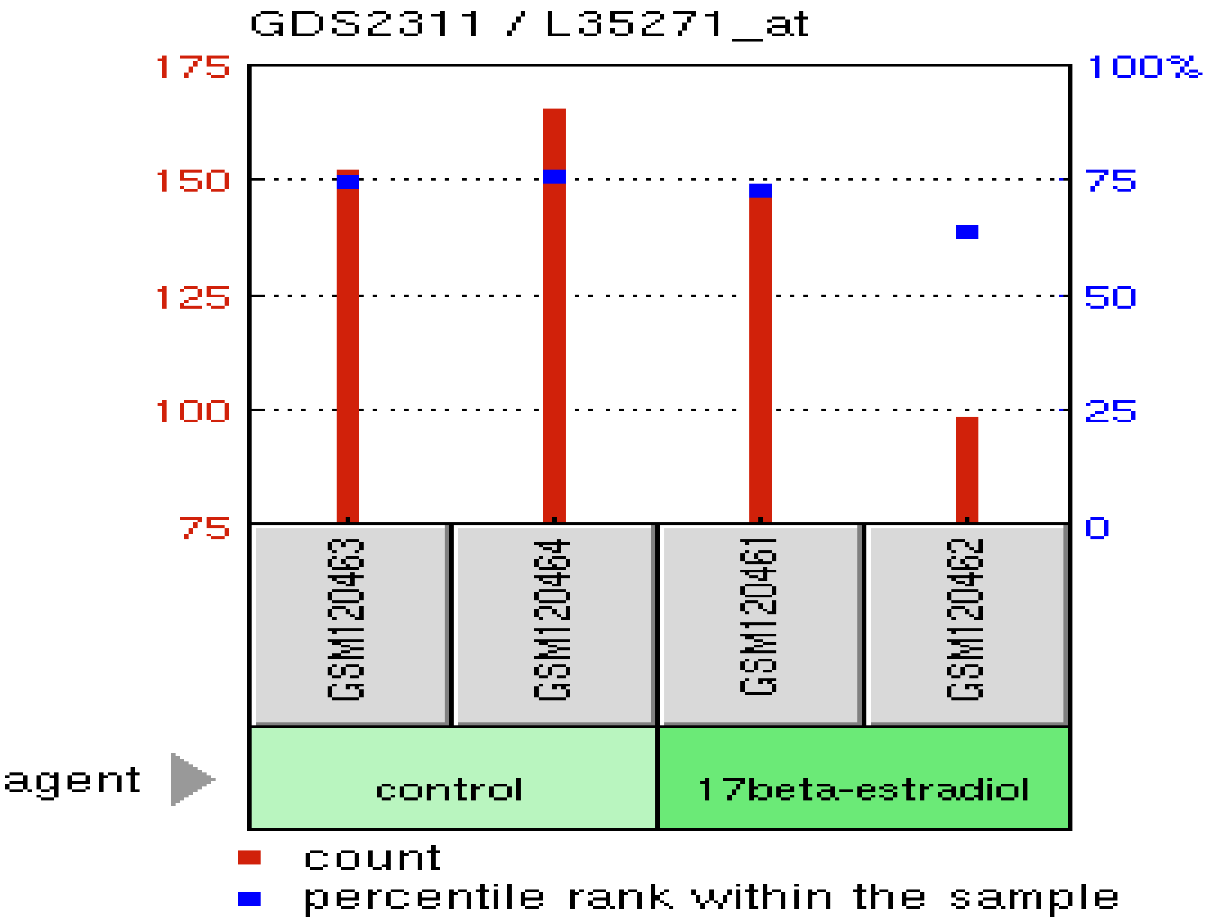

Sample	Title	Value
GSM120463	Placebo treated ipsilateral to stroke P6IC-1a	152.4
GSM120464	Placebo treated ipsilateral to stroke replicate P6IC-2a	165.7
GSM120461	Estrogen treated ipsilateral to stroke E6IC-1a	146.7
GSM120462	Estrogen treated ipsilateral to stroke replicate E6IC-2a	98.5

PPARG - All-trans retinoic acid (ATRA) vitamin A and 1,25-dihydroxyvitamin D3 (1,25D3) vitamin D stimulated peripheral blood monocytes

**Profile**	GDS4860 / 208510_s_at
**Title**	All-trans retinoic acid (ATRA) vitamin A and 1,25-dihydroxyvitamin D3 (1,25D3) vitamin D stimulated peripheral blood monocytes
**Organism**	Homo sapiens

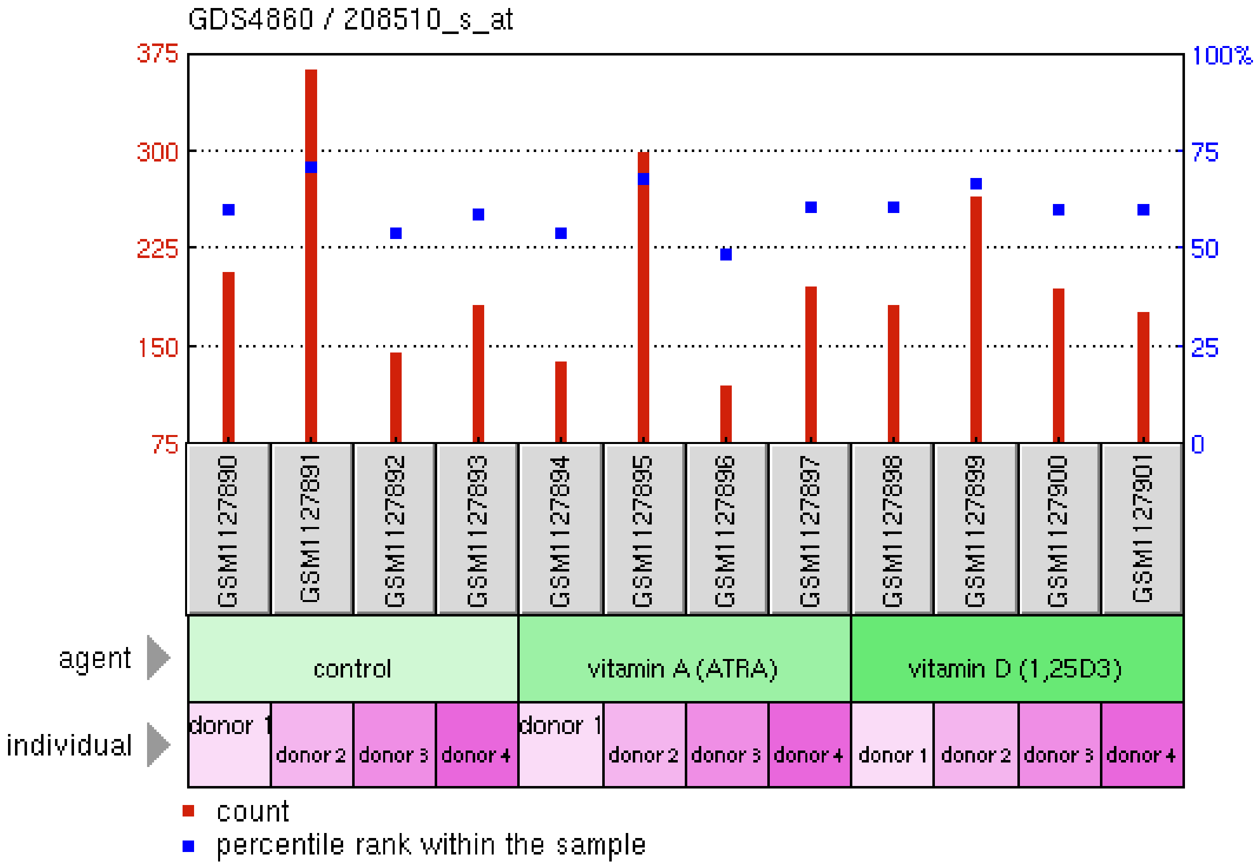

Sample	Title	Value
GSM1127890	monocyte from donor 1 stimulated with CTRL	207.327
GSM1127891	monocyte from donor 2 stimulated with CTRL	363.093
GSM1127892	monocyte from donor 3 stimulated with CTRL	145.655
GSM1127893	monocyte from donor 4 stimulated with CTRL	181.838
GSM1127894	monocyte from donor 1 stimulated with ATRA	138.577
GSM1127895	monocyte from donor 2 stimulated with ATRA	300.076
GSM1127896	monocyte from donor 3 stimulated with ATRA	120.906
GSM1127897	monocyte from donor 4 stimulated with ATRA	196.165
GSM1127898	monocyte from donor 1 stimulated with 1,25D	182.698
GSM1127899	monocyte from donor 2 stimulated with 1,25D	265.993
GSM1127900	monocyte from donor 3 stimulated with 1,25D	195.055
GSM1127901	monocyte from donor 4 stimulated with 1,25D	177.124

PPARG - All-trans retinoic acid (ATRA) vitamin A and 1,25-dihydroxyvitamin D3 (1,25D3) vitamin D stimulated peripheral blood monocytes

**Profile**	GDS4860 / 208510_s_at
**Title**	All-trans retinoic acid (ATRA) vitamin A and 1,25-dihydroxyvitamin D3 (1,25D3) vitamin D stimulated peripheral blood monocytes
**Organism**	Homo sapiens

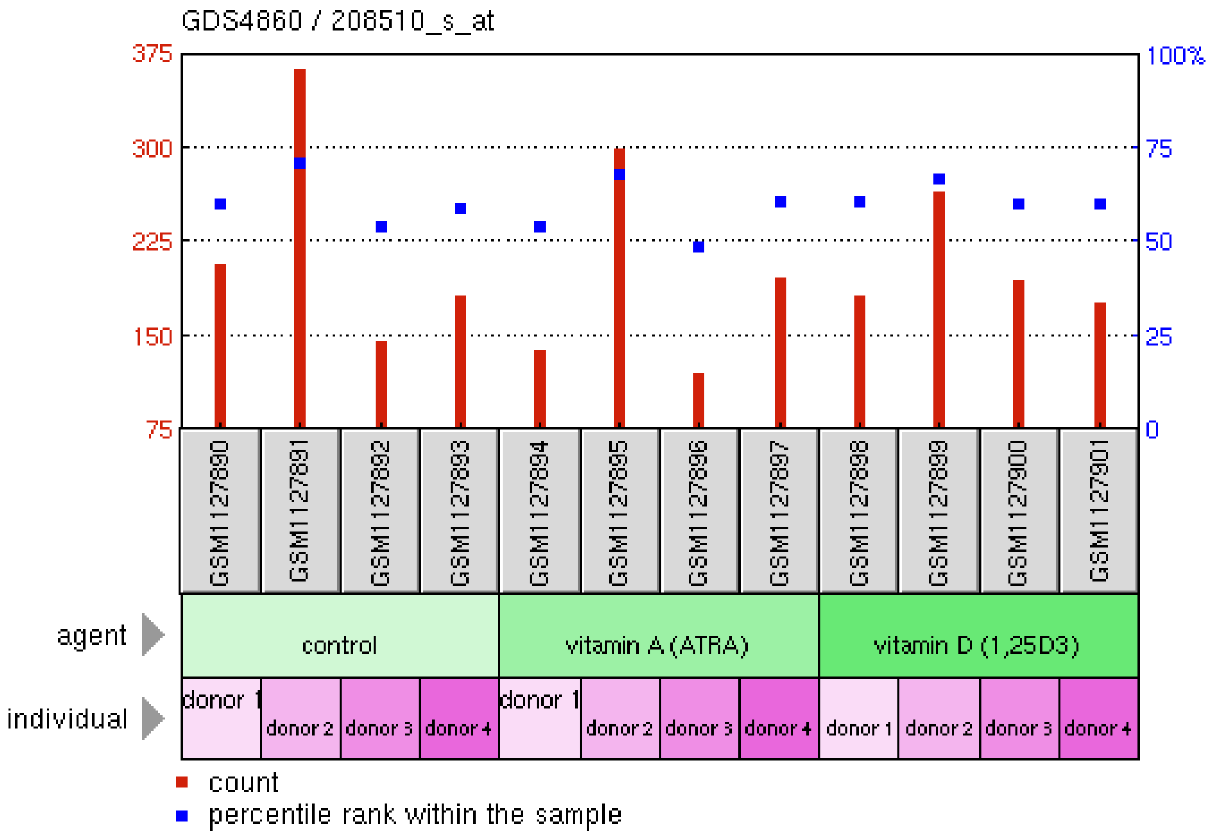

Sample	Title	Value
GSM1127890	monocyte from donor 1 stimulated with CTRL	207.327
GSM1127891	monocyte from donor 2 stimulated with CTRL	363.093
GSM1127892	monocyte from donor 3 stimulated with CTRL	145.655
GSM1127893	monocyte from donor 4 stimulated with CTRL	181.838
GSM1127894	monocyte from donor 1 stimulated with ATRA	138.577
GSM1127895	monocyte from donor 2 stimulated with ATRA	300.076
GSM1127896	monocyte from donor 3 stimulated with ATRA	120.906
GSM1127897	monocyte from donor 4 stimulated with ATRA	196.165
GSM1127898	monocyte from donor 1 stimulated with 1,25D	182.698
GSM1127899	monocyte from donor 2 stimulated with 1,25D	265.993
GSM1127900	monocyte from donor 3 stimulated with 1,25D	195.055
GSM1127901	monocyte from donor 4 stimulated with 1,25D	177.124

PPARG - Trans-retinoic acid and hydrocortisone induction of Na+/I- symporter in MCF-7 breast cancer cells

Profile GDS4082 / 208510_s_at

Title Trans-retinoic acid and hydrocortisone induction of Na+/I- symporter in MCF-7 breast cancer cells

Organism Homo sapiens

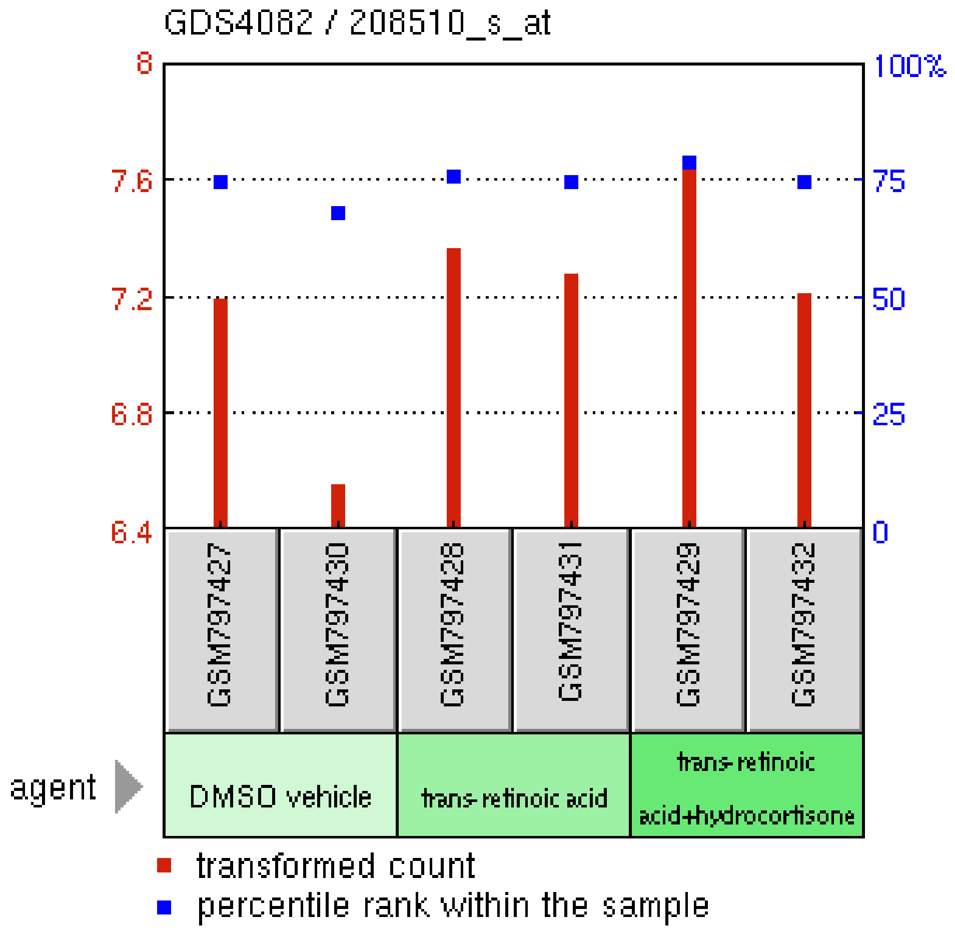

Sample	Title	Value
GSM797427	MCF-7 cell_DMSO_12hr_biological rep1	7.19145
GSM797430	MCF-7 cell_DMSO_12hr_biological rep2	6.56237
GSM797428	MCF-7 cell_tRA_12hr_biological rep1	7.37256
GSM797431	MCF-7 cell_tRA_12hr_biological rep2	7.27727
GSM797429	MCF-7 cell_tRA-H_12hr_biological rep1	7.64706
GSM797432	MCF-7 cell_tRA-H_12hr_biological rep2	7.21168

PPARA - All-trans retinoic acid (ATRA) vitamin A and 1,25-dihydroxyvitamin D3 (1,25D3) vitamin D stimulated peripheral blood monocytes

Profile GDS4860 / 223438_s_at

Title All-trans retinoic acid (ATRA) vitamin A and 1,25-dihydroxyvitamin D3 (1,25D3) vitamin D stimulated peripheral blood monocytes

Organism

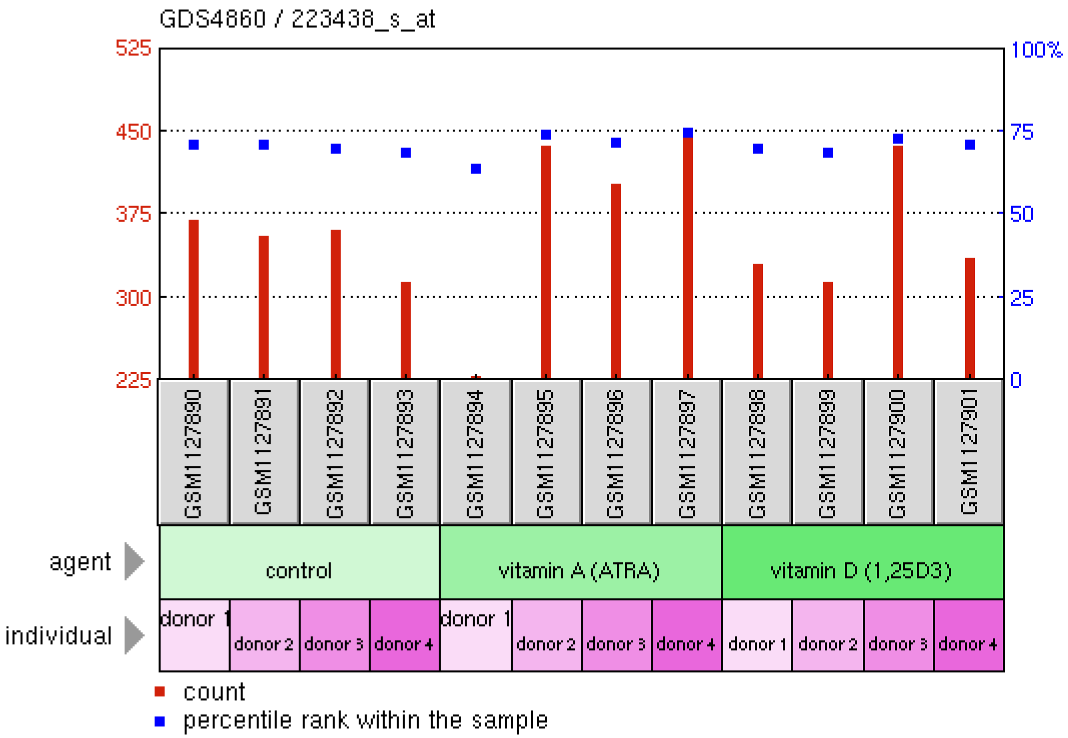

Sample	Title	Value	Rank
GSM1127890	monocyte from donor 1 stimulated with CTRL	370.684	71
GSM1127891	monocyte from donor 2 stimulated with CTRL	356.049	71
GSM1127892	monocyte from donor 3 stimulated with CTRL	361.137	70
GSM1127893	monocyte from donor 4 stimulated with CTRL	313.866	69
GSM1127894	monocyte from donor 1 stimulated with ATRA	229.664	64
GSM1127895	monocyte from donor 2 stimulated with ATRA	437.36	74
GSM1127896	monocyte from donor 3 stimulated with ATRA	402.443	72
GSM1127897	monocyte from donor 4 stimulated with ATRA	450.919	75
GSM1127898	monocyte from donor 1 stimulated with 1,25D	330.744	70
GSM1127899	monocyte from donor 2 stimulated with 1,25D	314.203	69
GSM1127900	monocyte from donor 3 stimulated with 1,25D	436.838	73
GSM1127901	monocyte from donor 4 stimulated with 1,25D	335.629	71

PPARA - Trans-retinoic acid and hydrocortisone induction of Na+/I- symporter in MCF-7 breast cancer cells

Profile GDS4082 / 223438_s_at

Title Trans-retinoic acid and hydrocortisone induction of Na+/I- symporter in MCF-7 breast cancer cells

Organism Homo sapiens

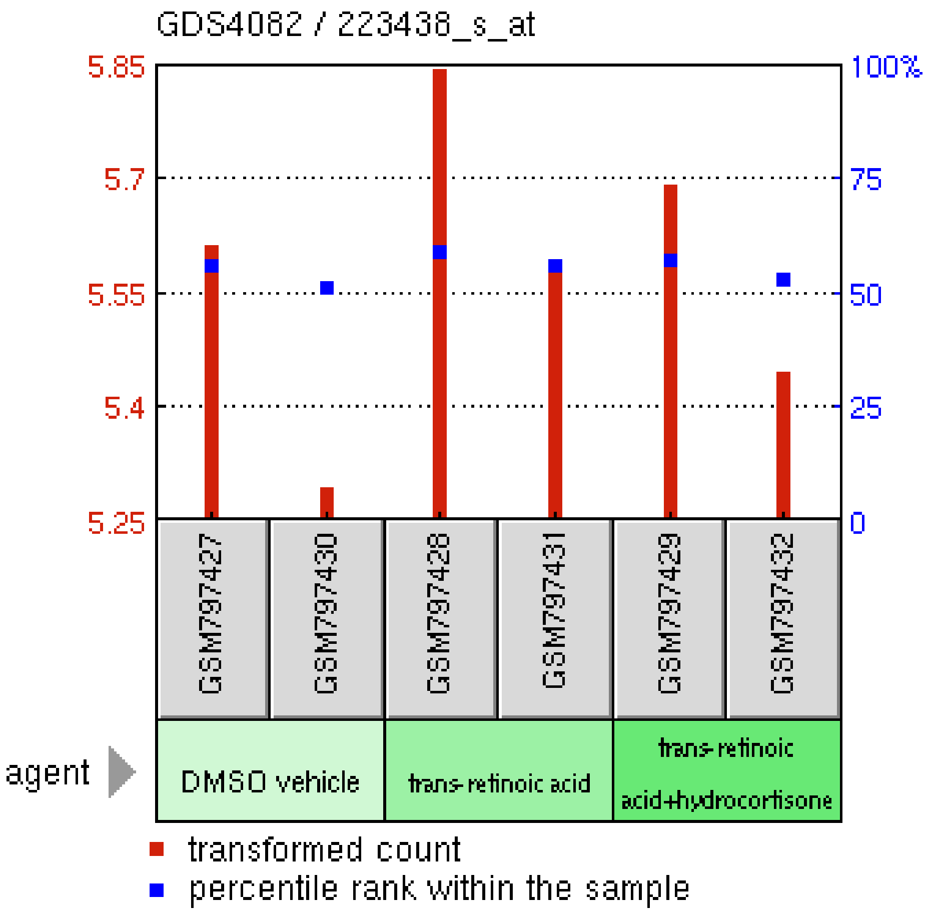

Sample	Title	Value
GSM797427	MCF-7 cell_DMSO_12hr_biological rep1	5.61195
GSM797430	MCF-7 cell_DMSO_12hr_biological rep2	5.29527
GSM797428	MCF-7 cell_tRA_12hr_biological rep1	5.8455
GSM797431	MCF-7 cell_tRA_12hr_biological rep2	5.58719
GSM797429	MCF-7 cell_tRA-H_12hr_biological rep1	5.69397
GSM797432	MCF-7 cell_tRA-H_12hr_biological rep2	5.44866

SOX17 - All-trans retinoic acid (ATRA) vitamin A and 1,25-dihydroxyvitamin D3 (1,25D3) vitamin D stimulated peripheral blood monocytes

Profile GDS4860 / 230943_at

Title All-trans retinoic acid (ATRA) vitamin A and 1,25-dihydroxyvitamin D3 (1,25D3) vitamin D stimulated peripheral blood monocytes

Organism Homo sapiens

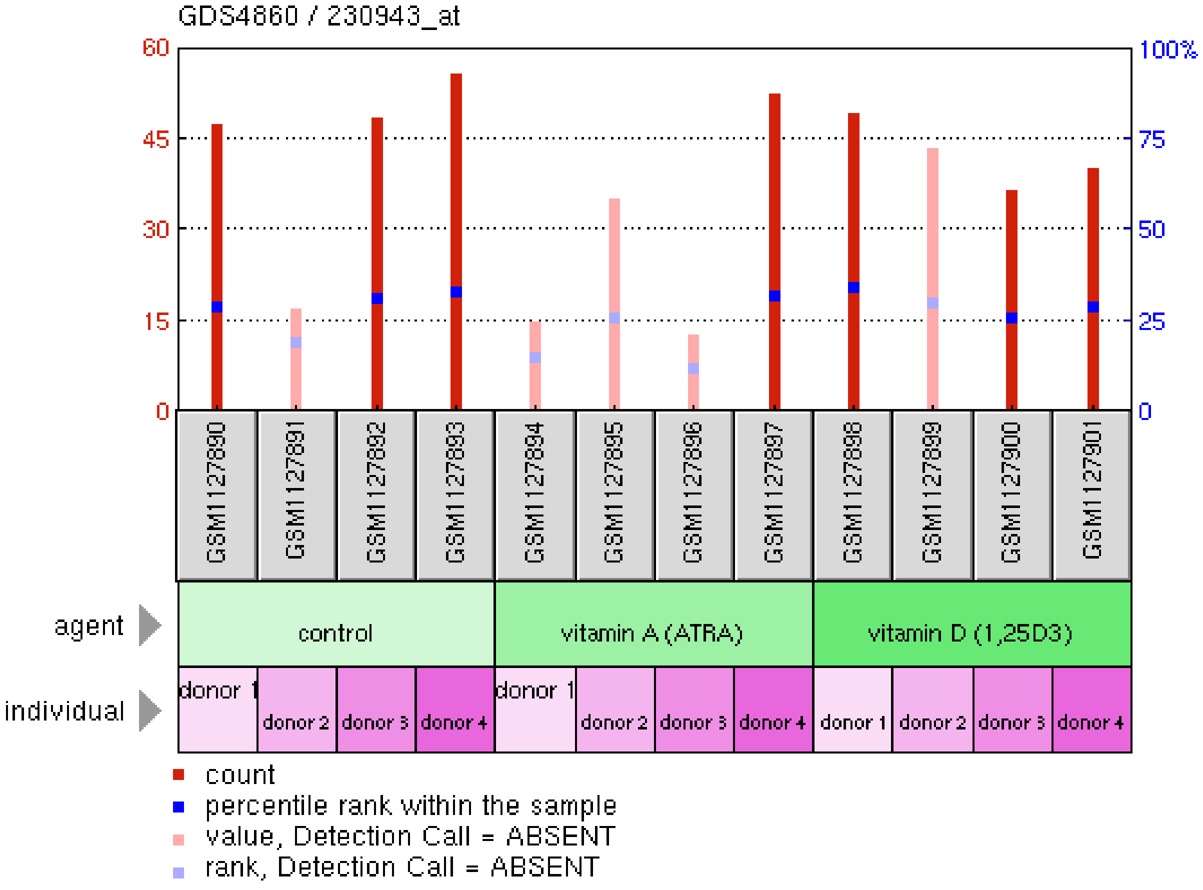

Sample	Title	Value
GSM1127890	monocyte from donor 1 stimulated with CTRL	47.5317
GSM1127891	monocyte from donor 2 stimulated with CTRL	17.2277
GSM1127892	monocyte from donor 3 stimulated with CTRL	48.5429
GSM1127893	monocyte from donor 4 stimulated with CTRL	55.733
GSM1127894	monocyte from donor 1 stimulated with ATRA	14.9672
GSM1127895	monocyte from donor 2 stimulated with ATRA	35.1071
GSM1127896	monocyte from donor 3 stimulated with ATRA	12.9649
GSM1127897	monocyte from donor 4 stimulated with ATRA	52.5423
GSM1127898	monocyte from donor 1 stimulated with 1,25D	49.2975
GSM1127899	monocyte from donor 2 stimulated with 1,25D	43.6532
GSM1127900	monocyte from donor 3 stimulated with 1,25D	36.5967
GSM1127901	monocyte from donor 4 stimulated with 1,25D	40.3322

Supplementary Figure 14. Effect of Estradiol and Retinoic acid as putative modulators of transcription factors targeting key genes, TMPRSS2 and CTSB/L. Both Estradiol and retinoic acid increases the expression of POU5F1 and AIRE in various cells.

Pou5f1 - Uterus response to 17beta-estradiol: time course

**Profile**	GDS1058 / 103075_at
**Title**	Uterus response to 17beta-estradiol: time course
**Organism**	Mus musculus

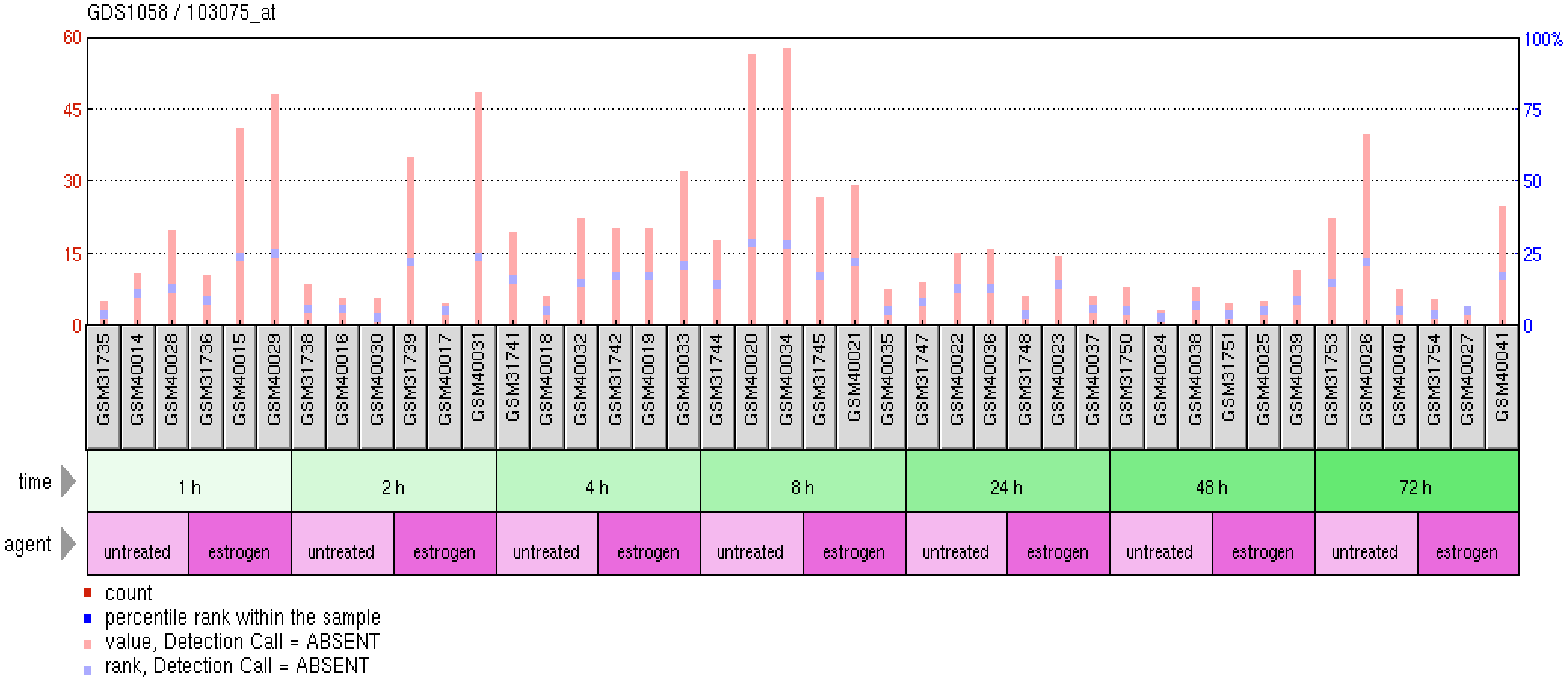

POU5F1 - Endometrium response to tamoxifen and low dose **estradiol** combination therapy

Profile GDS5278 / MmuSTS.2285.1.S1_at

Title Endometrium response to tamoxifen and low dose estradiol combination therapy

Organism Macaca mulatta

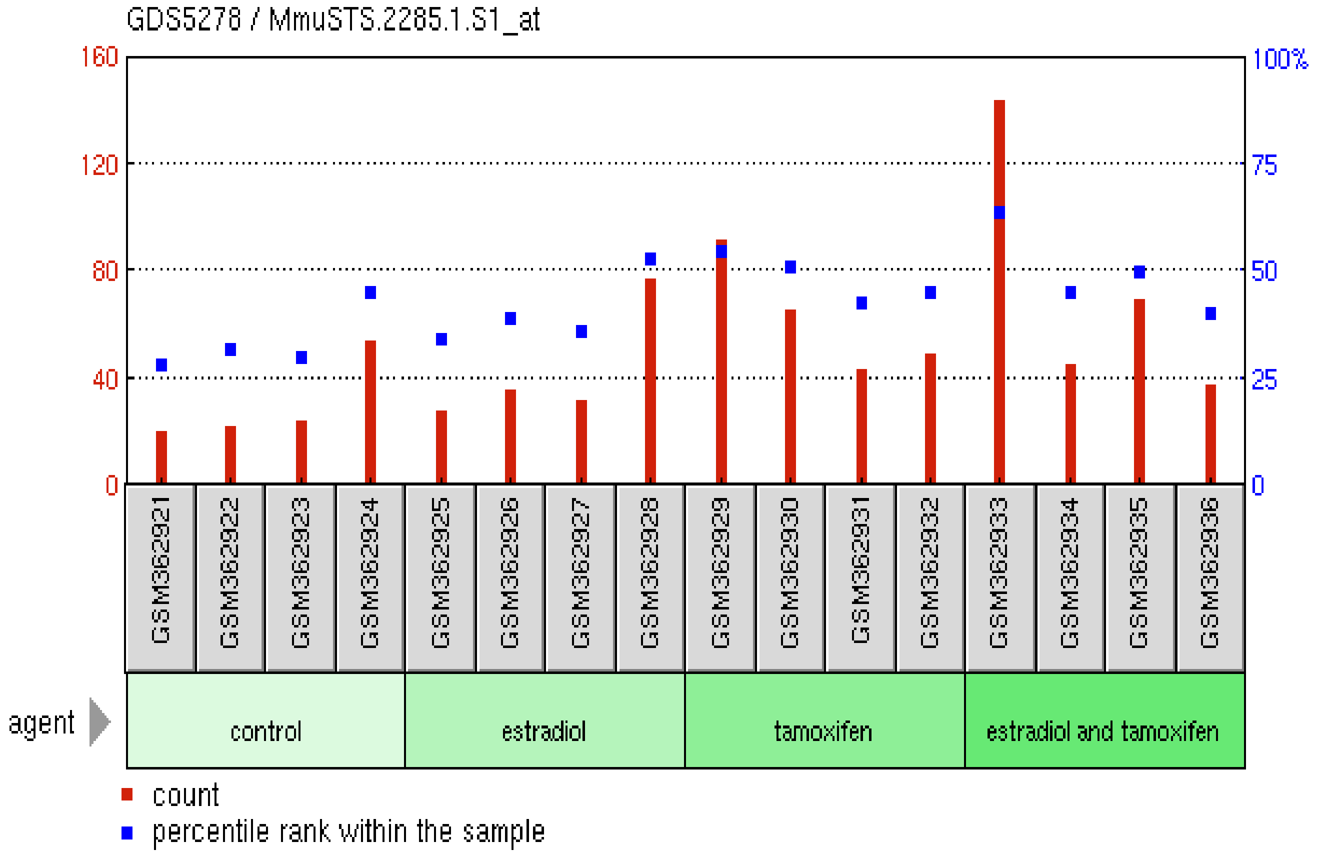

Sample	Title	Value
GSM362921	Control 1	20.8885
GSM362922	Control 2	22.6518
GSM362923	Control 3	24.1384
GSM362924	Control 4	54.7128
GSM362925	Estradiol 1	28.1372
GSM362926	Estradiol 2	36.2051
GSM362927	Estradiol 3	31.9254
GSM362928	Estradiol 4	77.119
GSM362929	Tamoxifen 1	92.0267
GSM362930	Tamoxifen 2	66.4209
GSM362931	Tamoxifen 3	44.0479
GSM362932	Tamoxifen 4	49.8232
GSM362933	Estradiol and Tamoxifen 1	144.056
GSM362934	Estradiol and Tamoxifen 2	46.257
GSM362935	Estradiol and Tamoxifen 3	69.7781
GSM362936	Estradiol and Tamoxifen 4	37.8983

AIRE - Endometrium response to tamoxifen and low dose estradiol combination therapy

**Profile**	GDS5278 / MmugDNA.17322.1.S1_at
**Title**	Endometrium response to tamoxifen and low dose estradiol combination therapy
**Organism**	Macaca mulatta

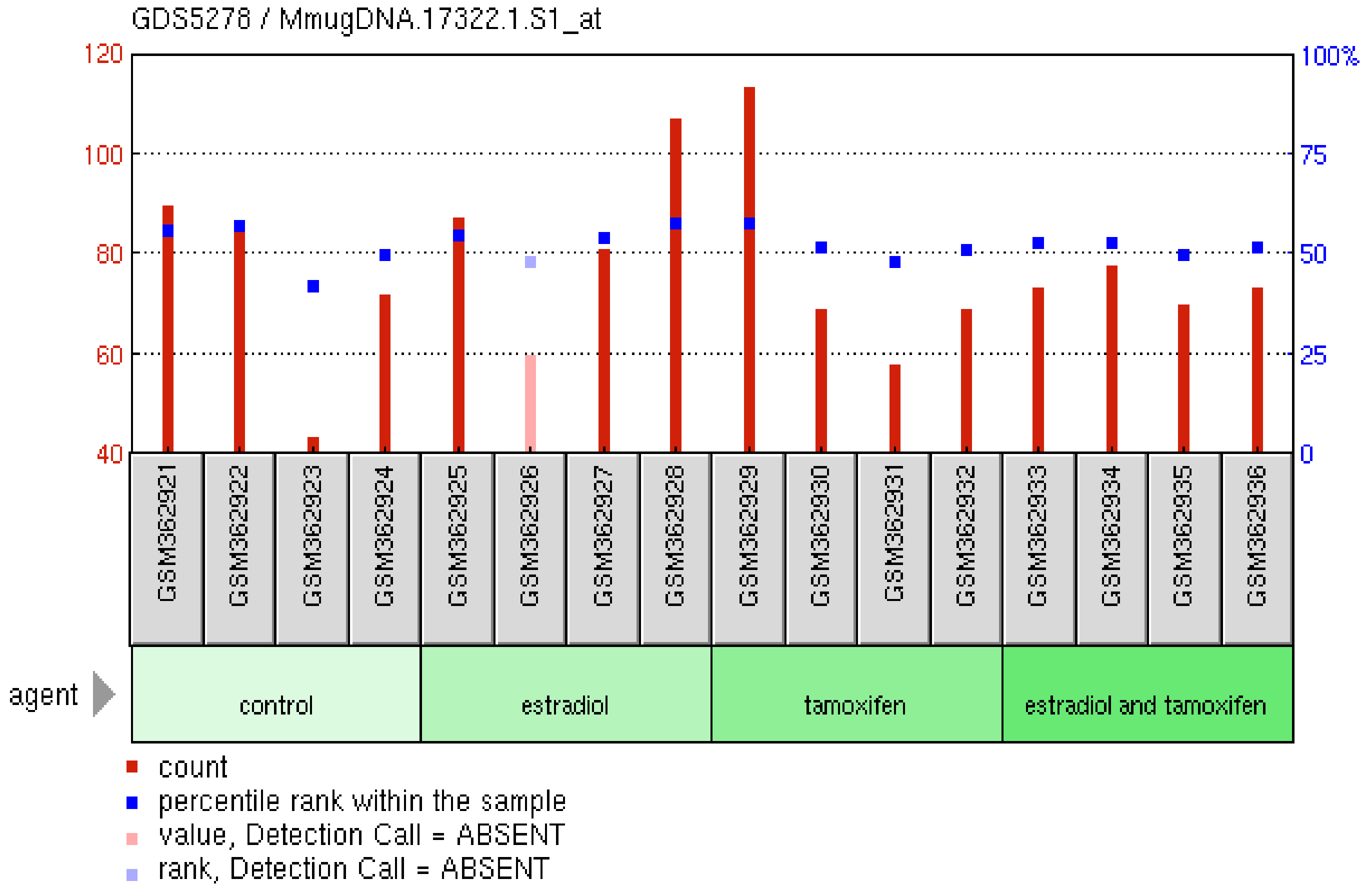

Sample	Title	Value
GSM362921	Control 1	89.668
GSM362922	Control 2	87.1643
GSM362923	Control 3	43.7563
GSM362924	Control 4	71.982
GSM362925	Estradiol 1	87.5857
GSM362926	Estradiol 2	59.7849
GSM362927	Estradiol 3	80.9651
GSM362928	Estradiol 4	107.353
GSM362929	Tamoxifen 1	113.259
GSM362930	Tamoxifen 2	69.3022
GSM362931	Tamoxifen 3	57.8455
GSM362932	Tamoxifen 4	69.0462
GSM362933	Estradiol and Tamoxifen 1	73.5671
GSM362934	Estradiol and Tamoxifen 2	77.8509
GSM362935	Estradiol and Tamoxifen 3	69.9955
GSM362936	Estradiol and Tamoxifen 4	73.2797

Aire - Uterus response to 17beta-estradiol: time course

**Profile**	GDS1058 / 97159_at
**Title**	Uterus response to 17beta-estradiol: time course
**Organism**	Mus musculus

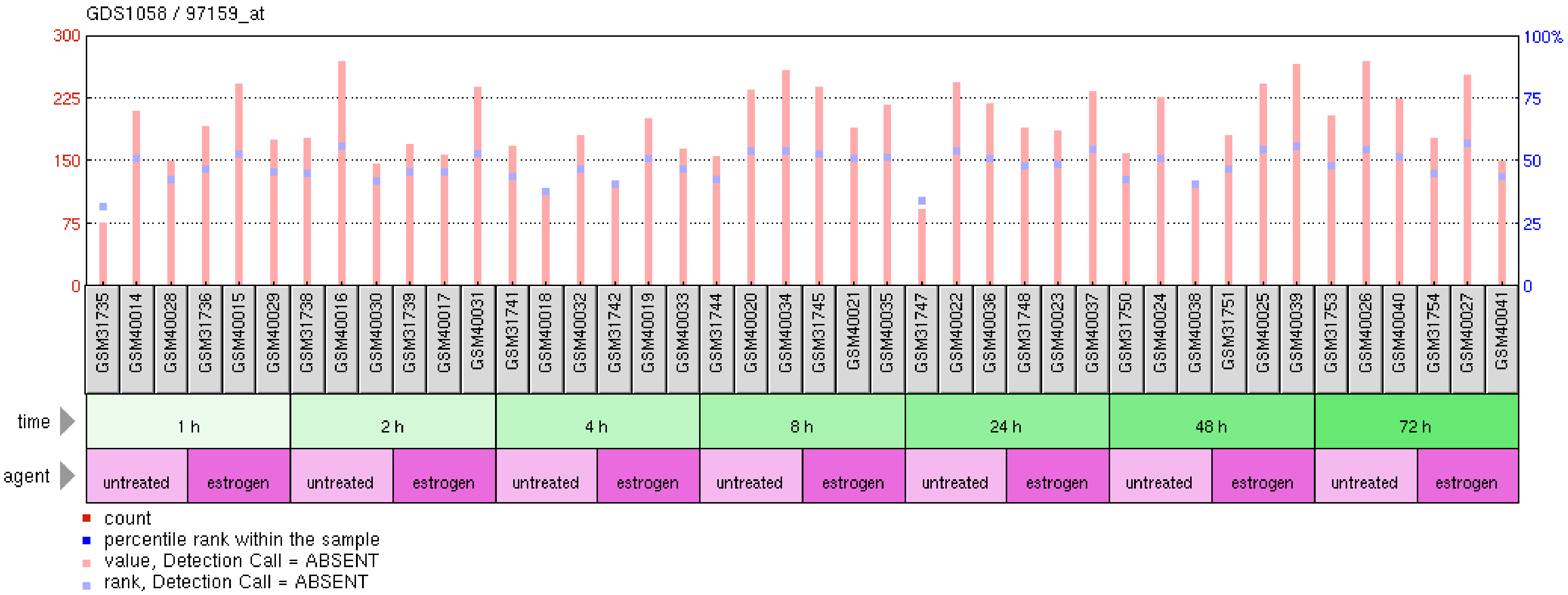

Pou5f1 - Retinoic acid effect on CD4+ T cells from spleen/lymph nodes

Profile GDS3840 / 1417945_at

Title Retinoic acid effect on CD4+ T cells from spleen/lymph nodes

Organism Mus musculus

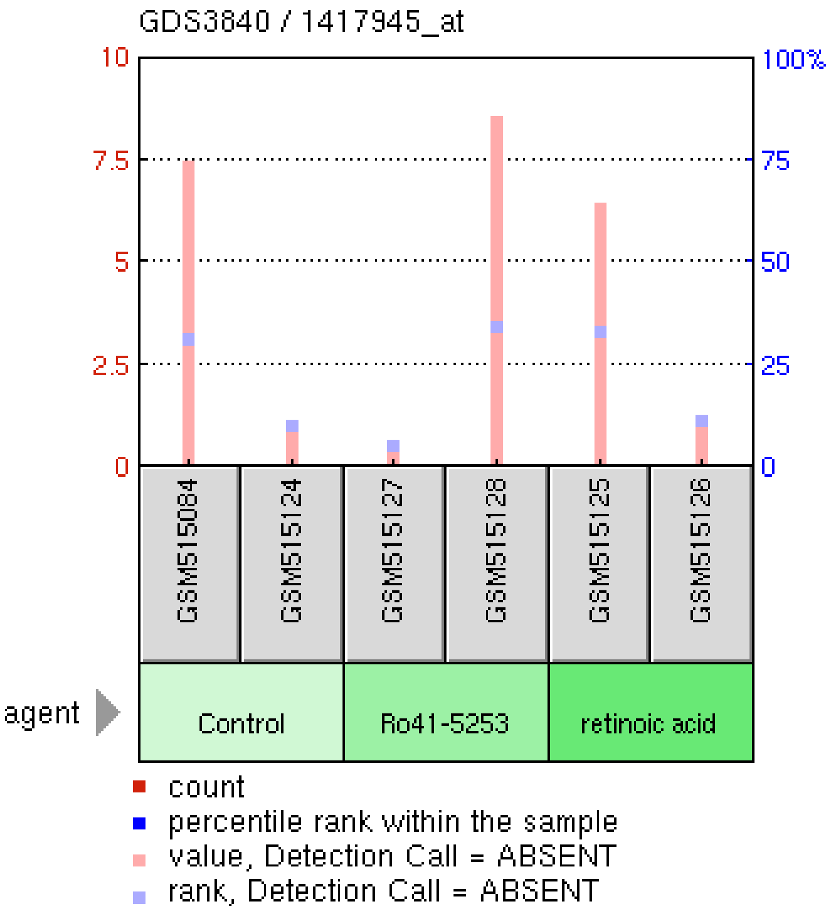

Sample	Title	Value
GSM515084	[CD4+ T-Cells]_[Control]_[1]	7.51068
GSM515124	[CD4+ T-Cells]_[Control]_[2]	1.05866
GSM515127	[CD4+ T-Cells]_[Ro]_[1]	0.665932
GSM515128	[CD4+ T-Cells]_[RO]_[2]	8.56459
GSM515125	[CD4+ T-Cells]_[RA]_[1]	6.44787
GSM515126	[CD4+ T-Cells]_[RA]_[2]	1.28111

Aire - Retinoic acid effect on CD4+ T cells from spleen/lymph nodes

Profile GDS3840 / 1419241_a_at

Title Retinoic acid effect on CD4+ T cells from spleen/lymph nodes

Organism Mus musculus

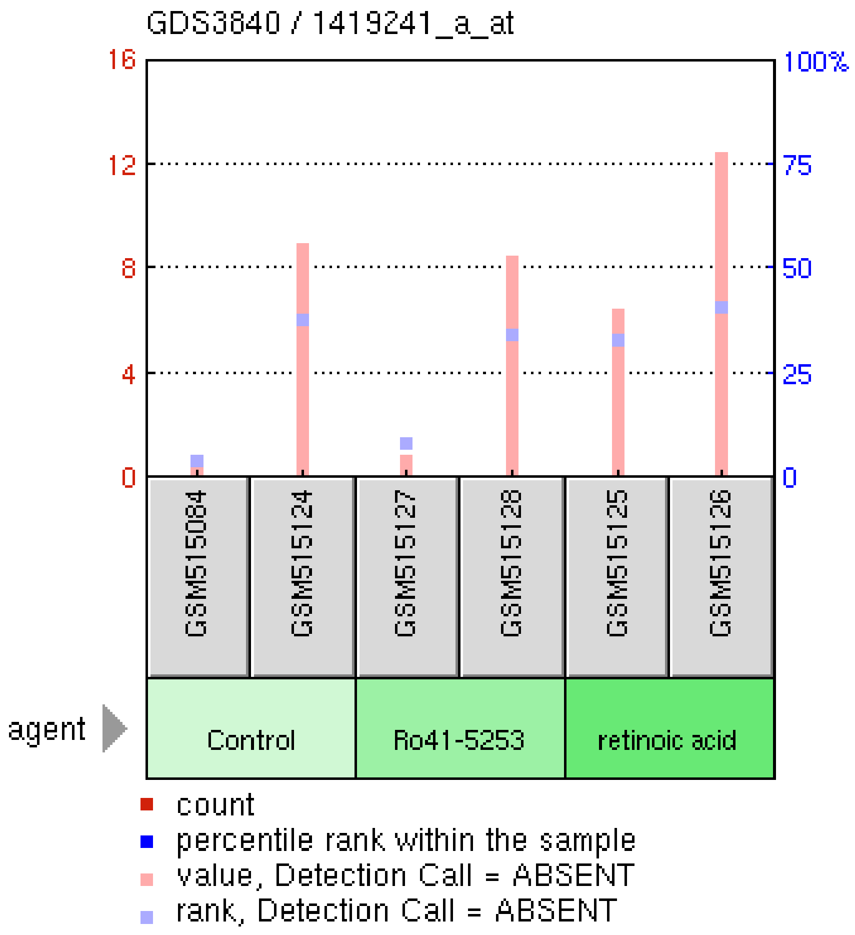

Sample	Title	Value
GSM515084	[CD4+ T-Cells]_[Control]_[1]	0.644794
GSM515124	[CD4+ T-Cells]_[Control]_[2]	8.97368
GSM515127	[CD4+ T-Cells]_[Ro]_[1]	0.895967
GSM515128	[CD4+ T-Cells]_[RO]_[2]	8.49716
GSM515125	[CD4+ T-Cells]_[RA]_[1]	6.50363
GSM515126	[CD4+ T-Cells]_[RA]_[2]	12.5205

AIRE - All-trans retinoic acid (ATRA) vitamin A and 1,25-dihydroxyvitamin D3 (1,25D3) vitamin D stimulated peripheral blood monocytes

**Profile**	GDS4860 / 208090_s_at
**Title**	All-trans retinoic acid (ATRA) vitamin A and 1,25-dihydroxyvitamin D3 (1,25D3) vitamin D stimulated peripheral blood monocytes
**Organism**	Homo sapiens

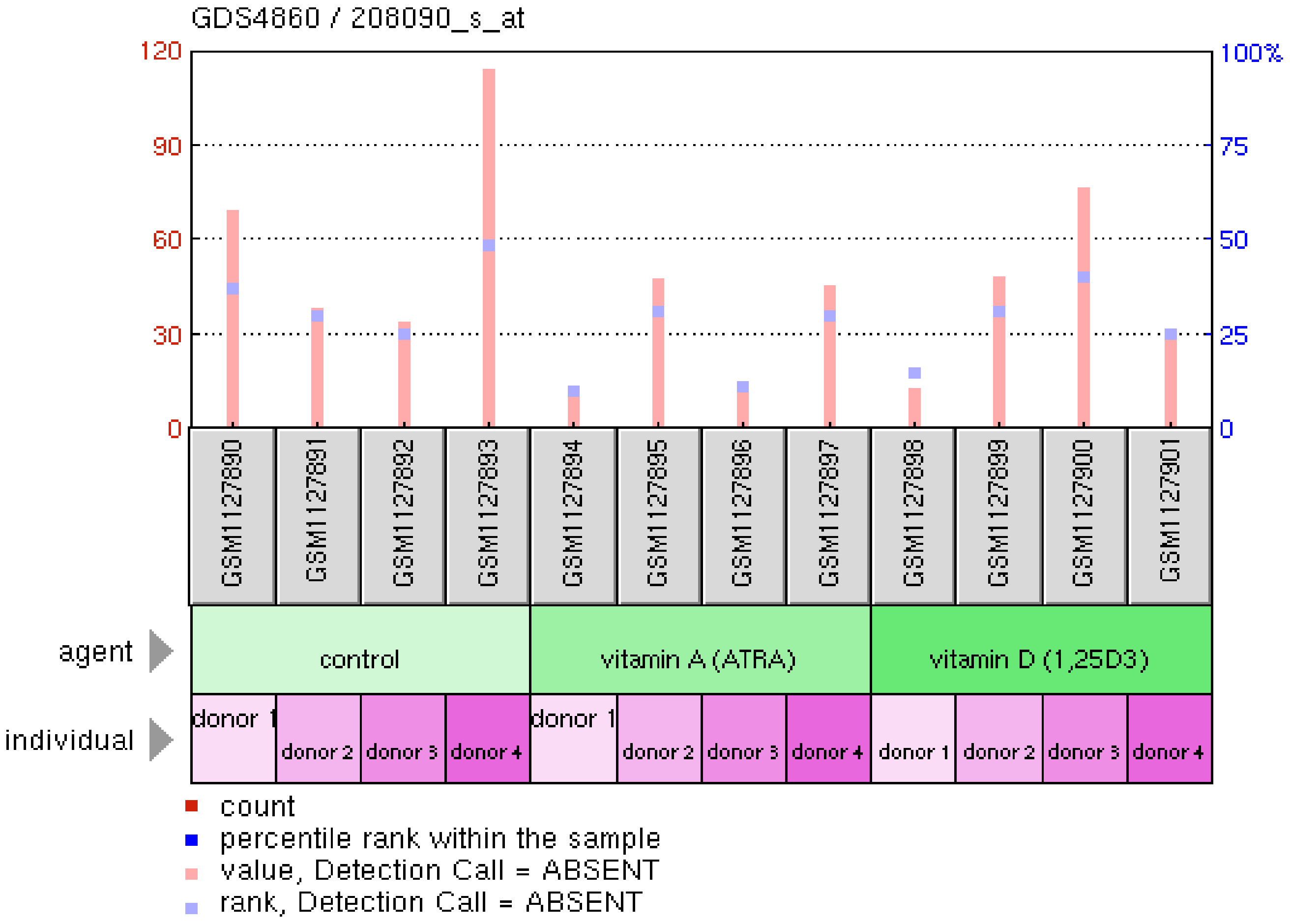

Sample	Title	Value
GSM1127890	monocyte from donor 1 stimulated with CTRL	69.8075
GSM1127891	monocyte from donor 2 stimulated with CTRL	38.9471
GSM1127892	monocyte from donor 3 stimulated with CTRL	34.6725
GSM1127893	monocyte from donor 4 stimulated with CTRL	114.731
GSM1127894	monocyte from donor 1 stimulated with ATRA	10.2908
GSM1127895	monocyte from donor 2 stimulated with ATRA	47.9749
GSM1127896	monocyte from donor 3 stimulated with ATRA	11.5769
GSM1127897	monocyte from donor 4 stimulated with ATRA	45.8082
GSM1127898	monocyte from donor 1 stimulated with 1,25D	13.4956
GSM1127899	monocyte from donor 2 stimulated with 1,25D	48.5224
GSM1127900	monocyte from donor 3 stimulated with 1,25D	76.7651
GSM1127901	monocyte from donor 4 stimulated with 1,25D	30.6412

AIRE - Trans-retinoic acid and hydrocortisone induction of Na+/I- symporter in MCF-7 breast cancer cells

Profile GDS4082 / 208090_s_at

Title Trans-retinoic acid and hydrocortisone induction of Na+/I- symporter in MCF-7 breast cancer cells

Organism Homo sapiens

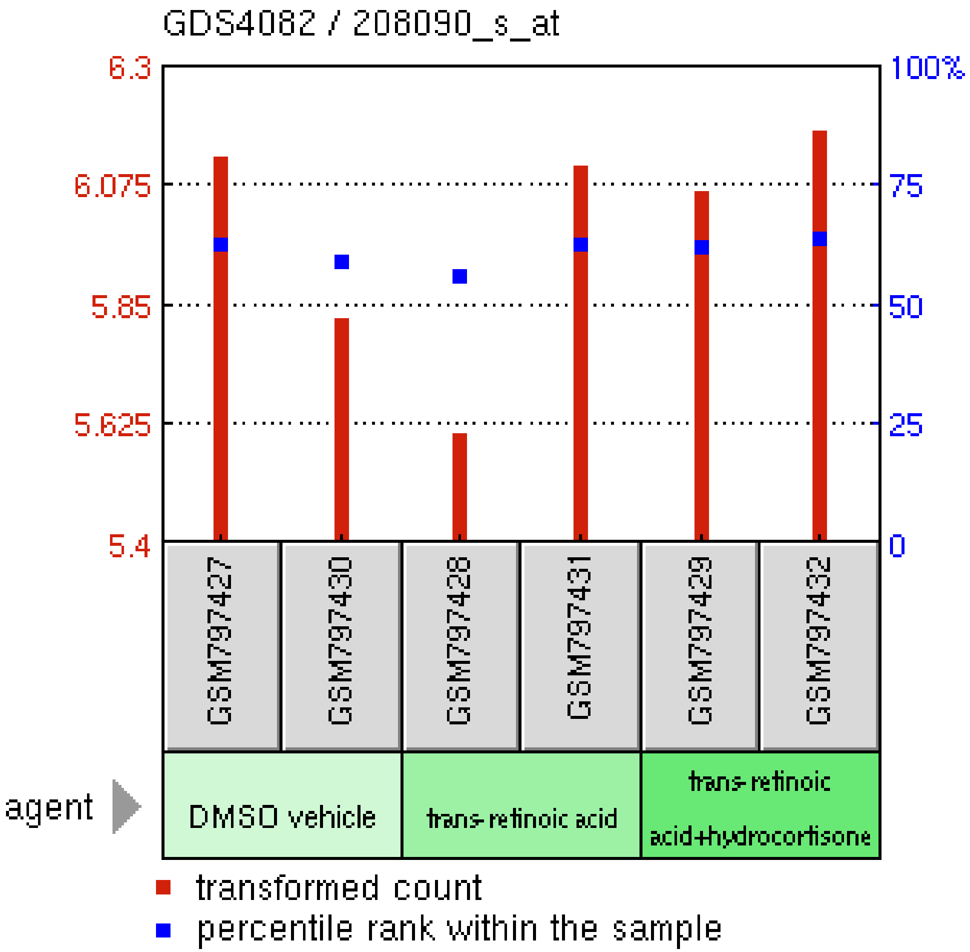

Sample	Title	Value
GSM797427	MCF-7 cell_DMSO_12hr_biological rep1	6.1288
GSM797430	MCF-7 cell_DMSO_12hr_biological rep2	5.82699
GSM797428	MCF-7 cell_tRA_12hr_biological rep1	5.60963
GSM797431	MCF-7 cell_tRA_12hr_biological rep2	6.11376
GSM797429	MCF-7 cell_tRA-H_12hr_biological rep1	6.06219
GSM797432	MCF-7 cell_tRA-H_12hr_biological rep2	6.17603

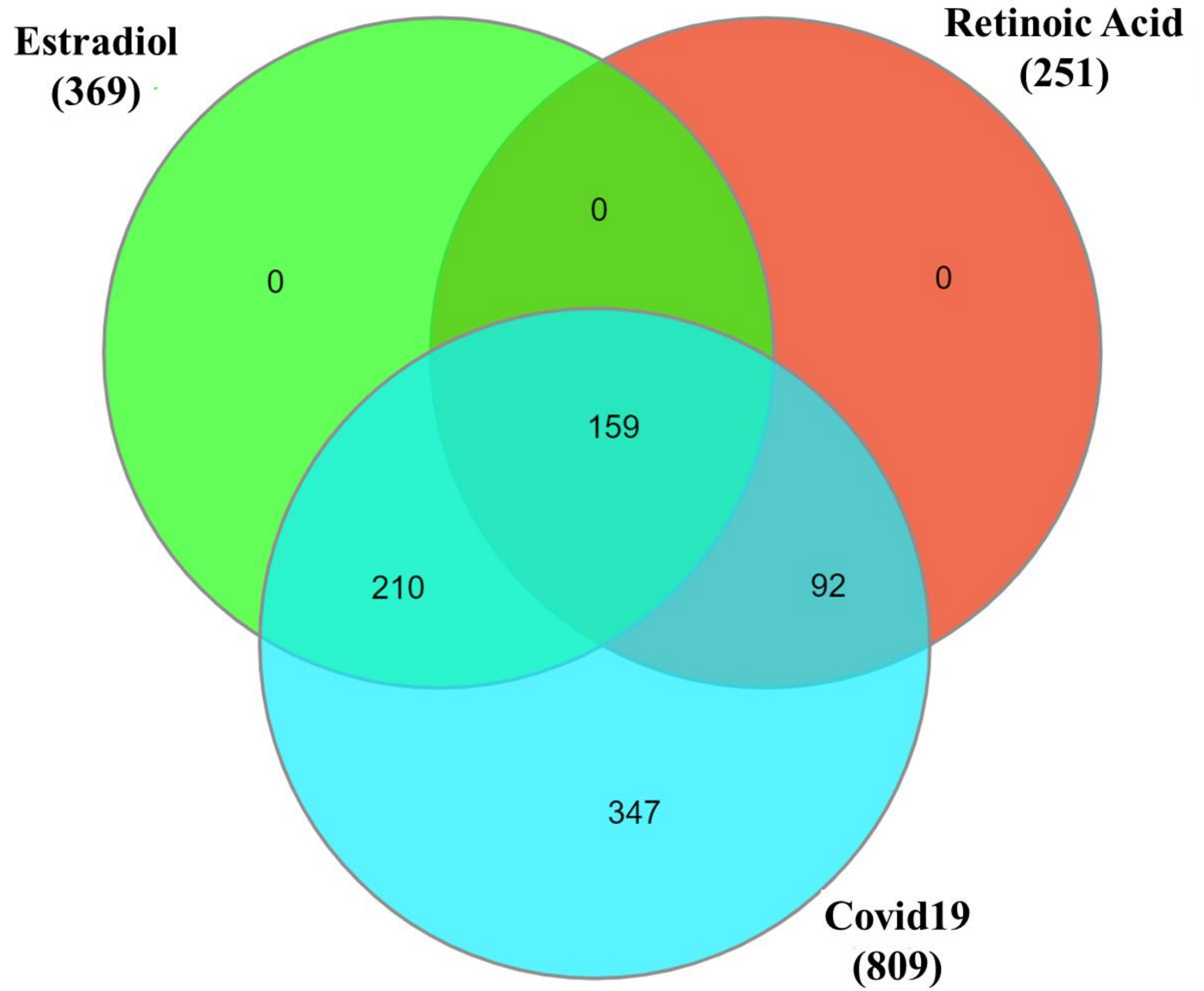

Supplementary Figure 15. Bipartite combination of estradiol and retinoic acid target 56% of human proteins prey for SARS-CoV-2 proteins and interfere with the functions of all but one SARS-CoV-2 proteins..

Supplementary Figure 16. Identification of miRNAs regulating the expression of TMPRSS2 and CTSB/L. The miRNA, hsa-miR-379 increased upon infection with influenza A infection in dendritic cells and in peripheral blood of Parkinson’s disease patients, indicating a candidate miRNA to target.

TargetScan microRNA 2017

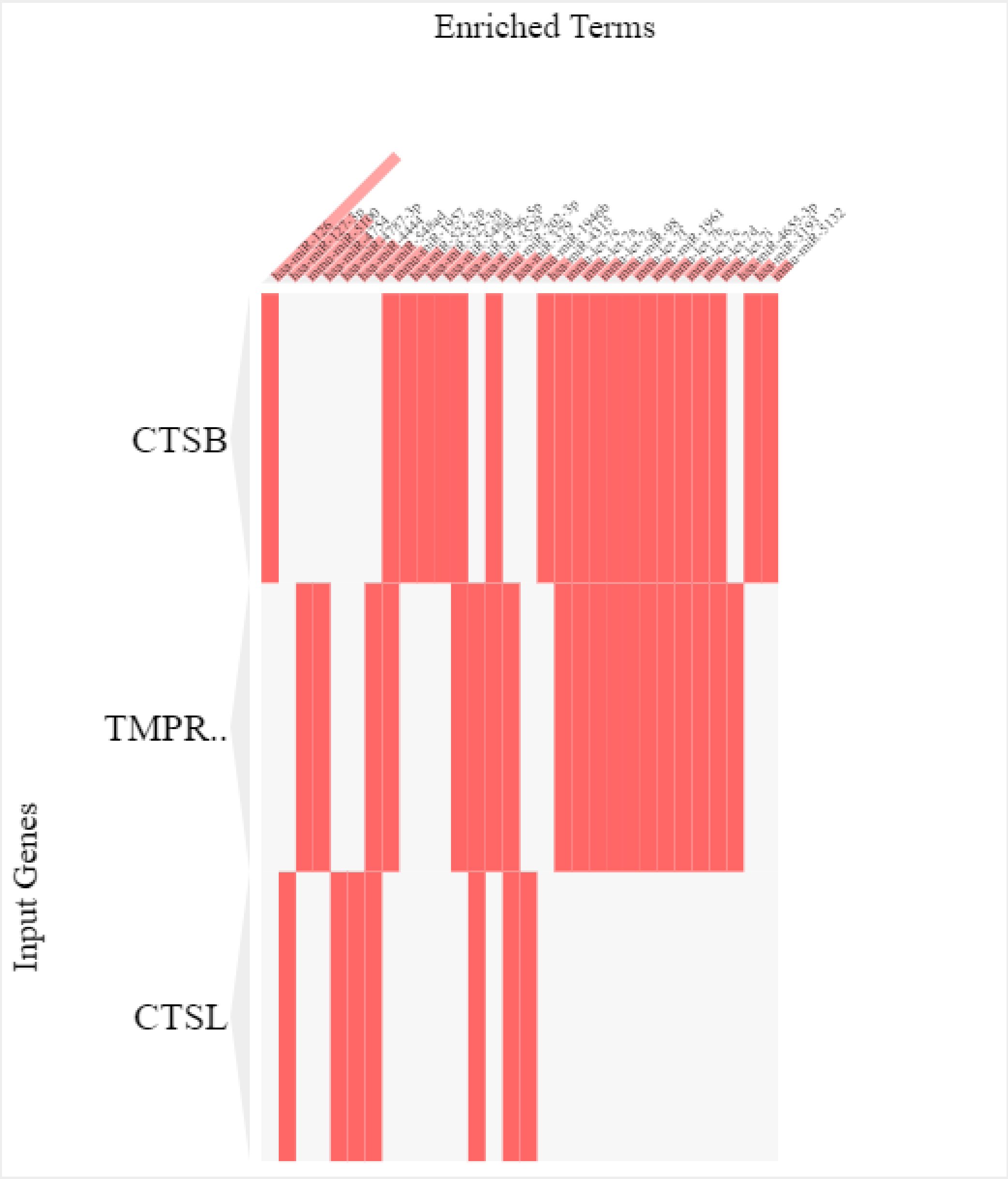

MIR379 - Influenza A effect on plasmacytoid dendritic cells

**Profile**	GDS6063 / ILMN_3309769
**Title**	Influenza A effect on plasmacytoid dendritic cells
**Organism**	Homo sapiens

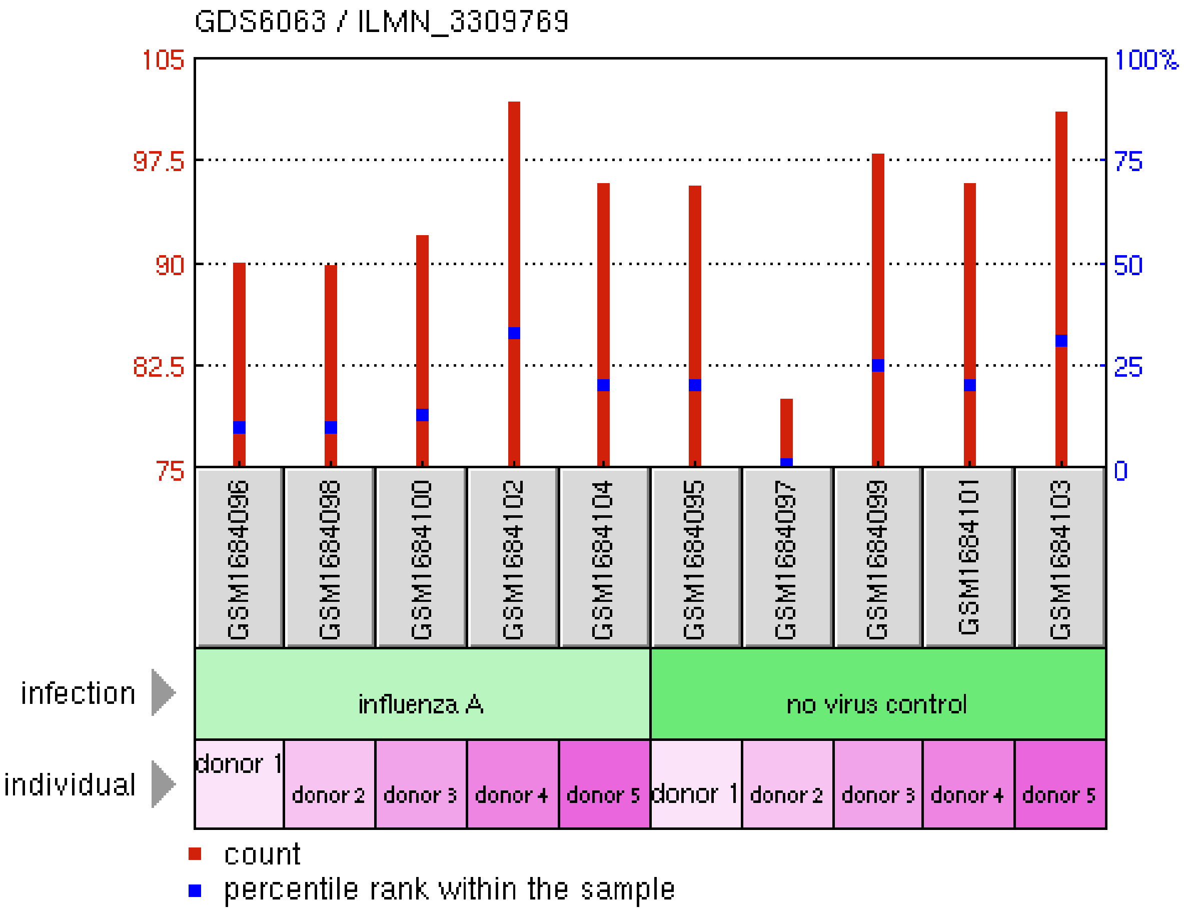

Sample	Title	Value
GSM1684096	Donor 1 - Influenza treated - 8h	90.0956
GSM1684098	Donor 2 - Influenza treated - 8h	89.9721
GSM1684100	Donor 3 - Influenza treated - 8h	92.1566
GSM1684102	Donor 4 - Influenza treated - 8h	101.814
GSM1684104	Donor 5 - Influenza treated - 8h	95.8511
GSM1684095	Donor 1 - No virus control - 8h	95.7292
GSM1684097	Donor 2 - No virus control - 8h	80.1677
GSM1684099	Donor 3 - No virus control - 8h	97.9748
GSM1684101	Donor 4 - No virus control - 8h	95.798
GSM1684103	Donor 5 - No virus control - 8h	101.084

MIR379 - Early Parkinson's disease: peripheral blood

**Profile**	GDS5646 / ILMN_3309769
**Title**	Early Parkinson's disease: peripheral blood
**Organism**	Homo sapiens

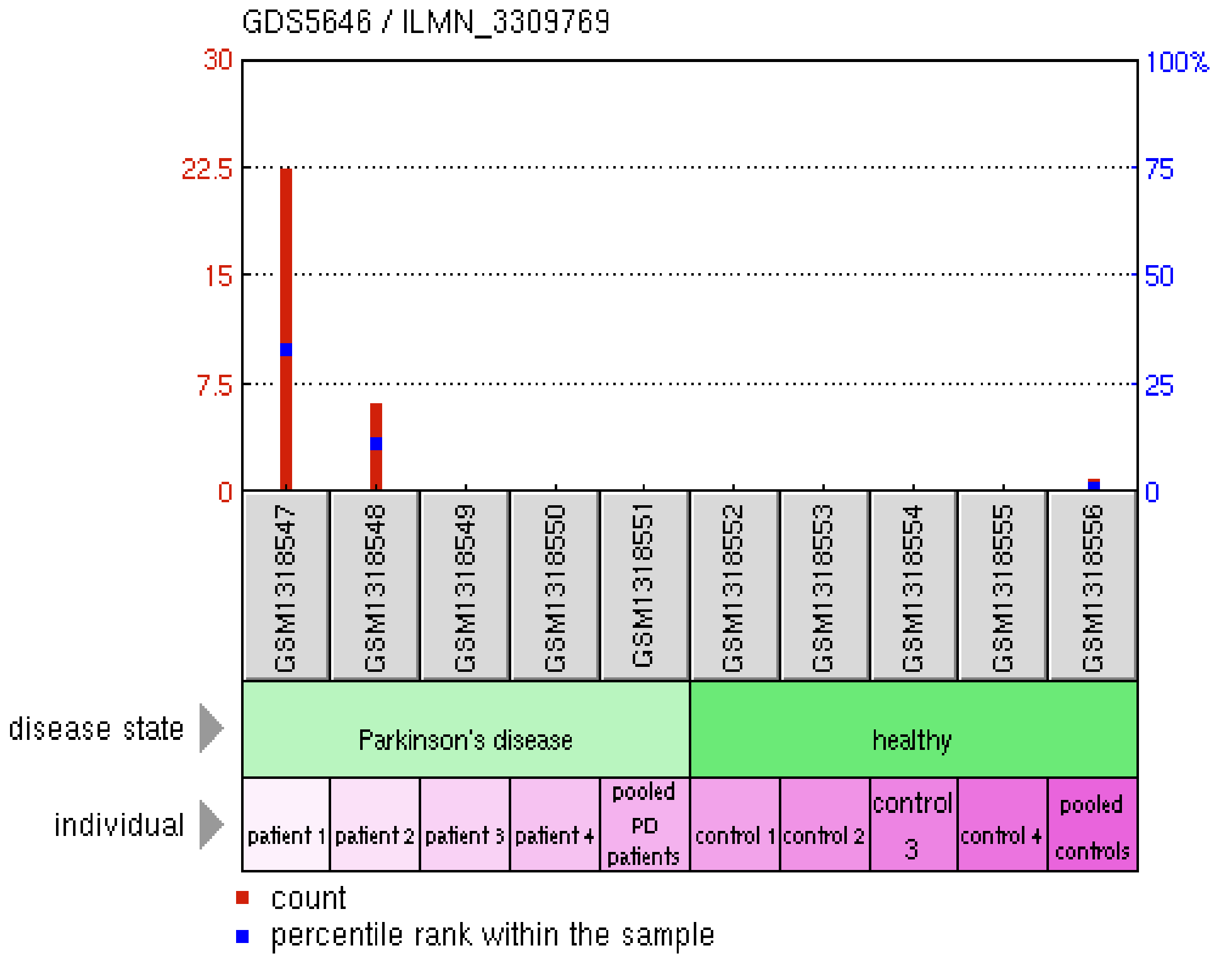

Sample	Title	Value
GSM1318547	PD patient 1	22.55
GSM1318548	PD patient 2	6.24
GSM1318549	PD patient 3	
GSM1318550	PD patient 4	
GSM1318551	RNA pool from PD patients 1-5	
GSM1318552	Healthy control 1	
GSM1318553	Healthy control 2	
GSM1318554	Healthy control 3	
GSM1318555	Healthy control 4	
GSM1318556	RNA pool from PD controls1-5	0.93
